# The experience of living with vitiligo in Nigeria: A participatory Interpretative Phenomenological Analysis

**DOI:** 10.1177/13591053241261684

**Published:** 2024-07-30

**Authors:** Nick Taylor, Ogo Maduesesi, Vasilis S Vasiliou, Andrew R Thompson

**Affiliations:** 1 The University of Sheffield, UK; 2 Unmind, UK; 3 Vitiligo Support and Awareness Foundation, Nigeria; 4 Cardiff University, Wales, UK; 5 Royal Holloway, University of London; 6 Cardiff and Vale University Health Board, Wales, UK

**Keywords:** Interpretative Phenomenological Analysis, psychodermatology, qualitative research, stigmatization, vitiligo

## Abstract

Vitiligo is a visible depigmenting skin condition, particularly noticeable on Black skin. There is widespread misunderstanding of the condition. Using a participatory form of Interpretative Phenomenological Analysis (IPA), we conducted eight semi-structured interviews with Nigerians living with vitiligo. Participants described their initial attempts to understand the condition, which typically drew on both traditional illness beliefs, religious influences, and the biomedical disease model. All participants reported experiencing marked stigmatization and discrimination. Participants experienced distress associated with thoughts about the personal meaning of the disease including its impact on their appearance and from concerns about anticipated and direct discrimination. Despite the wide-ranging impact, the participants’ narratives also contained references to the development of strategies that maintained wellbeing. This study provides valuable insights into the role of faith and traditional beliefs in both the experience and management of vitiligo in Nigeria. These insights can be used to develop individual and community interventions.

## Introduction

Vitiligo is a chronic autoimmune skin condition characterized by the loss of pigment from the skin and hair, leading to the development of noticeable white patches. While vitiligo affects individuals regardless of skin tone, it is more noticeable in individuals with darker skin (Bergqvist and Ezzedine, 2020). The prevalence of vitiligo varies among different regions, with estimates suggesting rates of 1% in Europe and higher rates, around 3%, in African countries (Onunu and Kubeyinje, 2003; Whitton et al., 2015). These variation in prevalence probably reflect reporting bias associated with the visibility or/and cultural issues linked with the condition (Birdi et al., 2020; Parsad et al., 2003) that have not been well examined.

A number of meta-analyses and meta-syntheses demonstrate that both children and adults living with a range of visible skin conditions are at risk of experiencing significant psychological distress and psychiatric co-morbidities including experiencing symptoms typical of depression, generalized anxiety, social anxiety, and feelings of shame and anger (Ablett and Thompson, 2016; Birdi et al., 2020; Ezzedine et al., 2021; Kussainova et al., 2020; Lukmanji et al., 2021; Osinubi et al., 2018; Van Dalen et al., 2022). Evidence suggests that studies using questionnaire measurements of depression and anxiety typically report higher levels of affective distress than studies using clinical interview (Osinubi et al., 2018). Nevertheless, there is clear evidence that people diagnosed with vitiligo are at heightened risk of developing clinically significant mood disturbance (Eleftheriadou et al., 2024; Osinubi et al., 2018; Thompson et al., 2022). Indeed, in a recent large-scale UK population-based cohort study that examined medical records, Thompson et al. (2022) reported that vitiligo is associated with an approximately 25% increase in risk of new onset depression and anxiety, within the first 2 years following the diagnosis. This study also found evidence that the risk of psychological co-morbidity was higher in people of non-White ethnicity. Further, people with both vitiligo and mental health comorbidities had more primary care encounters and were found to be twice as likely to have time off work as compared to matched controls. This indicates that vitiligo can have a profound impact on healthcare utilization, quality of life, and financial stability. Treatment for both the condition itself and its comorbidities has been identified from another large-scale record review study to be slow or/and lacking (Eleftheriadou et al., 2024).

A number of cross-sectional studies further demonstrate that the psychological and quality of life burden is common across different countries (Kussainova et al., 2020; Olasode and George, 2008; Yaghoobi et al., 2011). For example, Parsad et al. (2003) conducted a review of the quality of life of people in India and found that social stigma was a major concern. Studies conducted in Nigeria report a similar impact particularly in relation to stigmatization, yet it remains unclear as to the factors that contribute to this stigma or to its exact nature or phenomenology (Nnoruka, 2005). Stigmatization may exist due to a number of factors including misunderstanding about the condition, to concerns relating to its impact on appearance. For example, Ramam et al. (2013) reported that in India, the condition is often misinterpreted as “white leprosy,” and this has also been reported to be the case in Nigeria (Onunu and Kubeyinje, 2003). Further in a cross-sectional survey conducted by Tsadik et al. (2020) in Ethiopia, participants reported that supernatural explanations remained associated with vitiligo.

In the UK Thompson et al. (2010) conducted a template analysis with British South Asian women, and whilst they did not directly ask about cultural issues, the interview data revealed that stigmatization was associated with a range of factors including perceived impact on arranged marriage and family status. In an early interpretative phenomenological study conducted in the UK, Thompson et al. (2002) reported that their female participants were concerned by thoughts of being different, and attempted to avoid stigmatization by utilizing a range of internal and external strategies. A phenomenological study conducted with women with vitiligo in Iran also demonstrated that the impact on appearance was related to culturally specific concerns, and in particular concerns about marriage (Borimnejad et al., 2006). Further, the participants gave descriptions that other people had misattributed their vitiligo as resulting from punishment from God, uncleanliness, or dietary factors, and whilst such factors have been identified in other countries the nuances of such issues appear to vary depending on cultural, geographical, and temporal factors.

Despite the wide use of qualitative methods in health and illness it is surprising that there has been so little research with vitiligo. Thus far, only the small number of qualitative interview based studies previously described have investigated the experience of living with the condition. These have all been with female participants and varied in quality, and have tended to not consider reflexivity or have significant expert by experience involvement beyond participation. Lack of expert by experience involvement is a particularly important deficit as it is likely to hinder understanding of the sociocultural aspects of living with a condition like vitiligo.

As yet there have not been any substantive qualitative investigation conducted in Africa, and given this the present study sought to understand the psychosocial impact of living with vitiligo in Nigeria. In addition to the general need for expert by experience involvement, when researchers are working in cultures different from their own they must ensure that the methodology used is sensitive to cultural issues, and this is particular important when investigating phenomenon related to ethnicity such as skin pigmentation. Consequently, in this study we used a participatory form of Interpretative Phenomenological Analysis (IPA: Larkin and Thompson, 2012; Smith et al., 2021). Interpretative Phenomenological Analysis (IPA) involves a double-hermeneutic process whereby the experiences of the participant are first interpreted and made sense of by themselves within the context of the study aims, and secondly interpreted and made sense of by the research group (Larkin and Thompson, 2012; Larkin et al., 2006; Smith et al., 2021). Further IPA is an idiographic case-series approach, and as such is ideally suited to capturing detailed accounts that can be interpretated in an attempt to understand stigmatization and distress associated with vitiligo within the sociocultural context in which it occurs.

## Method

### Study design

This study followed the well-established original IPA approach (Larkin and Thompson, 2012; Smith et al., 2009). IPA is grounded in two core theoretical foundations: phenomenology and hermeneutics. It begins by constructing a descriptive account of the experiences, and then moves toward making grounded interpretations based on those accounts. The IPA approach sought to not only give voice to a Nigerian account of the experience of living with vitiligo, but also sought to provide interpretation that goes beyond the accounts themselves, incorporating psychological concepts so as to enhance understanding (Smith et al., 2009). This approach has previously been used to investigate the experiences of living with vitiligo by the senior author in other contexts (Moss et al., 2020; Thompson et al., 2002, 2010). Further IPA has also been used widely in Clinical Health Psychology field (Smith, 2010; Thompson, 2017).

In researching groups according to socially constructed definitions of race/nationality/ethnicity it is essential to work with experts by experience so as to avoid “essentialism” (Gunaratnam, 2003). The participatory approach was guided by Emanuel et al.’s eight-point guideline for research in developing countries (Emanuel et al., 2004: See Supplemental Material). As such this study used a participatory form of IPA as advocated by Larkin and Thompson (2012) to capture and reflect upon the principal claims and concerns of individuals living with vitiligo in Nigeria. The study was an active collaboration with the Nigerian vitiligo charity (VITSAF: The Vitiligo Support and Awareness Foundation), and in addition had the support of the University of Sheffield’s West African Regional office, and the Nigerian Association of Dermatologists. The study was conceived and developed with VITSAF and in particular the third author who had made contact with the senior/last author to initiate and seek funding to support this work.

Ethical approval was gained from the University of Sheffield and guidelines for conducting research in developing countries was followed (Emanuel et al., 2004). Prior to interviews being conducted all participants provided written consent and had the chance to ask questions about the study.

### Sampling and recruitment

A small homogeneous sample of eight participants (see the Supplemental Material) were purposively recruited and reimbursed for travel expenses. Participants were included if they had a diagnosis of vitiligo, experienced some source of burden due to their condition, and spoke English. To assess the burden of the condition criterion, we used the Dermatology Quality of Life Index (DLQI: Finlay, 1997) and the Fear of Negative Evaluation questionnaire (FNE: Stopa and Clark, 2001). Both male and females were eligible to participate, and the exclusion criteria were loss of pigmentation due to reasons other than vitiligo or other significant health conditions. Recruitment occurred via social media and via the personal communication carried out by VITSAF. In order to participate some participants traveled from other regions within Nigeria.

### Data collection

A semi-structured interview schedule (see the Supplemental Material) was used. Face-to-face interviews were conducted at either a hotel or the VITSAF offices in Lagos, Nigeria. The interviews were conducted by the first and final authors, with assistance from the third author where necessary to facilitate communication via consideration of language and cultural nuances. Six out of eight interviews involved the third author being present. The lead interviewers were a trainee clinical psychologist (first author) and a registered clinical health psychologist (senior/final author), who were experienced in conducting sensitive interviews. The interviews lasted 1–2 hours on average and were recorded and stored on a password-encrypted laptop. The subsequent transcripts were stored securely in an encrypted data repository. Participants received a small but sufficient payment provided in local currency to support incidental expenses.

### Data analysis

We followed guidelines to conducting IPA as per the original approach (e.g. Eatough and Smith, 2008; Larkin and Thompson, 2012; Smith et al., 2009). For each participant the transcribed audio recordings were checked for accuracy by the research team drawing on the third author for assistance where cultural translation was necessary. During the reading of the transcripts the first author referred to a reflective diary that had been maintained by the first and final authors throughout the research process. This was used to facilitate understanding of the phenomenon being investigated with reference to the researchers’ own experiences and positions (see reflexivity section below: Biggerstaff and Thompson, 2008; Shaw, 2010).

Data from the transcriptions was organized by the first author into a spreadsheet according to preliminary descriptive themes generated from reading notes that had been made in the left-hand column of each transcript. Once an individual transcript was coded the themes were organized into clusters via returning to the original text excepts. To make the early super-ordinate themes and sub-themes more visually accessible to interpretation, mind-maps showing connections between clusters of themes were produced using software. This process was repeated for each participant before group themes were created via careful consideration of the original individual data sets. The development of the overarching group superordinate themes involved repeated checking back to the raw data as held in the spreadsheet. As such the entire analysis was a cyclical rather than linear process and involved frequent discussions and checking of data by the author group primarily led by the first and final author.

### Reflexivity

Due to the interpretative nature of IPA reflexivity is central to the research process (Biggerstaff and Thompson, 2008; Shaw, 2010). As described above the first and senior/final author kept an ongoing reflective diary. This was used to record both theoretical reflections and field notes at various points throughout the study. Within the diary particular consideration was given to the impact of the research teams preconceptions might have on the study’s findings, and how the research teams values and theoretical orientations might affect the study. The reflexivity process was essentially used to inform the analysis process via consideration of the inter-subjectivity and some excerpts are included in the results section to demonstrate this (Biggerstaff and Thompson, 2008).

### Quality control

Qualitative equivalent of validity criteria as defined by writers such as Stiles (1999) were adhered to, which for example included consideration that the analysis was conducted in an iterative fashion. As IPA is an interpretative approach, participant validation was not utilized and was replaced with an audit process that saw the data and analytic processes scrutinized so as to ensure that there was evidence of there being quality data, and that this data could be evidenced as having been analyzed in a rigorous fashion commensurate with IPA procedures (Larkin and Thompson, 2012; Spencer and Ritchie, 2012).

### Data sharing statement

All the individuals’ participant data collected during this study were first anonymized and then was uploaded on Figshare. The current article includes the complete raw dataset collected in the study including the participants’ dataset we used for analyses. All the data files are available in the Figshare repository and accessible as Supplemental Material via the Sage Journals platform.

## Results

The results demonstrate that there is a high level of social stigma attached to living with vitiligo in Nigeria for the included participants. Traditional beliefs clearly merged alongside medical information that had been provided to participants in shaping the participants understanding of the cause of the condition, options for treatments, and concerns as to how others would judge them. Adjustment was hard to develop in the face of limited access to medical support and high perceived and apparently actual experiences of social ostracism and discrimination. Nevertheless, the participants described developing a range of coping strategies, and these were bolstered by the acceptance provided by peer support and personal faith.

The careful grouping of themes across the transcripts led to the creation of four superordinate themes, each associated with secondary themes (see Table 1). These are described in more detail below with some references also being made to the reflective diary used to support the development of the interpretations.

**Table 1.** Summary of themes (IPA analyses).

IPA themes
1. Discovering vitiligo 1.1. Initial psychological reactions 1.2. Initial reactions from family and friend 1.3. Attempts to appraise the onset of vitiligo
2. Core beliefs about causation 2.1. Idead deriving from traditional beliefs of the condition 2.2. Ideas deriving from medical beliefs of the condition
3. Stigma and its emotional burden 3.1. Experiencing stigma 3.2. The emotional burden of vitiligo
4. Learning to live with vitiligo in Nigeria 4.1. Developing acceptance 4.2. Spiritual support 4.3. Behavioral coping 4.4. Positive role of user-led support

Sole numbers present the super-ordinate themes and indexed numbers their generated sub-themes.

### Discovering vitiligo

The first super-ordinate theme generated three overlapping sub-themes, including: (a) initial psychological reactions; (b) attempts to appraise the onset of vitiligo; and (c) reactions from family and friends.

#### Initial psychological reactions

Initially, the participants described having no awareness of what was happening to their skin and described feeling anxious:

P1: Initially, I felt that it was a reaction of some of the things I eat, so I had to do away with all of the things I was eating – and then it didn’t stop.

Many participants attempted to ignore the depigmentation in the hope that it would go away. This quickly led to a realization that it was not improving and for some people it was spreading:

P1: I ignored it, thinking it was one of those reactions. Then it came to my forehead, then another part. So, at that point … okay, I think something is wrong

#### Initial reactions from family and friends

Participants described their friends and family expressing fear and anxiety and often misattributing the condition to other conditions. They also worried that bad spirits or a curse had caused it. This confusion appeared to run in parallel to the participants’ own reaction:

P1: They (family) were kind of scared … so worried and they believed that it must be some spiritual thing, it must be some nemesis, and we have done something – so many things that they believe

In the quote above P1 demonstrates awareness of the numerous traditional beliefs that family were drawing on to understand the vitiligo, and the quote indicates ambivalence about this demonstrating dual internalization of both medical and traditional beliefs.

Christianity was advocated by most of the participants’ families and friends as likely to be needed to treat the condition. Again there was ambivalence about this being useful, and this at times caused conflict within the family:

P6: Me, I don’t want to follow him (Dad) out that we should be going to church, different type of church, so that any man of God can heal you

#### Attempts to appraise the onset of vitiligo

Related to this the participants described having held and still holding a wide range of explanatory hypotheses about causation of their vitiligo. Many described holding beliefs at some point that the vitiligo might have been caused by something eaten, or the result of wronging some spirit, or the result of a curse:

P7: I never knew what was going on and what was wrong with me … I was scared … Initially I believed that vitiligo was caused by a spell because I have not seen it before.

None of the participants continued to believe that spirits or traditional magic played a role in their vitiligo, however they were aware that others might well think this.

Reflexivity: *I found it hard to talk adequately about traditional beliefs due to my ignorance. Some participants described eating certain foods as being against tribal cultures. I became confused about diet being linked to traditional spiritual beliefs. To help clarify my own understanding of these issues I spoke with one of the founding board members of VITSAF.*

### Core beliefs about causation

The second super-ordinate theme, as Table 1 presents, generated two overlapping sub-themes, referring to: (a) ideas derived from traditional beliefs of the condition, and (b) ideas from medical beliefs of the condition.

#### Ideas derived from traditional beliefs of the condition

A central narrative throughout the transcripts was that vitiligo might be caused by offending forefathers, eating a forbidden food, or being cursed by someone:

P5: (the treatment is) like a sacrifice. They will appease this thing that maybe was the spirit that maybe the fore-father that they didn’t … that they didn’t appease … that is … now affecting maybe one of the grandchildren … I’ve tried it … (it) doesn’t work

Such was the strength of these beliefs that many participants had tried traditional treatments; however, none reported these interventions as having any lasting effect:

P3: I went (to the village), I saw a woman, and she prepared some things for me, one to drink and one to apply on my skin. She collected many things like leaves and other things … it worked … then, when I had my baby, the whole thing started again

Reflexivity note: *The participants varied in how they related to traditional beliefs. We wondered whether this was influenced by age, with the older participants having more exposure to “traditional ideas.” Perhaps people with stronger ties to their tribe and village might have more exposure to traditional beliefs. Another reflection was that vitiligo has no cure and no clear medical etiology; it is more liable to become the focus of traditional beliefs about illness. Further, there was occasional reluctance to speak about traditional beliefs, perhaps because we are from the UK and might be seen to not understand or be judgmental and consequently, we should discuss this with VITSAF.*

#### Ideas derived from medical beliefs of the condition

All the participants had some experience of receiving medical treatment or medical consultation and there was unanimous acceptance of it being a medical condition. This position was developed over time and occurred as a result of both contact with VITSAF, and peers with the condition, as well as via contact with doctors. Unfortunately, most participants’ had had some negative experience of contact with the medical community. They reported experiencing initial misdiagnosis, receipt of incorrect advice, and difficulty in receiving any advice on management once they had the diagnosis:

P7: Even now most doctors don’t want to know about it, that’s the issue, most doctors don’t know about it

However, as the participants became aware of the international issues related to lack of treatment for vitiligo via contact with VITSAF, there was a recognition that a lack of resources were contributing to the difficulties that the Nigerian medical community experienced in providing support and treatment:

P1: (the dermatologist was) not friendly at… She just looked at it, asked me a few questions, and just put down on the paper, vitiligo. I was trying to get her to tell me, okay, this is what you have, or something, but she never said it.

### Stigma and its emotional burden

The third super-ordinate theme focuses on the (a) experience of stigma and the (b) emotional burden associated with vitiligo, particularly when accompanied by stigmatization.

#### Experiencing stigma

All participants described experiences of stigma. Either an experience from an early young age or an ongoing one during their day-to-day lives. For example, two participants whose vitiligo had occurred when they were children reported having been bullied, causing them to feel shame:

P7: Well, it wasn’t so easy because … children back then that I’d play with tend(ed) to mock me.

Further, some participants reported other people worrying that the condition was contagious in some way:

P7: a lady walked away from … her food and left where we were sitting because I was there

This was clearly experienced as being instrumental in affecting social confidence:

P7: it made me to lose my self-confidence … It made me angry … I was annoyed (at strangers’ negative reactions), and so ashamed of myself.

Five participants reported experiencing discrimination in their jobs or difficulty securing a job in the first place. This was associated with feelings of both humiliation and shame. For example, P4 was extremely upset during the interview relaying how she had recently lost her job as a result of a complaint being made by a customer about her vitiligo:

P4: It does really affect my work … a lot of people would stop me … “boss, that is this one?”

Some participants reported that the vitiligo had not had an effect on their work, however this was more typically the case for participants working in roles with low contact with customers/clients. For example, two of the participants reporting lower impact at work were office workers and one was a sailor. For P7 who was a sailor, he described there being a tolerance of difference associated with many others having some form of difference and also because of a hint that any such discrimination might be dealt with aggressively:

P7: Well (it doesn’t impact on my work) because now I’m a sailor and where I work people don’t discriminate.

#### The emotional burden of vitiligo

All the participants described negative emotional reactions due to their condition.

P4: It makes me feel depressed at times, makes me feel kind of depressed

Part of the negative reaction appears around loss of identity. Participants described feeling different to those around them and touched upon losing their sense of belonging to their family:

P3: my skin is not the same with my siblings, my parents, my friends. Among them I look different

### Learning to live with vitiligo in Nigeria

The final super-ordinate theme generated four overlapping sub-themes, referring to: (a) developing acceptance; (b) spiritual support; (c) behavioral coping strategies; and (d) Positive role of user-led support.

#### Developing acceptance

The participants, unanimously, described slowly arriving at a point of acceptance of the vitiligo:

P2: It’s natural. My skin is who I am. It’s who I am.

This was often supported by acceptance of others. For example, participants who were married typically described their spouses as a source of support:

P2: But my wife accepted it and my children would even boast of my pink lips.

Indeed, all of the participants described the instrumental role played by friends:

P3: I have a friend and ever since vitiligo started she has always telephoned me … whenever I see her I’m so happy that she’s given me courage and boldness

Further, all the participants described the realization that they were not going to be able to find a cure. Giving up on this was seen as setting them free from searching for answers and from the resources that were required to do this. This process was important in developing acceptance and helping participants to develop a sense of living “in peace” with their vitiligo:

P8: everything that we are spending, it’s not working, so why am I using that? It’s no solution to the problem, so let me move forward

Participants appeared to be able to accept vitiligo by construing living with it as having helped them to develop into better people:

P2: Psychologically it made me a stronger person …I look at people now who have blemishes, deformities, with a lot of kindness and respect.

Reflexivity note: *The participants were generally presented as having high levels of acceptance and coping strategies. All were recruited through one source, VITSAF, and there is similarity in many of the answers. At times it felt like we were hearing the narrative of VITSAF. Perhaps belonging to this assertive support group had assisted them in developing a new shared identity, and indeed, one of the sub-theme indicated the common narrative. We need to be mindful of this by checking the accounts and also transparent in discussing limitations.*

#### Spiritual support

Participants mentioned the importance of faith in helping them to manage the condition. There was unanimous belief that God helped them to better cope with the condition:

P2: Some authority is behind this and I acknowledge that he is God, and as long as I acknowledge that he is God I have no problems … I mean if I hadn’t God’s grace I don’t think I would have coped

Further, participants spoke about the church congregation and how it provides them with a sense of community and belonging, a place where stigma was not present:

P6: People in the church … take me as their own brother and sister, they don’t even look if I have vitiligo at all

All participants also mentioned some personal beliefs and benefits, gained, from prayer:

P8: Yes, when I’ve used everything and I see that it is not working, we have to … pray again that God will intervene

Nevertheless, there were also some concerns as indicated earlier that there was a blurring between faith support and attempts at offering unsubstantiated treatments, both psychological and medical:

P1: Pastor, be a pastor. Leave a psychologist to be a psychologist, leave a psychiatrist to be a psychiatrist. If you want to be a psychologist, go and study that and add it to your pasturing. Stop misleading people.

#### Behavioral coping strategies

Participants described using a range of behavioral coping strategies. Firstly, all the participants gave examples of using avoidance and concealment as behavioral coping strategies that can diminish attention and perceived stigmatization of their vitiligo. These included covering affected areas with clothing and camouflage and not attending social events:

P6: It’s something like powder, that’s easy to cover my face, but you will not know that I have it in my face

Notably, none of the four men were currently using camouflage and all expressed an unwillingness to do so. One hypothesis for this could be that it is related to hegemonic masculine stereotypes:

P5: No, no, I’m not comfortable with using makeup

Further, the participants described having developed a range of coping strategies to manage other people’s reactions. The common theme among these naturalistic strategies was showing self-efficacy:

P5: But I realize people are looking at me, but I just have the confidence and I have to face what I’m doing

Finally, participants referred to certain practices, such as healthy eating and having mental plans of how to respond to rejection as supporting ones, as helpful addressing aspects of vitiligo:

P5: I use a supplement, you know? The supplement is a combination of … vitamin(s). I got it from America

#### Positive role of user-led support

The participants were recruited via a charity, and all described benefiting from peer support. Participants described it as giving them a sense of belonging and facilitated them in fighting stigmatization:

P2: (because VITSAF people) are able to fight the stigmatisation and the rejection in society

Participants stated that members of VITSAF would be able to guide them by acting as role models and providing confidence:

P4: Yeah, they have been there before. They can put me right, put me on the right track

Without exception participants spoke of a desire to promote vitiligo within society. There was a shared desire to help others through the journey to acceptance and to challenge society’s stigma:

P4: I believe there are a lot of people who still have this vitiligo … who need help

## Discussion

Despite vitiligo being highly visible on darker skin, surprisingly there has been a dearth of in-depth qualitative research conducted in Africa. This is highly unfortunate as it is essential to develop a deep understanding of the psychosocial issues associated with vitiligo in populations and regions with high incidence rates, in order that culturally appropriate interventions can be developed.

The findings of this study provide some support for the applicability of current psychological theories and models of disfigurement (e.g. Clarke et al., 2013; Thompson, 2012a, 2012b), with the participants describing vitiligo as having both a negative psychological impact and considerable impact upon their quality of life. This fits with Thompson and Kent (2001) and Cash’s (1990) distinction that in order to understand disfigurement or visible difference, both “the view from the inside” (self-concept, quality of life, emotional wellbeing) and “view from the outside” (social and cultural interactions) must be understood. However, our findings provide a unique insight into the experience of people living with vitiligo in Nigeria that suggests existing models do need to give greater consideration to cultural aspects of the condition that go beyond simple recognition that this might be associated with stigmatization.

Whilst the findings largely align with previous qualitative (Borimnejad et al., 2006; Thompson et al., 2002, 2010) and survey studies (Ayonrinde et al., 2004; Nnoruka, 2005; Onunu and Kubeyinje, 2003) that highlight the common experience of early distress, stigmatization, and the desire to conceal the condition, there are important nuanced differences that will be discussed below. Certainly, the experience of stigmatization is evident in both the current study and research conducted in other countries (Kim et al., 2009; Olasode and George, 2008; Onunu and Kubeyinje, 2003; Parsad et al., 2003; Schmid-Ott et al., 2007), with the participants’ in this study reporting having experienced particularly vocal public displays of disgust and shock associated with misunderstandings and prejudice about vitiligo.

The psychological impact of vitiligo was described as initially being distressing and confusing. When compared with the findings of Thompson et al. (2002, 2010) where the participants were drawn from the UK, the participants in this study described having less initial awareness and knowledge about vitiligo, and this is similar to the findings of Borimnejad et al. (2006) with a sample of Iranian women. One explanation for this difference might be the differences in the health care systems, however there is also the possibility that additional confusion and anxiety results from the increased complexity, associated when alternative (traditional or faith-based) causal explanations are present. Indeed, our superordinate theme associated with core beliefs about causation indicates narratives of confusion and distress in participants’ accounts.

Indeed, the present study uniquely highlights the significant role of traditional cultural health beliefs in the vitiligo experience. Participants’ descriptions of their initial beliefs about vitiligo corroborate those given by Ayonrinde et al. (2004) who explored the perspectives of lay individuals in Nigeria, of health issues. Notably, these beliefs are deeply rooted in traditional spiritual frameworks. Many beliefs are rooted in the idea of punishment for previous wrongdoing, such as displeasing forefathers or going against traditions. In turn, these beliefs can influence treatment approaches. For instance, some individuals may opt for evidence-based intervention, while others may seek cultural practices, for instance, involving interactions with tribe members, and in conditions where there is limited medical treatment available and lack of understanding about a condition, both approaches may be utilized, at least initially, as was found in this study.

Participants in this study emphasized the significant role of God and their own faith in helping them to positively adjust and cope. Substantial evidence indicates that positive religious coping is linked with better illness experience and outcome and that negative religious coping is associated with poor illness experience and outcome, specifically anxiety and depression (Krägeloh, 2011; Weber and Pargament, 2014). The narratives from all the interviews in our study consistently reflect the adoption of positive religious coping mechanisms by participants, albeit on occasion accompanied by ambivalence about specific practices suggested by others.

Likewise, all the participants described the benefits of belonging to a peer support organization as facilitating positive coping that allowed them to find out about treatment and benefit from the support of a community. Mead et al. (2001) argues that peer support is an extension of community rather than being an extension of professional style care. She defines peer support as involving respect, mutual agreement about what is helpful, and a shared responsibility. This definition fits well with the description given by participants of the benefits of connectedness with VITSAF, and this is the first study to highlight the potential benefits of peer support for vitiligo in Nigeria.

From a practical perspective, incorporating spiritual well-being alongside physical and mental well-being might provide a foundation for culturally sensitive interventions in African countries (Anakwenze, 2022). Likewise, accommodating traditional health beliefs into cognitive, behavioral, and public health interventions can booster targeted outcomes, such as reducing stigmatization and shame, as well as creating more inclusive healthcare environments that value and respect diverse beliefs and practices. Strategies such as cultural sensitivity (Resnicow et al., 1999), collaborative approaches (Wallerstein et al., 2017) education and awareness (Gamby et al., 2021), and the integration of spirituality (Karademas and Petrakis, 2009; Koenig et al., 2001; Tsaousis et al., 2013) can foster a greater understanding, challenge discrimination, and promote inclusive healthcare environments (Betancourt, 2003). However, it is essential to tailor interventions to the local context and engage with relevant stakeholders and communities to ensure their relevance and success in addressing the unique experiences of individuals with vitiligo in African countries (De Jong, 2014). Development and testing such interventions, may be particularly important for countries in African with strong cultural influence where ineffective and dangerous medicinal products or traditional treatments can prevail.

The findings of this study have important clinical implications for the management of vitiligo. The results suggest that socio-cultural understandings ought to be integrated in psychological interventions, targeting at alleviating distress associated with vitiligo, in combination with public health strategies that will raise awareness and challenge discrimination. Specifically, when working with people with vitiligo from African countries, it is essential to consider specific cultural factors. This may involve providing specialized training to practicing clinicians, including dermatologists and practitioner psychologists, to enhance their communication skills and improve patient interactions. Moreover, the study highlights the crucial role of peer support organizations in regions with limited access to medical care, such as Nigeria. These organizations can provide psychosocial support and other necessary care in an affordable way, thereby addressing the sparsity and expense of medical services. Lastly, the findings emphasize the importance of offering access to professional camouflage services as a means of assisting people to manage the reactions of others toward vitiligo. By providing access to such services, individuals can better manage the visible effects of vitiligo and improve their overall well-being. These implications underscore the need for comprehensive approaches that combine psychological interventions, public health strategies, and access to supportive resources to effectively address the challenges faced by individuals with vitiligo.

The study acknowledges limitations, with participants being recruited via a specific organization, possibly limiting the themes transferability beyond this context. High acceptance of medical causes by our sample perhaps indicates access to information facilitated by the charity that others might not have, and clearly further studies are needed to examine the experience of participant samples in both Nigeria and other African countries not connected with peer support organizations. Whilst the study had clear processes in place to consider cultural issues, further studies might adopt additional ethnographic methods that might enable additional cultural issues to be identified. We used the original version of IPA and it is possible that the newer version (e.g. Smith and Nizza, 2022; Smith et al., 2021) may have possibly contributed to the development of more detailed themes. That said, our approach was extremely rigorous and had a high level of expert by experience involvement.

In conclusion, this unique qualitative study investigated the psychological, social, and cultural experiences of individuals living with vitiligo in Nigeria. The findings reveal that vitiligo creates confusion for both the affected individuals and their support networks, and highlights that there remains significant social stigma associated with the condition in Nigerian society. Participants accounts demonstrate the importance that religion, peer support, and personal resilience play in coping with the condition. Further, the study provides suggestions on how researchers and clinicians should leverage biopsychosocial frameworks to develop culturally sensitive interventions.

## Supplemental Material

sj-docx-1-hpq-10.1177_13591053241261684 – Supplemental material for The experience of living with vitiligo in Nigeria: A participatory Interpretative Phenomenological Analysis Supplemental material, sj-docx-1-hpq-10.1177_13591053241261684 for The experience of living with vitiligo in Nigeria: A participatory Interpretative Phenomenological Analysis by Nick Taylor, Ogo Maduesesi, Vasilis S Vasiliou and Andrew R Thompson in Journal of Health Psychology

sj-docx-2-hpq-10.1177_13591053241261684 – Supplemental material for The experience of living with vitiligo in Nigeria: A participatory Interpretative Phenomenological Analysis Supplemental material, sj-docx-2-hpq-10.1177_13591053241261684 for The experience of living with vitiligo in Nigeria: A participatory Interpretative Phenomenological Analysis by Nick Taylor, Ogo Maduesesi, Vasilis S Vasiliou and Andrew R Thompson in Journal of Health Psychology

sj-docx-3-hpq-10.1177_13591053241261684 – Supplemental material for The experience of living with vitiligo in Nigeria: A participatory Interpretative Phenomenological Analysis Supplemental material, sj-docx-3-hpq-10.1177_13591053241261684 for The experience of living with vitiligo in Nigeria: A participatory Interpretative Phenomenological Analysis by Nick Taylor, Ogo Maduesesi, Vasilis S Vasiliou and Andrew R Thompson in Journal of Health Psychology

sj-docx-4-hpq-10.1177_13591053241261684 – Supplemental material for The experience of living with vitiligo in Nigeria: A participatory Interpretative Phenomenological Analysis Supplemental material, sj-docx-4-hpq-10.1177_13591053241261684 for The experience of living with vitiligo in Nigeria: A participatory Interpretative Phenomenological Analysis by Nick Taylor, Ogo Maduesesi, Vasilis S Vasiliou and Andrew R Thompson in Journal of Health Psychology

sj-docx-5-hpq-10.1177_13591053241261684 – Supplemental material for The experience of living with vitiligo in Nigeria: A participatory Interpretative Phenomenological Analysis Supplemental material, sj-docx-5-hpq-10.1177_13591053241261684 for The experience of living with vitiligo in Nigeria: A participatory Interpretative Phenomenological Analysis by Nick Taylor, Ogo Maduesesi, Vasilis S Vasiliou and Andrew R Thompson in Journal of Health Psychology
