# Supplementary material for: The experience of living with vitiligo in Nigeria: A participatory Interpretative Phenomenological Analysis
Source: J Health Psychol. 2024 Jul 30;30(5):1120–35. doi: 10.1177/13591053241261684 (PMC11977828; doi:10.1177/13591053241261684)

Vitiligo study

Data analyses (robustness analyses)

**Process of data analyses**

Line-by-line the transcriptions were organized into a spreadsheet according to preliminary-themes (see explanatory memo). These themes attempted to capture the essence of the interview and the researchers’ interpretation of it. Once the entire transcript was coded into the spreadsheet the themes were reorganized into clusters. These clusters resulted in super-ordinate themes. So as to make the super-ordinate themes and sub-themes more visually accessible the spreadsheet was converted into a mind-map (see below). Finally, eight spreadsheets and mind-maps were brought together and connections were made between and within participants. The themes were then reorganized taking into account frequency, prominence and appropriateness. This process was aided through the use of a flow diagram (see below). The entire analysis was a cyclical rather than linear process. Previous steps were checked throughout to ensure the interpretations remained true to the original data.

Ethical and cultural considerations. Ethical approval was granted from the University of Sheffield via the Psychology Research Ethics Application Management System (PREAMS). Emanuel, Wendler, Killen and Grady (2003) outlined an eight-point guideline for conducting research in developing countries (see below); this guided the research. In researching groups according to socially constructed definitions of race/nationality/ethnicity it is important to give consideration to the meaning of the distinction being drawn and to avoid ‘essentialism’. That is the temptation to ‘imply an internal sameness and external difference or otherness’ to the group of people (Gunaratnam, 2003, p.29). The current research does not aim to uncover the ethnic or racial Nigerian experience for this would be an impossible task.

Quality control. To ensure research rigour an audit of the analysis process was conducted for both the IPA and the TA approaches (Larkin & Thompson, 2012). The first co-author reviewed the analysis process for three participants chosen at random. This involved reading through the transcripts, the initial coding, and examining the evidence for the final set of themes. Any disagreements were resolved through discussion. This helped ensure that the interpretations made were the most comprehensive available (Stiles, 1999). Research protocols were checked by VITSAF and representatives from the University of Sheffield Lagos office for comments on cultural sensitivity and appropriateness. Due to the interpretative nature of IPA reflexivity was central to the research process. This acknowledges that the researcher is a subjective rather than objective part of the process.

Reflexivity. Due to the primary methodology being IPA, an interactive, exploratory and interpretative approach, consideration of reflexivity is important. The researcher kept an ongoing reflective diary. This was used to record feelings and thoughts at various points through the study. Within the diary particular consideration was given to what impact the role and presence of the researchers had on the study’s findings and how the researchers’ values and theoretical orientations affected the study (Spencer & Ritchie, 2012). This diary helped to inform the analysis process; when using IPA methodology it is important to consider inter-subjectivity (Thompson & Harper, 2012). Where appropriate exerts from the diary are presented in the results. In addition to the diary the researcher attempted to avoid making cultural assumptions that might have negatively impacted on the interpretations.

1.
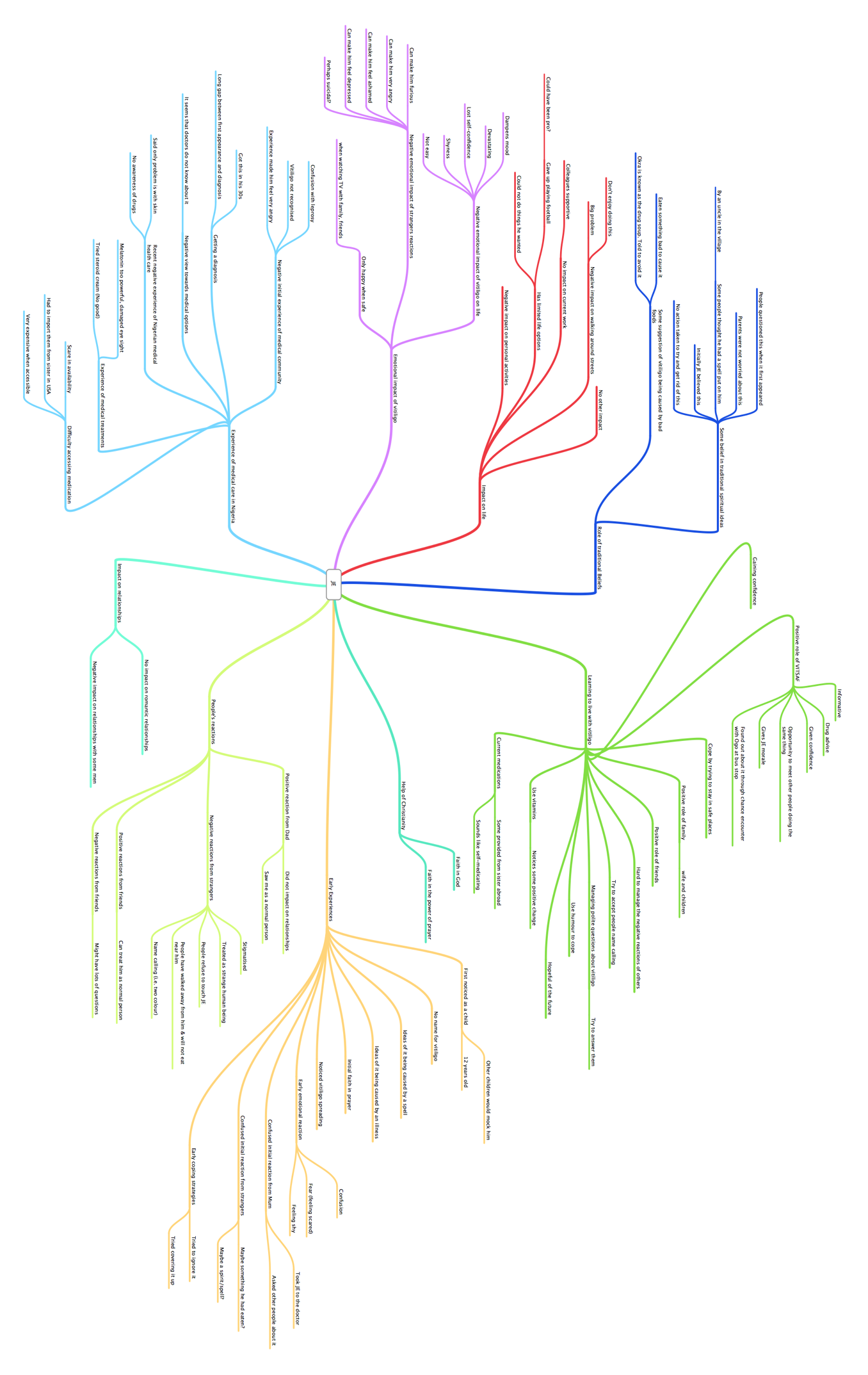
Example of mind map of IPA themes
2.
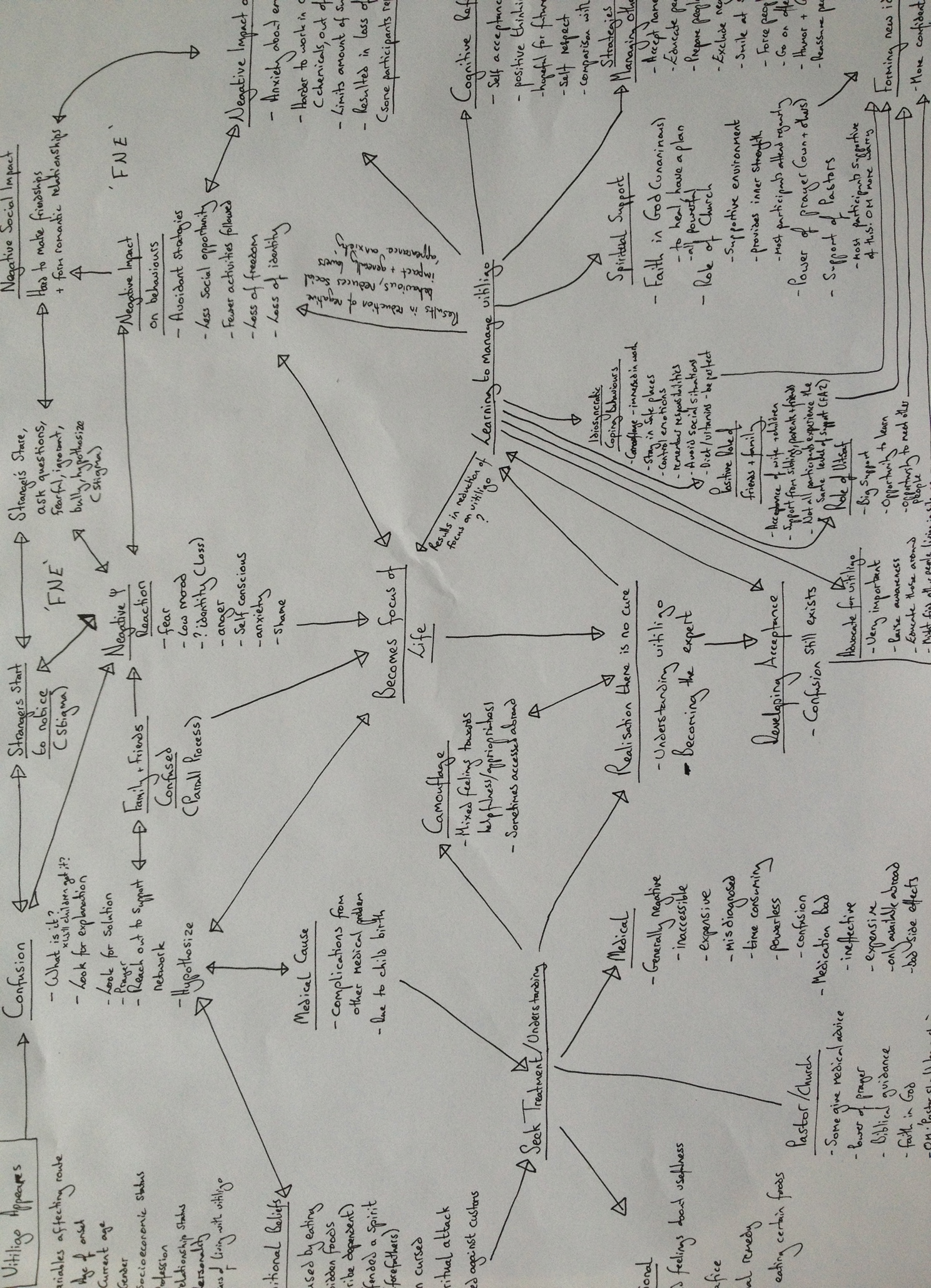
Flow diagram used to facilitate IPA working
3. Example of TA initial coding


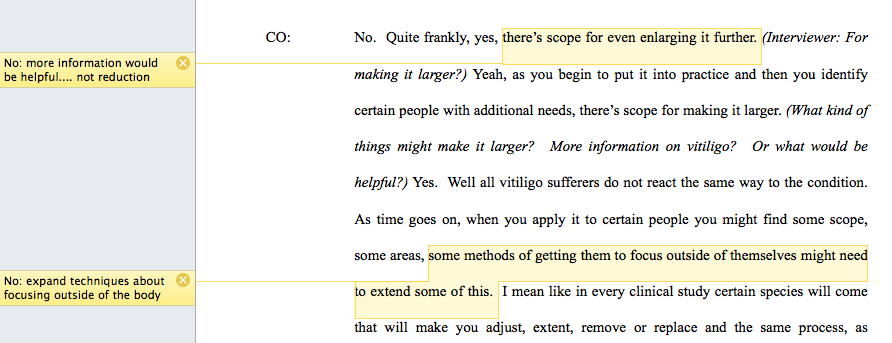


1.
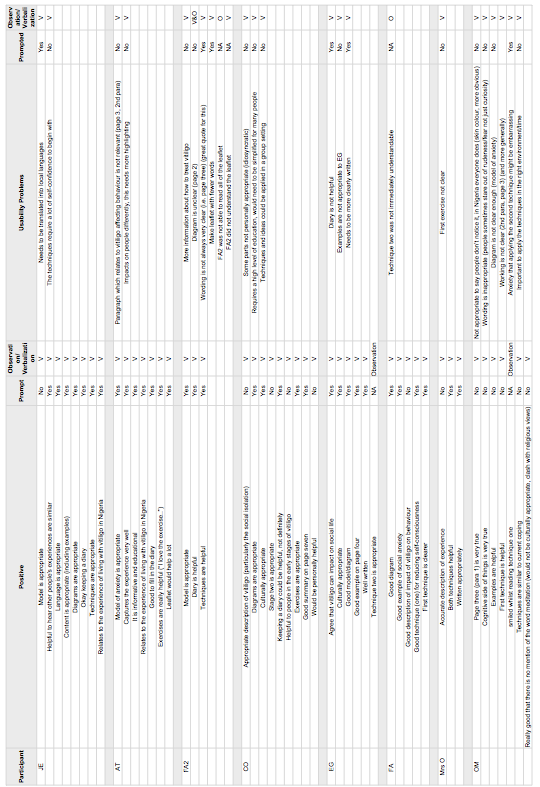
TA spreadsheet used to facilitate analysis
2. Eight-point guideline for conducting research in developing countries as outlined by Emanuel, Wendler, Killen and Grady (2003)


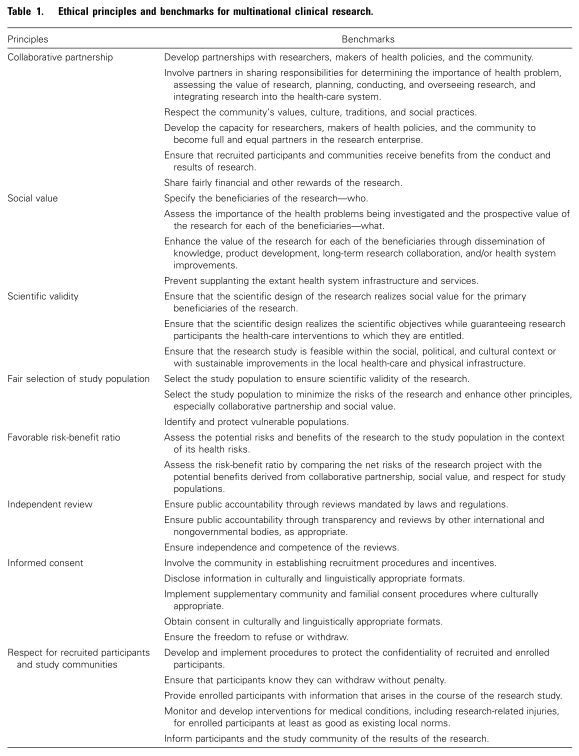

Supplement: sj-docx-1-hpq-10.1177_13591053241261684 – Supplemental material for The experience of living with vitiligo in Nigeria: A participatory Interpretative Phenomenological Analysis [file sj-docx-1-hpq-10.1177_13591053241261684.docx]
