# Supplementary material for: The experience of living with vitiligo in Nigeria: A participatory Interpretative Phenomenological Analysis
Source: J Health Psychol. 2024 Jul 30;30(5):1120–35. doi: 10.1177/13591053241261684 (PMC11977828; doi:10.1177/13591053241261684)
Supplement: sj-docx-2-hpq-10.1177_13591053241261684 – Supplemental material for The experience of living with vitiligo in Nigeria: A participatory Interpretative Phenomenological Analysis [file sj-docx-2-hpq-10.1177_13591053241261684.docx]

Data Set

Vitiligo Study

**Participant 1- AT**

Respondent: I first started noticing the change towards the ending of 2010. Yeah, 2010 I started noticing it from my palm then alongside with my foot before you know, generates to the skin. So ever since then I’ve been worried, what can this be, and I’ve not really seen somebody who can explain it to me that, oh this is what it is, not until I met somebody in December, Christmas, that was 2012, then the person introduced me to Mrs Ogo, so.

Interviewer: Right, okay, but when it first started happening...

Respondent: When it first started happening I’ve applied a lot of things, there were people with different opinions, oh this is what it can be, this is what I think you can use, and all that, so I’ve been using that not until 2012 when I met somebody at a Christmas party and the person just noticed it and said it’s like, “I know of somebody who has this kind of thing who can help you out,” and I know the person just in December 2012. Yeah.

Interviewer: When you first started noticing it you felt quite worried, did you?

Respondent: I got worried and things keep generating and a lot of people have been seeing it, “What is it? What is it?” But even myself I didn’t get the name that it’s been called, but I just noticed it in my hand and my palm so I didn’t even know what it is. And I thought it was the changing of skin and not until someone said, “Oh, this is so and so,” so you need to go so and so person that can put you right and tell you what you need to do.

Interviewer: So there was that early period of thinking who do I talk to who knows about this?

Respondent: Yeah.

Interviewer: And then you met Ogo.

Respondent: I met Ms Ogo. Yeah.

Interviewer: And who did you tell initially? Did you speak to your parents or friends?

Respondent: Yeah, I speak to my mum and she got worried, what kind of thing is this, and we pray that this thing does not keep spreading, and I even met a woman who discovered me that one of our brothers has such a thing, and it’s just changing of skin and nothing anybody can do to it. I even met my doctor and he said the same thing, that with time it will disappear, but I got myself worked up, what can this be? This thing keeps increasing, suppose it’s something that you have seen it change, but this one does not change, it keeps increasing, I keep seeing it on my legs. So I got worried but I just go to my mum and we prayed and all that and not until I met Mrs Ogo then things... And I believe with what you are discovering it and I know that there will surely be a change.

Interviewer: And what do you call it now? Do you call it vitiligo or do you...?

Respondent: It’s called vitiligo. Now I know the name, I know what can cause it and if the background’s done something or something there is pigment in our body that usually develops vitiligo, so I know what it is now.

Interviewer: How does that change your relationship with it knowing what it is?

Respondent: Doesn’t really change my relationship because she has given this kind of hope that this vitiligo is something that can be cured, it’s a matter of time, but you applying the right thing, the right dosage, then it will surely go in a matter of time. It gave me hope that surely the vitiligo would go.

Interviewer: So for you that’s an important thing is that hope that in due course, at some point it’s going to go?

Respondent: The hope. At some point it’s going to go.

Interviewer: Do you know when or do you kind of think just in your lifetime it will go?

Respondent: I believe in a short while it will go. But I have that belief that in a short while it will go.

Interviewer: That’s a powerful belief to have.

Respondent: Yeah.

Interviewer: I’m wondering what impact that has on you, to walk around knowing in a short while it will go, how does that change your having vitiligo?

Respondent: It doesn’t really change my thinking because there is something that I believe that once you know the right person to meet, when you meet the right person then whatever you’re going through, at least you have somebody who has trained on that same path before so she’s in best position to put you right. So I applied this to my own and it works for me so why can’t she just try the same thing? It’s not the same format but one way or the other there will be changes, so that has really given me a lot of belief and hope that it should go. I believe so it should go.

Interviewer: And in terms of your daily life, what impact does vitiligo have on your daily life?

Respondent: It does really affect my work because I am a cinematographer and I love to put on the shorts, T-shirts and all that to make me look more smart, (inaudible 00:06:09) jean, you know, first I will put on a jean so that you can look smart and you can work very well but now it’s given me a lot of concern, because I travel a lot, I work outside Lagos, I travel to east and all that, far place, so at times I will worry when I’m working in any location and somebody’s been disturbing me, “Oh, what is this in your hand? What have you been applying these things?” So at times I won’t be able to remove my socks or to free myself because I have to cover my legs in order to prevent a lot of questions and disturbance when I’m working, so it has really affected me in a lot of ways. But with time I know there will be changes.

Interviewer: For me that’s really interesting to hear, that you’re going out of Lagos to the farther areas of Nigeria, to more rural environments.

Respondent: Yeah.

Interviewer: And in those areas you’re more aware of your vitiligo.

Respondent: Of my vitiligo.

Interviewer: Why is that? What is it about those areas that makes it more...?

Respondent: One thing, I’ve noticed this kind of a thing when I was a small baby. When we are growing up we see some of these edali people with all these marks, but I’ve never had a thought of what it’s been called, whether it’s vitiligo or anything, I think maybe something’s been poured on them, maybe, what’s it called? Hot water and all that. So I never had this kind of experience in my life before, not until it happens to me now, so I can recall back then, when I was growing up I would see some of these edali(?) ladies, guys and all that with marks on their body, in their eyes, one side of their eyes and their mouth and all that. I’ve never for once believed

something that generates, that affects people, I never knew, not until this happens and I begin to get to know what it’s been called, what it was, it’s vitiligo, so.

Interviewer: So when you’re outside of Lagos you cover up more, do you?

Respondent: I cover up my hand because it affects most parts of my hand, and I have to lift my cameras and walk with my staff like that so they affect me so much, so I always put on T-shirt, most of the time you’ll see me with shirts now.

Interviewer: So you wear shirts now?

Respondent: I wear now a lot.

Interviewer: In Lagos is it easier?

Respondent: I would say it’s not easier because one thing there that’s been stopping me is a lot of people would stop me a lot and a lot of person who moves... I also walk with crowd, with my job, yu know? People will have to see you, there’s no way you could do it, you can’t cover yourself. You move in the midst of people, you talk to people, you meet people. And I also work in the ministry, I also work in the church here, so most of the time I’m with people and I have a lot of people under me that I work with, so a lot of them would stop me, “Boss, what is this one? What are you going to do with this thing? You don’t like this thing, you’ve got to work on it I know that.” So it’s really disturbed my work. It’s disturbed me a lot.

Interviewer: Has it disturbed your work so much that you don’t do work now or do you keep working?

Respondent: I keep working. It doesn’t stop me. It doesn’t stop me. But one thing that is I’m not too freer like before, because I don’t like putting on something that will shock me, I don’t like it, I love to free my body, to free myself, so... and it has changed my dressing a lot, it has changed my mode of dressing a lot.

Interviewer: Really? What were you wearing before?

Respondent: Before I loved to put on... if I’m putting on a nitsiv(?) now, on mostly Saturdays I put on a nitsiv Saturday, Sunday, Friday I put on a nitsiv. If I’m not on location I put on nitsiv. Like before I would put on a sandal but now I have to put on my shoe. I always go with my shoe.

Interviewer: And I imagine in the heat as well that’s quite difficult is it, to have to wear more?

Respondent: In heat it’s quite difficult because it disturbs me, it affects me a lot. I must confess it’s affected me a lot. It affects my mode of dressing, my work, even meeting with people because there are places that I have to go on behalf of my boss. Now I won’t put on something then I will get out and the person I want to go and meet is just asking me, “What happened to your hand?” So it has really affected a lot of things, but that does not stop me from working, don’t stop my work, but people asking me questions, I don’t like this, I love to free myself, to be myself.

Interviewer: I’m wondering when you walk around the streets does it affect you?

Respondent: Walking around it doesn’t affect me because I believe every minds our own business on the whole but the closer I am to you you will ask me what is this, “What happened to you? Something for you hand.” So…

Interviewer: So it gets to a point when you're getting to know someone that you know eventually they’re going to ask you?

Respondent: Eventually then they will ask you questions.

Interviewer: How do you manage that?

Respondent: What happened, I have been able to tell people it’s something I believe it’s a matter of time, it will go. That’s been my message, that it will go. I know it will go. I have that belief, it’s going to go, it’s a matter of time, and I’ve been able to talk to people, it’s something I can always explain myself. I can’t explain it but it keeps coming, it keeps increasing and all that, so.

Interviewer: Do you mind me asking what will happen if it doesn’t go?

Respondent: It will go.

Interviewer: It will go? So for you it’s not even entertainable?

Respondent: Yeah. No. It will go. It’s a matter of time, it will go. I believe it will go.

Interviewer: Has it impacted on your relationship with your friends?

Respondent: No. Nothing happened.

Interviewer: Your friends don’t mind that?

Respondent: We play, I play normally, it doesn’t affect my relationship with anybody.

Interviewer: And with your family?

Respondent: At all, at all, it doesn’t affect my relationship with them.

Interviewer: So it’s not so much your relationship with people it’s affecting it’s more kind of how, when you’re going out, you’ve got to wear wrong clothes and –

Respondent: When I’m going out, yeah, when I’m in a social gathering and all that my friends asking me, but two years ago because I left my (inaudible = workplace) station three years ago so most of my friends that we met together then, when we see now they will ask, “What happened to you now? This thing’s not there when we out of school so what is this? What happened? What happened to your head?” Most people that met me they were maybe three years ago now, once you see me now the first thing you notice is the mark. They will ask, “What is this? What happened to you?”

Interviewer: Is that the first thing they notice or is that the first thing that you worry that they’re going to notice?

Respondent: No, that’s the first thing they will notice. Because I have a lot of friends, I grew up in the midst of people, different people, so once we come across each other the first thing you notice is this mark, and they will ask, “What happened to you? What happened to you?” So it makes me feel bad a lot, it’s really disturbing.

Interviewer: So it makes you feel bad, it gets you down, does it?

Respondent: Yeah. It makes me feel bad. I felt, what is this? Because it keeps attaching more questions which I cannot even me myself I cannot ask, I will just be looking that it’s a matter of time it will

go, it’s a matter of time it will go.

Interviewer: You said it makes you ask questions that you can’t ask.

Respondent: Yeah. People ask me a lot of questions, and I just felt, what am I going to say to them? I wouldn't know what to say, because I cannot explain, I cannot explain what happened, so all I will say is, “It’s a matter of time, it will go.”

Interviewer: Do you find the questions quite tiring, people constantly asking?

Respondent: Yeah. It keeps coming and... (sighs) it’s a matter of time (laughing) it will go. It will go.

Interviewer: For you it seems for me hearing you talk that the most important thing is it’s a matter of time.

Respondent: It’s a matter of time, it will go. It will go.

Interviewer: And then that keeps you going.

Respondent: I believe so. It will go. I believe it will go.

Interviewer: On a daily basis how do you manage vitiligo? How do you cope with people’s responses, how do you cope with people’s questions?

Respondent: Like I said earlier on, I’ve not found any answer to it, but now that God has made me in contact with somebody which I know she has a better idea than what I did before, now it’s a thing I can call her anytime, any day now, “Oh, hello, Mrs Ogo, this and this and this, what am I going to do? What can I do?” So I believe for every problem once there is a solution then you can move up, so I believe there is a solution now to what I’ve been passing through. So I will be able to tell people what it is now, to explain to them what it is now, to explain to them what to do and to tell them that this thing, it’s something you can live with and it will go. That’s one thing.

Interviewer: You mentioned that God helped you find the right support.

Respondent: Yeah. Because I was saying it’s God because she gave me some of the pamphlets, question and answer, and I looked at it, I read through, that’s only about me, I read a lot, I love to just read, so I got through the question and answer and some booklet and I saw some people with a lot of... seen them with different skin and all that. So I believe, for God to help me at this stage I believed something that God can still do, because God has helped me to be in contact with her, to link me with her because he know it’s something that I’ve been worried because I’ve been praying, because I’ve prayed a lot and a lot, for him he can just help me out because I don’t like this, because it’s disturbing me, so I don’t want it. And God has really helped me to be in contact with her so I believe for every problem there is a solution and I think it’s time that God just wants to take it out of my body, so I believe.

Interviewer: So spirituality is something that helps you manage?

Respondent: Yeah.

Interviewer: And does your pastor and your church, they are supportive of you?

Respondent: My pastor in the church, my boss now, he’s a pastor, so he’s aware of what is going on, he is aware. I even met a doctor, a female doctor in the last month or so and she asked me to come that she travelled outside the country to get some drugs and she promised me get me

some of these drugs that can work with skin, you get. So during that period I went through and during that period it was Christmas period or so, so I travelled out of Lagos for almost three weeks or so and so we lost contact and all that, so if... (inaudible 00:19:00) after I’d met with the woman to just get the stuff from her, so I travelled, coming back to Lagos and someone just told me that (inaudible 00:19:12) travelled out of the country back. So I couldn’t get in contact with that, so between that period this is when I get in contact with somebody who knows Mrs Ogo, so.

Interviewer: So you were kind of alluding there to physical treatments, to somebody bringing drug treatments into the country. Have you tried any physical treatment?

Respondent: I have not. I have not tried anything.

Interviewer: I think in the UK something that’s quite popular is kind of make up, people wear kind of cover up which changes the colour to match the colour of their natural skin. So is that something that you’ve explored or do you use that?

Respondent: I won’t pray that I will use that. I am not even ready to use that. But one thing I believe that my natural skin will come back to life. That is one I believe. I believe that God can do it, that can bring my natural skin back to normal. I believe so, so I don’t have any doubts, it will go, so my normal skin will come back. I believe that. I believe so, so it’s a matter of time.

Interviewer: And that’s the driver for you? That’s the big thing to take away?

Respondent: That’s the drive. Yeah. Because I keep saying it, it’s what you keep saying that will happen to you, you command and you keep ensuring(?) it, so it will come. It’s a matter of time. Once you believe and you are patient with it, so it will change.

Interviewer: So would you try any treatment or would you explore treatments or would you rather just...?

Respondent: Yeah. No, I will try treatment. It’s a natural thing that can happen to anybody so I will try treatment, at the same time I will believe on the word of God and I believe it will go.

Interviewer: So God is incredibly important for you in this?

Respondent: Yeah. It’s God first. The treatments, it’s something that you just need to use, to obey.

Interviewer: Because the treatment will come from God?

Respondent: Yeah. Come from God, but I believe that God will do it. Something God can do. So I will believe, I will stick my mind to God, pray to God that bring back my normal skin, at the same time I will go with the treatment. So far as it will not have any effect on me, damaging my skin, I will use it. That is it.

Interviewer: One of the things I wanted to explore earlier and I forgot to ask you when we were talking about what impact it has on you, does it affect your kind of romantic relationships at all?

Respondent: Not at all, it doesn’t affect them, no.

Interviewer: So it has no impact at all on...? No.

Respondent: Not at all.

Interviewer: How do other people react to vitiligo? I guess that’s one of the things I’m trying to understand. So for you how do other people react? Are they scared of it? Are they worried by it? Are they curious, are they mean?

Respondent: One thing that I will say is I have not seen somebody who has this kind of thing before. But like I said earlier on, when we were growing up I’ve been seeing it but I don’t know what it is, I don’t know it, I don’t know the name, I don’t know what causes it. I thought it was maybe something poured on them and treat it like a fire accident and all that, so that is my own belief then. But now that it’s happened to me now I know what it is, I know what they can do, and I know who to go and meet that can direct me.

Interviewer: So that makes it much easier?

Respondent: It makes it more easier.

Interviewer: So I guess really what that is is it’s a support network isn’t it? It’s having people who can support you who have been there before.

Respondent: Yeah, who have been there before. They can put me right, put me on the right track.

Interviewer: And that’s incredibly helpful. So it’s having the support network, it’s having God, and it’s having the belief and the knowledge it’s going to change?

Respondent: The belief that it will go, yeah, that it’s going to change.

Interviewer: So they’re the very important things.

Respondent: Those are very important things.

Interviewer: Thank you so much for sharing all that with me.

Respondent: You’re welcome.

Interviewer: I think probably a really important question is just to ask you have I missed anything? Is there anything that you think, “Ah, Nick should have asked me that and he didn’t?”

Respondent: Nothing.

Interviewer: Nothing at all?

Third party: You should think, think, think, think. It’s important. Just anything you want to share, maybe an experience you had or something.

Respondent: What can it be? Nothing. One thing there is, I believe there are a lot of people who still have this vitiligo there are a lot of people somewhere, somewhere who needs help. I want it to be something that maybe you have a TV station, you have a programme for it, because I believe it’s something that a lot of people have within them, they have in their body, so they don’t have somebody to share it with. Because if you know the right thing to do then you know the right channel to follow so that the problem can be solved, but a lot of people have this thing. Even in the... what’s it called? Outside Lagos a lot of people are living with this thing but it’s killing them, they can’t share it with anybody, so I would want you to do maybe a TV programme, a programme that will shed more light to this thing.

Interviewer: So to spread the word?

Respondent: Yeah. To spread the word, to make more awareness, because a lot of people are living with it, a lot.

Interviewer: So for you, you imagine there’s a lot of people out there who have had the same thing and they’ll be going through the same experience?

Respondent: Yeah.

Interviewer: And because you’ve found the support network so helpful and for them it might also be.

Respondent: Yeah. That is it. Something to make more awareness, make awareness, because it’s like HIV, a lot of people have HIV but they don’t know, they don’t know anything because not until there is awareness, oh, so somebody has this thing somewhere, somebody have this thing somewhere. So we need to make more awareness.

Interviewer: I guess one of the things I feel maybe I haven't asked you enough about is the impact on your mood and on your feelings.

Respondent: It affects my moods, it affects my feelings, but I don’t allow it. I don’t. I don’t allow it to control me. It’s something that disturbs me but I don’t allow it to stay in my mind, I just play over it, take it out of my mind and (inaudible 00:26:10).

Interviewer: What’s it trying to do? What are you having to say, no I won’t let that happen? It makes you feel kind of depressed and then you go, “I’m not going to do that?”

Respondent: It makes me feel depressed at times, makes me feel kind of depressed. Once I woke up in the morning, first thing I see is the mark, I will look at myself. This thing is still there, Lord help me. It depressed me a lot but I don’t allow it to control me.

Interviewer: So for you it’s important that you’re in control of it and it’s not in control of you?

Respondent: I am in control of it.

Third party: I wanted to ask, did you at any point feel that a ritual is a cause, it’s something that somebody did to you, or did you ever get that kind of impression from someone?

Respondent: I never had that impression. (Laughing) I never had that come (inaudible 00:26:55).

Third party: And nobody ever suggested that?

Respondent: But one thing that is, you know the female as my... I’m talking about my mother now, because I called her...

Respondent: So my mum, she felt something like a cause or like a background (inaudible 00:28:23) I immediately I change her perspective, I change her thinking, “No, this is natural, its cause, nobody, so don’t ever see it as a spiritual thing. It’s normal and one thing we should believe, it will go.” That’s my belief, and I believe so much.

Interviewer: But your mum was questioning was she?

Respondent: Yes, questioning maybe it’s spiritual stuff, you know.

Interviewer: So your mum thought maybe it’s a bad spirit?

Respondent: It’s a bad spirit and all that so. So I change her mind, I change her perspective on it.

Interviewer: And did she believe you?

Respondent: Yeah. Ever since then we’ve never... but all she asks now, “How is it going? I hope it’s getting better.” “Oh, it’s okay, I’m okay.” So…

Interviewer: Thank you very much.

Respondent: You’re welcome.

Interviewer: I’m going to stop the recorder just for a second.

[End of Transcript]

**Participant 2- CO**

Interviewer: Can you tell me when you first noticed the changes on your skin?

Respondent: Like I said it’s 1982. Prior to that I had a fever and I went to hospital and I had some injections but then after my lips dried, and when they pealed off they remained pink thereafter. Then like a year or two thereafter really the pink lips came like in 1980, I remember, that was when my family came in from the UK, my wife and my baby. Then I discovered the... you know, pink patches at the tips of my fingers. And then thereafter my palms became more pinkish than they were before and then it started creeping upwards from my fingers up to my elbows, attacking the back first. And then as the years went – well I went to the hospitals, I went to the local teaching hospital here where they gave me some funny injections. Back then vitiligo wasn’t popular, you know, it wasn’t commonplace like it is today. Or maybe I didn’t notice those who had them. And their efforts to try this and that, they gave me funny injections and things in Lagos here. Then when I went to UNTH the funny part of it was that in between the Lagos treatment and the soca(?) treatment my elder brother, I found out that my family members were... they couldn't face me to say, they thought what I had was contagious but I later realised that they were avoiding me in a way. And my elder brother actually took me to a leprosy clinic. I drove my car down here from here to the east and then we went to Zapoly(?) leper colony. It was then I realised that they actually thought what I had was contagious.

Interviewer: Was that quite soon after you noticed it?

Respondent: Yeah, after a while, say about two years or thereabouts. And the doctor asked me, “Does it itch?” I said, “No.” “Do you have any pain?” “No.” “Do you have any numbness in the tips of your fingers?” I said, “No,” too. “People shouldn't have brought him here, there is nothing wrong with him, it’s pigmentation loss.”

Interviewer: And what did your brother say when the doctor said that?

Respondent: Well actually he’s the eldest, I think probably discussed with the younger ones behind me, he was the only one who could tell me to do such a thing because we have this order in the family. The eldest, the first born, has control after the father. Whatever he says you do. And all those behind me do what I say they should do, so they probably discussed and said, “Well, you're the only one who can tell him.” So. We go out there and the doctor looked at me and said, “No, there’s nothing wrong with this man, it’s just pigmentation loss.” Actually then I didn’t know what was wrong with me, and at that point most of the things you put in that questionnaire were correct. I was self-conscious, I was allergic to people looking at me. I could quarrel with anybody if you looked at me for long. I used to have a very nice face and then suddenly when that went, initially I was fighting against it. So I now had the second visit to the other teaching hospital where the doctor looked at what they had given me and said, “No, that is for keloid, it’s not for that.” He now recommended I should come back to his clinic and he has some things to use topically and asked me to avoid bleaching creams and all that. Before then I wasn’t used to using any sort of cream at all, I was ebony black so I didn’t need any cream. My body was oily so it wasn’t dry so there was no need for me to use any cream at all before then. So I tried some of the soralim(?) paint and all that and all that. I am a free spirit, you can’t cage me, you can’t give me any type of routine that I must do every day. I can’t.

Interviewer: So you didn’t want to do –

Respondent: I just stopped them, until the time I met her (Ogo) about four, five years ago, and it took her three years to convince me to come to the vitiligo thing, so it’s in the last two years that I’ve interacted with them. My life is peculiar in such a way that since it was there there was no

pain, I just looked at it as a new face.

Interviewer: Right, so when it first started emerging that was your kind of thought?

Respondent: Yeah. I reacted negatively towards it. I was angry and all that. But my wife accepted it and my children would even boast of my pink lips and all that, so who am I, so I reconciled within myself. So whatever you do or say, I would go to the market, women would not collect money from me and I would drag them and say, “You have to take your money.” (Laughing) Then I now found out that people paid a lot of attention to me when I talk, because of what I look like, and in the Christian group I belong to, Charismatics in the Catholic Church, I do a lot of teaching so I found out that nobody would stop paying attention to me as long as I talked, as I was there talking. And because this was a peculiar person, confident and talking without being ashamed so it took so many people out of their own misery, so rather than being a negative weapon against me it’s not something that actually works on people.

Interviewer: So people are drawn to it?

Respondent: Yeah, people are drawn to me. They say, “Ah, is it a fire or something?” I said, “No, it’s natural.” Even now people look from their buses at me in my car and I look at them and I smile at them. They are surprised, you know?

Interviewer: Is that a different response to when you first –

Respondent: Oh yeah. When I first came, I mean I could have committed suicide because of it if I had the chance, but if not for Christianity I say why should I change? So after asking many questions and taking many medications, and rather than recede it was going forward and spread. I let it

go. So it’s my identity.

Interviewer: So was that a conscious decision to change from you fighting it to accepting it as your new identity?

Respondent: Yes. Because there is no way of fighting an invisible attacker. This thing is invisible, the source is invisible, I see it spreading. And from time to time it recedes, some areas will darken on their own, and then it comes back full blown and then it extends its territory. I say, “Why am I worrying myself?” I reconciled with it that I have to keep this face and this body the way it is. So the only drawback is that in the intense sun I quickly get sunburnt. If I stand in the sun very hot for three hours my skin will peel off.

Interviewer: So that’s a big difference?

Respondent: Yes, that’s a big difference. That is the only effect of vitiligo on me. Psychologically it made me a stronger person but no, so logically I have to avoid the sun and very hot places because I will darken and the next thing I will peel off. So that’s that.

Interviewer: I’m wondering when you first started noticing it who did you tell? You told your family?

Respondent: No, my wife was there, all of them were, they were noticing it at the same time with me.

Interviewer: So you were already married at time?

Respondent: Oh yeah, sure.

Interviewer: And were your children born then?

Respondent: No, only one.

Interviewer: And how old was that child then?

Respondent: She was born in 1978.

Interviewer: So she was four years.

Respondent: Four years, yes.

Interviewer: So your children, they’ve grown up with it.

Respondent: The others came and saw me that way and they've seen it spreading. By the time the next one came in 1982 I already had it, very tiny on my fingertips and only my lips, there was none on my neck or anywhere. By the time the next one came in 1986 it was spreading but not this much. Then in 1988 when the last one came it was still not moving that fast but it was up to here, you know, it’s gone from there, it’s going towards my elbow but it stopped there, didn’t make much progress. But I think in the last ten years it’s really done a marathon, it’s expanded like wildfire and I’ve just left it alone. Yeah.

Interviewer: When you told people originally in your family you mentioned that they wondered about leprosy.

Respondent: Oh yeah.

Interviewer: But also how did they react to you?

Respondent: Well in that time because I’m such a strong character I really didn’t notice people drawing away. I hadn’t much time to dwell on anybody. I mean that’s my nature, I was too active to look back and see how people are reacting and what they’re doing after I have gone past them. They would come to my house, we would play and chat, but I had that nature of having a particular seat in my house, and if I come to your house often you’ll find that I’ll pick a particular seat, and I’ll sit in a place that I observe everybody else, I will cast a glance on you, and go, “I’ve made up my mind what is in your mind and what you are going to do next so it won’t surprise me,” so I just sit down there. So I wasn’t aware that there was this feeling within my family until I now went to the leprosy clinic and when I came back I now told them, I said, “Look, there is nothing wrong with me. What is wrong with me is my nature is changing, my skin is changing, and it’s not a sickness, so you could relax,” and they just took it that way.

Interviewer: But you mentioned that there was a time early on when you felt suicidal.

Respondent: Oh yeah, because I felt my face mutilated. I mean I used to be very proud of what I looked like and now with all these patches all over the place you can’t really say what I look like anymore and all these things, so I sort of stopped... I would not look at my face in the mirror.

Interviewer: So did you avoid them?

Respondent: I’d avoid the mirror, yeah, I will avoid the mirror. I will comb my hair without looking at the mirror. I will put on my clothes without looking at the mirror. I would not look at my face for quite a long time.

Interviewer: And during that time were you feeling very depressed?

Respondent: Yeah. I was depressed. But it didn’t stop me because my work, I had to work, so, I had a lot of work to do so I didn’t quite have free times to dwell on myself. And plus at that time the economy here and the construction industry where I work was going down.

Interviewer: So you were working in the construction industry then?

Respondent: Yeah. I still do. I’m into engineering. And I was working for a company that was suffering, you know, very few jobs so most of my colleagues were laid off, I was the only one running everything, so I was busy from morning until night, so only the time maybe casually I see myself in the mirror that I remember, “Oh, this is what I look like now,” so I had the cloak of work and being busy covering what I looked like and my spare time which actually would allow me to look at myself.

Interviewer: So did you therefore immerse yourself into work?

Respondent: Yes I did, and I kept away from social gatherings as much as possible.

Interviewer: Do you call it vitiligo, is that the terminology you use?

Respondent: Yeah. That’s what we call it here. Well that’s the only name we know it by. On the internet and other place they call it white leprosy whatever, whatever.

Third party: No I think he meant the first time.

Respondent: The first time, no I didn’t know what the name was. It was much later than I knew what the name was. Not even medical doctors didn’t know what the name was, way back in 1982. They didn’t.

Interviewer: So it was back then that the doctors seemed quite...?

Respondent: Oh yeah. They were just mucking about trying to find out what was similar to it. It was only at Inugla(?) they took part of the skin. He told me to come back for a skin graph and the sort of money he was asking for was too much for me. The children had to go to school, in an economy that was depressed so I couldn't go back.

Interviewer: Have you continued to engage in the health services since that time? I’m wondering whether you've seen any change.

Respondent: What I have observed is that it comes at a time, I darken a lot and then it comes back. Between you and me I am into the vitiligo thing now to create awareness for people who are suicidal, people who are depressed because of it. I want to be a source of inspiration to them. I am not doing anything to change this. I wouldn't really want to go back to being completely black.

Interviewer: Because now it’s your identity?

Respondent: Oh yes. I wouldn't want to go back.

Interviewer: So I’m wondering how it does impact on your life now?

Respondent: Well, like the bible says, somebody is the son of encouragement, Barnabas is it? When you come and you’re depressed and we meet anywhere, you are depressed about anything on your skin or any suffering and I’ll draw you close and I’ll talk to you. You’ll relax and you’ll begin to see things differently. So that is why I wouldn't want it to go back to that.

Interviewer: Because it’s allowed you to see things differently?

Respondent: It allows me to serve other people and that’s fulfilling.

Interviewer: Do you find in your thoughts and feelings it’s had an impact today? Because I know you’ve spoken about the past, but today you feel it allows you almost a freedom does it?

Respondent: Yes, it’s a positive thing today but initially it was negative. It was destructive then. Maybe out of the touch of God and maturity and seeing other people I realise there are people that need help, so you know, I don’t call it anything anymore.

Interviewer: How about in your work, does it impact on your work life, now?

Respondent: Initially I’m something like something of interest, like a new movie to people when we meet the first time, but as time goes on and when they begin to see that there is much more behind this mask, they relax. People usually are tense when I come into their midst, particularly those who have an even complexion and have not had much contact with people like me they are sort of far away. Well, I am strange, aren’t I? In a sort of way, I am strange, so people do not... people who are vain, let me use that word, it is only vain people. I realise that those who are vain who attach more importance to the outlook, what they see on the surface than the substance of the being that they are with, will always stay far away. Okay? But in the

process, maybe we’re in a meeting, maybe we’re discussing a project, they generally realise that what they’re looking at is a normal human being and in some cases very, very articulate in what I do and say, when they know it, they relax. And at the end of the day they say, “What happened to you?” and I’ll tell them, “It’s natural, it’s vitiligo, it’s a loss of pigmentation, and it can happen to anybody.” And after that first meeting they generally begin to appreciate, maybe they’ve been drawing back from other people like me with these patches, now they will go closer and ask.

Interviewer: So you feel that there’s almost a process, that when people first meet you they stand off a little bit?

Respondent: Yeah, because the don’t know what you are carrying. They are not educated about it.

Interviewer: So you think it’s fear from them?

Respondent: Yes. But as time goes on and you explain to them, little children will see me with their mother and they say, “Mummy, look at that man?” You know, I will stop and go and grab them and I will rub my fingers on them and they see there is no stain and that’s the end of it, so those children will never again react with amazement towards someone who looks like me. That’s what I’ve been doing.

Interviewer: So it’s very important for you is it to play a role now?

Respondent: Oh yes.

Interviewer: To represent for people with vitiligo?

Respondent: With vitiligo, yes, it is.

Interviewer: I’m wondering over the years how your family has changed in its view towards... you said that your children never noticed and were proud of it.

Respondent: Oh yeah.

Interviewer: What about your brothers, has that changed and your parents and how’s that?

Respondent: Well at the time it started my mother was the only one around and she was worried about it. Even up to the time she died she was worried, “Why are you changing, changing.” I said, “Mamma, this is it.” And then somebody reminded her that her father-in-law was like this. She says, “Yeah, but has it got to be you?” I said, “Yes. That’s me.” I mean she was the only one who always complained about it. My siblings, none of them complained.

Interviewer: One of the things that Ogo’s(?) kind of brought to my awareness is the idea that in Nigeria there can be a view that it’s a bad spirit.

Respondent: Yes.

Interviewer: Kind of evil spirits. Has that ever been something for you?

Respondent: Yes. Some people say something that I did that is wrong. Some people say that I’ve done something against traditional custom. Some people even say that I’ve eaten something that I was not supposed to eat, and some people even say, “Don’t eat crayfish, don’t eat this,” and all that. They all attach it to some form of spiritual thing. Well if it is spiritual then it is God’s

spirit, not any other spirit. In some places people will see you and they’ll run away. They’ll turn the other way and quietly move away.

Interviewer: Which places?

Respondent: Well I’ve gone to some villages recently where I saw some people turn around and go but you know what, they didn’t get too far. I went after them.

Interviewer: So is there a difference between being in Lagos and the responses you get in the city?

Respondent: And some remote places, yes. Some people have this fixed idea, attitude. They pretend to be Christians but they are still into traditional ways of things. Albinism is supposed to be a spiritual thing in some places due to their culture and their nature, but as they come out and mix up and see that there are many more people like that, you know, they sort of mellow down.

Interviewer: So it’s a process again. It’s like people initially react quite badly and then –

Respondent: Oh, like education.

Interviewer: It’s an education.

Respondent: People have to be educated, yeah. They have to be educated.

Interviewer: You've told me about how it impacts and has in the past impacted on your life. I’m wondering how do you cope with it? What are your day-to-day coping strategies, like how do you think you get through?

Respondent: Nick, it’s like putting on a shirt. This thing is a shirt I’m putting on. All I have to do is wash the shirt and iron it, make sure it’s clean, it’s simple. I don’t need any coping mechanism.

Interviewer: Because now it is who you are?

Respondent: It’s natural. My skin is who I am. It’s who I am.

Interviewer: But do you think that you manage other’s responses in a different way to how you used to?

Respondent: Yes. It makes me look at people who are not exactly how they should look with compassion. I would rather come closer to someone who is deformed than keep far, you understand? So it brought us some sort of kindness so to say, feeling of kindness towards people.

Interviewer: So a greater kind of level of compassion within you towards others?

Respondent: Yeah, within me towards others. So I think it’s made me nobler than I should have been if I was prim and dark and handsome. So I look at people now who have blemishes, deformities, with a lot of kindness and respect and I would like to get closer to them to help them to bear whatever it is, stigma, rejection or whatever.

Interviewer: One of the things you mentioned on a number of occasions is the role of God or the role of religion or the church, is that very important for you in the way that you cope as well or the way you function?

Respondent: Yeah. I mean if I hadn’t God’s grace I don’t think I would have coped. I don't think I would have coped, because I’m a very intense human being and any form of rejection could trigger some internal and external reactions, so it has made me appreciate that whatever you say or do there is a force behind our existence and he determines what goes. Maybe one day I will turn back to dark, maybe I’ll have an even complexion that is between dark and vitiligo, but I’ll leave it to time, and I’ll leave it to the research you people are carrying out, but I am okay as I am.

Interviewer: Has the church always been a place that has been supportive of you?

Respondent: Oh yes. I am of the Catholic Church stock, it’s not a small place. They have to be useful in one aspect or the other for you to find a foothold, otherwise you go there on Sundays and pray and go home. But like I said, with the charismatic renewal movement into which I am we do a lot of things, we pray a lot and we work hard a lot, there are daily exercises that won’t even let you notice what you are and what you look like or whatever, and it makes you get a deeper grasp of this force, this being, this author that can create people like you with even complexion and also create people like us with different shades all over the body. I mean even between... even now in the skin you notice that some areas are not... okay, now, this is a sign of repigmentation now, it’s spreading, it will get darker like these ones, and before you know it this will come very close to being dark, then it will go. Somebody is behind this. Some authority is behind this and I acknowledge that he is God, and as long as I acknowledge that he is God I have no problems.

Interviewer: I imagine though that you’ve had many years of living with vitiligo.

Respondent: Oh yeah.

Interviewer: Have you tried many treatment options during that period?

Respondent: No. As the years went by, as I moved from the second year to the third, to the fourth, and the fifth, to the tenth year, I sort of tapered down, you know, the scale went down, treatment, I stopped asking for any treatment.

Interviewer: So as the years went on you looked for less and less?

Respondent: Yeah.

Interviewer: Why do you think that was?

Respondent: Because I had reconciled myself with what I was carrying. All the medication and everything rather gave me reactions and sickness and I became weak sometimes and I became sort of restless sometimes with certain medications and each one that gave me a reaction I dropped it. And it got to a point I said, “Wait a minute. Why am I wasting my resources on medication?” So the things that I try now, the things that don’t cost me money like duguyero(?) and all those things that I chew the leaves and... that’s it.

Interviewer: And do you do those specifically for vitiligo?

Respondent: Oh yeah.

Interviewer: And have you noticed any impact?

Respondent: I mean like I say, sometimes the pigment’s back and sometimes it goes, it recedes, so I take them to keep the balance, and hopefully any change in me would mean that there is a possibility for other people to change. So we are jointly taking the treatments to see which one works so that others can do that.

Interviewer: So kind of exploring natural options.

Respondent: Yeah. As a voluntary guinea pig, yes, I will take anything that is recommended and if it works on me then other people will have a source of, you know, cure. And those who will have gone made because of it, those who will have dropped out of school, like I’m sorry for the younger ones like when I met there in the last meeting, I mean by the time she left us going back to school she was more human. And I keep calling her on the phone now, she is in the secondary school, and I encourage them to stay focused, that the top of the skin has nothing to do with the brain matter and the heart and the future ahead.

Interviewer: I think it’s nice the way you put that. So it’s nothing to do with the brain or the heart.

Respondent: Yes. That’s the way it is.

Interviewer: For you it’s just the skin and those two things –

Respondent: Yeah. It’s a cover. The shirt, it’s not the man who is putting on the shirt.

Interviewer: And you talk about the meetings that you attend, can you tell me a little bit more about those?

Respondent: Well we have our usual meeting, the vitiligo meetings from time to time, because we invite people. She will tell you more about that, she’s the organiser. So a lot of people come in like the last one we had with the professor from India and the rest of them, there were school children, there were married women who had been left by their husbands and when these people meet people like us and we sit down and talk with them and make jokes of the skin pigmentation. And generally realise that people who are worse off than them, you know, physically looking, but who are confident and positive, so it impacts on them and they go back and they are now able to fight the stigmatisation and the rejection in the society.

Interviewer: So is that purpose for you quite important in your identity now with vitiligo?

Respondent: It is. Yes. It is, very important. Because it saves souls from depression and madness and despondency.

Interviewer: So it’s building a support network.

Respondent: Yes.

Interviewer: It sounds to me like you have a number of support networks, that you have the church, you have the –

Respondent: Yeah, really in the church I am rather one man support group there because what I say and do helps people who are not into the vitiligo scheme for instance, and people who are stigmatised for one reason or another, the deformity or speech or anything. I tend to let them know that their conditions are known to God in the first place, and if they accept to be children of God they have to live with those things. Some of them will take as crosses, I take my vitiligo as a cross, I take it as an attack from the evil one knowing that I was proud to an extent and I liked what I looked like and I mean I couldn't hide, and then it turns me the other way, obviously an

attack to make me... well let’s see what you will be proud of. And that inner strength, you have to give it to people who are hurting one way or the other because they do not look like others or because they have failed in one place or the other. So that’s what I use it for in the church. So it’s not the church now, well the church gave me the initial stability to pray, to think positively in any situation and with further training in spiritual matters I stabilised so to say, and then you have to give that stability to other people. You do not need a group now. Well, wherever you find yourself in the Charismatic Renewal you know that you have a function to perform and you have to perform it as a function.

Interviewer: Thank you. The two things that came out from you there. One was you used the word stigmatised.

Respondent: Yes.

Interviewer: I’m wondering, is that how you have felt? You've felt stigmatised over the years, have you?

Respondent: Oh yes, initially, because you go to the market, certain people who have little or no education and who have not witnessed you, they tend to equate you to a situation of a leper, so when you get money out of your purse somebody doesn’t want to collect the money from you.

Interviewer: So there’s a fear?

Respondent: Of even contamination. And that has passed, because people are now with us preaching and talking on the television. People can now recognise a leprosy patient. The skin peeling has form of rot(?) and then if it is healed it’s stunted. It’s not like ours that it’s completely has no... they can now see the difference because people like us are able to come up and talk to people and tell them the difference. And now it’s not like that, people do not generally wherever I go

into the open market or whatever people are now able to differentiate between the hand of a leper from the colony and the vitiligo patient’s hands, so that’s an achievement, yeah.

Interviewer: The other thing I wanted to just ask you about there was you mentioned that you felt that it was a kind of... somehow a punishment from the evil one because you had been proud of how you looked. Is that still how you feel?

Respondent: Not a punishment. You see, if you are a Christian like me you realise that the evil one cannot do anything to you unless God allows it, so if you keep flogging the evil one saying he is doing that, the evil one is doing that, he has to get permission from my father in heaven. And if my father in heaven permits it the evil one is of no consequence anymore as long as I accept it as coming from God, he is finished. His own is finished. And then if I accept it as something allowed me from God then there is a strength in it.

Interviewer: So you've taken strength from that?

Respondent: There must be something positive coming out of everything that comes from the almighty God. It looks negative but when you explore it there are certain strengths that you couldn't or you wouldn't have been able to achieve if you had not accepted that situation or condition initially.

Interviewer: So it’s about seeing the positive of it and seeing how it’s helped you develop as a person?

Respondent: Oh yes. There must be something in it that will help you that will help humanity.

Interviewer: It’s about finding the good?

Respondent: Yes.

Interviewer: Just too more questions if that’s okay, one is just how do other people react to your vitiligo?

Respondent: I don't know. Because (inaudible 00:34:14) positively I don't know. I don’t stop to find out how they react. Because I use it, you see my own is attack rather than defence, so I don’t give you time to pity me. I don’t give you time to react in such a way that it affects me. I will come to you and I’ll become your friend, and whatever reaction you had conceived is buried because I will not allow you to table it. You understand the way I operate? Alright.

Interviewer: Yeah. Thank you. My final question really in this section is just, I’m aware I’ve had a list of things and I’ve roughly followed that schedule of questions. I would like to really ask you whether you think there’s anything that I’ve missed that you think I should have asked.

Respondent: Well, if there is there will be another opportunity you can email me. If there’s anything else you need for this research you can ask me or you can ask Olga.

Interviewer: That’s kind.

Respondent: Well I know you didn’t ask me about family, my family.

Interviewer: Perhaps we can talk about your family.

Respondent: I am the second son in a family of 14. My father was a polygamist, he married three wives. Had 16 rather, 16 children. Yeah. And my elder brother was the first child in the whole family, aged 73 or thereabout. He’s right now doing a PhD in America. He retired from teaching in the (inaudible 00:35:57) seminary here and went to America to do his PhD. I have seven other brothers and nine sisters. We’re all alive. Okay? Well as I married, I was married until April when my wife died, last year, 15th of April, so almost nine months now. I have four children, two boys and two girls. The eldest is a girl, she’s based in England, she lives and works in England but she was born in London and she went back there. The rest were born in Nigeria, they are Nigerians so they are here. The youngest is 26. A girl. It started with a girl and the two boys. They’ve all left university now so maybe I’ll go back to school too.

Interviewer: Maybe you’ll go back?

Respondent: Yeah.

Interviewer: And that’s a large family to come from, a lot of people to think there.

Respondent: Yes. And I happen to be the hub of this family, the centre of gravity of this family. Everybody relates to me, all relate to me. So you can see why I have to be stable.

Interviewer: So you have a responsibility.

Respondent: I have a responsibility to give confidence to other people. I don’t have time to look at myself and wallow in self pity, I can’t because it will affect a lot of people.

Interviewer: So you have to stand up, you are the person that people rely on.

Respondent: Yes.

Interviewer: And has that helped you to be able to be that person?

Respondent: Yes, because you realise you have a responsibility to achieve. You have authority that you get as a responsibility. If you do not do it then things will be bad for everybody. So their hub, the centre of the world, there is no break up otherwise the tyres will fly. So with that you don’t have time to worry about what we’re talking about about vitiligo, and it’s the same process that I use outside.

Interviewer: So it becomes less relevant to you?

Respondent: Oh yeah, it is.

Interviewer: Thank you very much, Chris. I think we’ll close that part now if that’s okay.

Respondent: Yeah. Okay.

[End of Transcript]

**Participant 3- EG**

Interviewer: Can you tell me please, when did you first notice the changes to your skin?

Respondent: That was when I was 21.

Interviewer: What happened, what did you notice?

Respondent: I just saw a white patch on my left hand. That was how it started.

Interviewer: What did you think when you saw the white patch?

Respondent: I thought it was maybe reaction.

Interviewer: A reaction to what?

Respondent: I don’t know. I don’t know if it’s a reaction to something I have been taking. What I did, I went to a man selling cosmetics, I showed him then he gave me a cream to apply there. As I was applying it wasn’t going, it was expanding, so.

Interviewer: So who was the man who gave you the cream?

Respondent: Just a cosmetic man.

Interviewer: In a shop?

Respondent: Yeah.

Interviewer: And he recommended the cream but it didn’t work?

Respondent: Yes. It didn’t work. I found him by the name of the clinic. (Inaudible 00:01:08).

Interviewer: Oh that doesn’t matter. I was wondering how were you feeling when you first saw it, what were your thoughts and feelings?

Respondent: I wasn’t happy, I felt very bad.

Interviewer: You felt very bad?

Respondent: Yes.

Interviewer: What do you mean when you say you felt very bad?

Respondent: Because it was disfiguring my skin.

Interviewer: So you felt disfigured by it?

Respondent: Yes.

Interviewer: So when you say you felt bad, were you feeling depressed?

Respondent: Yes. I did.

Interviewer: And you were feeling just sad about the situation?

Respondent: Yeah, I felt very bad and I tried using something and I went for some herbs. It wasn’t going before I went to the hospital to see a doctor.

Interviewer: So you tried herbs and the creams first and that didn’t work so then you went to see a doctor at the hospital?

Respondent: Yes.

Interviewer: What did they say?

Respondent: Said I had vitiligo, they called it vitiligo, and they gave me a cream called Maladin(?).

Interviewer: Did that help?

Respondent: It didn’t help, because the doctor said I should applying it, as I was applying it there it was coming up other places, so I went back to tell the doctor, he said I should keep applying, but anyway it comes up in any place I notice a new one coming up, I should just keep applying there and also stay under the sun for like 30 minutes before washing it off, and I was doing it but it wasn’t helping, the condition was getting worse and I was so worried and feeling very, very unhappy.

Interviewer: So you felt very unhappy and you were worried and I’m wondering who did you tell?

Respondent: My parents, my friends, everybody around me.

Interviewer: What did they say? How did they react?

Respondent: Well, initially people were saying, “Is it a...” What will I call it? Maybe charm.

Interviewer: So a charm? Like a spell?

Respondent: Yeah.

Interviewer: So is that kind of black magic?

Respondent: Yeah.

Interviewer: So what do you mean? So they thought it was a charm.

Respondent: Yeah. Some people were saying that, so I was asked to go for herbal treatment, not medical treatment.

Interviewer: So what do you mean?

Respondent: And I did. I went for herbal treatment.

Interviewer: Where was that?

Respondent: I went to Calabar.

Interviewer: What is Calabar?

Respondent: It’s a state in Nigeria. It’s at the east side of Nigeria.

Interviewer: Why did you go there?

Respondent: I was asked to go there. Somebody told me to go there.

Interviewer: Who did you see?

Respondent: I went there, I saw a woman, and she prepared some things for me, one to drink and one to apply on my skin.

Interviewer: Was that to do with the idea that it was a spirit or why was that? Who was that woman?

Respondent: She collected some things like leaves and other things, it’s herbs she gave to me and I used it, and I used it, it worked for me, because that period when I was using it I didn’t notice any new patches on my skin anymore, so it stopped. When it stopped I was kind of happy because it wasn’t spreading anymore at that time, until 2010 when I had my baby, then the whole thing started again.

Interviewer: So after the birth of your baby it started again?

Respondent: Yes.

Interviewer: How did you feel then?

Respondent: I felt very bad.

Interviewer: So very sad again?

Respondent: Yes.

Interviewer: I’m wondering, when your friends and family said they thought it might be a charm or a spirit, what did you think?

Respondent: I thought the same thing.

Interviewer: Can you tell me more about that? So what was the charm, or what was the spirit?

Respondent: I really don’t know. I only know even when I go into the woman’s place she never told me anything like that, all she said was she was going to give me herbs to be using, and I collected them and I came back to Lagos and started using it. And it really worked for me because at that period I was using I wasn’t seeing any more patches on my skin. It was just very little on my fingers like this. It wasn’t on my legs or any other part of my body, just the fingers.

Interviewer: And now, do you still believe it’s a charm or a spirit?

Respondent: No more, I don't believe anymore.

Interviewer: Why not?

Respondent: Since I met Ogo.

Interviewer: So since you met vitiligo, the vitiligo kind of support group that helped you?

Respondent: Yes.

Interviewer: So that changed your view? Ogo stopped you thinking it was a spirit?

Respondent: Yes.

Interviewer: And what do you now think it’s caused by?

Respondent: When I met her she gave me some pamphlet to read. I read them and I got to know more about it. So I took my mind away from that and the things she told me to be using I’d be using them and I think... that’s it.

Interviewer: So did you find the pamphlets helpful?

Respondent: Yes, I did.

Interviewer: I’m wondering, when you told your parents, what did they say?

Respondent: They believed too, and –

Interviewer: They believed it was a spirit?

Respondent: No. When they saw the pamphlet, when I took it to them I showed everybody, my parents, my siblings and those around me, they are like, “Oh God, is this really what happened to you? Don’t worry, God will help you, you will get well.” People keep encouraging me that way.

Interviewer: So now what do you call it, do you call it vitiligo?

Respondent: Yes.

Interviewer: Have you ever called it anything different?

Respondent: No.

Interviewer: I’m wondering what impact does vitiligo have on your daily life now?

Respondent: It makes me feel ashamed.

Interviewer: Why do you feel ashamed?

Respondent: I think I’m different from others.

Interviewer: So you feel different?

Respondent: Yeah.

Interviewer: So you don’t feel the same as others?

Respondent: Not that I don’t feel the same but that my skin is not the same with my siblings, my parents, my friends. Among them I look different.

Interviewer: So you look different to your family?

Respondent: Yeah.

Interviewer: So does that change your identity?

Respondent: No.

Interviewer: So your identity is the same?

Respondent: Yeah.

Interviewer: How does it impact on your relationships with your family?

Respondent: The relationship has been (inaudible 00:08:09), there has not been any... I have been showed love and care and encouragement.

Interviewer: Love, care and encouragement from your family?

Respondent: Yes.

Interviewer: How about your friends, has it impacted on your relationship with your friends?

Respondent: My friends too, they have showed me love, care, and also encouraging me.

Interviewer: I’m wondering has it had any impact on your romantic relationships?

Respondent: I’m not (inaudible 00:08:36) going into a relationship.

Interviewer: A long time ago you got into a relationship?

Respondent: Yeah.

Interviewer: So it hasn’t impacted your relationship?

Respondent: I’m not into any relationship.

Interviewer: You’re not in a relationship?

Respondent: Yes.

Interviewer: In the past has it affected your relationships?

Respondent: When I met my first boyfriend and then I had the vitiligo, I told him, but then I was always putting on long shirts, wearing something very long to cover my arms where the things appeared. So I told him, he said he doesn’t care, that he loves me, and it started but along the line we departed.

Interviewer: Because of the vitiligo?

Respondent: He really didn’t tell me why.

Interviewer: He never told you why?

Respondent: Yes.

Interviewer: Do you think it was because of the vitiligo?

Respondent: Partially.

Interviewer: And why is that?

Respondent: Because he never told me that, he has never mentioned it. Also because he told me not to be ashamed of it, that God will help, he was praying for me that God would help me that the vitiligo would go away from my skin.

Interviewer: I’m wondering has vitiligo impacted on your work?

Respondent: Yeah.

Interviewer: Do you mind me asking what do you do?

Respondent: I teach.

Interviewer: Young children?

Respondent: Yes. Young children.

Interviewer: So you’re a primary school teacher?

Respondent: Yes.

Interviewer: How does it impact on your work?

Respondent: To the children sometimes they come to me and say, “Auntie, what is this on your skin?” I tell them it’s skin discolouration. And some of them say, “Auntie, what are you doing about it?” I say I’m going for treatment.

Interviewer: What do they say?

Respondent: They’re like, “I hope that God will heal you.”

Interviewer: Does it have any impact on the parents of the children?

Respondent: Some of them.

Interviewer: What do they do?

Respondent: Some won’t want to associate with you or fear your presence.

Interviewer: Do they put pressure on you? Do they worry about you teaching their children?

Respondent: They've never said that to me. Because the proprietor has never shown me that he doesn’t want it or that he’s not happy with my condition, he has never showed that to me, so I don’t worry what the parents’ feelings, what I’m worried is just the owner of the school, the one who employed me.

Interviewer: Have they said anything? Is the owner of the school supportive of you?

Respondent: I don't know.

Interviewer: But they've never said anything?

Respondent: Yes.

Interviewer: But you worry about it?

Respondent: No I don’t.

Interviewer: You don’t? Okay.

Respondent: Yes.

Interviewer: I’m wondering, how does vitiligo impact on your thoughts? On your thoughts every day?

Respondent: Every day I wake up, see myself with the vitiligo and thought, “My God, just see me through. I want a day that I will look at my skin and I will not see these things anymore.”

Interviewer: So you hope that day will come when you no longer see vitiligo. I want to ask you about how you cope every day. And I know you've mentioned God a number of times. Does God help you cope with vitiligo? Can you tell me how?

Respondent: I just pray.

Interviewer: You pray?

Respondent: Yeah.

Interviewer: And does that help?

Respondent: Yes.

Interviewer: I’m wondering are there any other things that help you cope?

Respondent: Prayer.

Interviewer: Prayer is the big thing. Do you find that your family helps you cope and your friends help you cope?

Respondent: Yes.

Interviewer: You mentioned also that the vitiligo society has helped you cope as well, VITSAF has helped.

Respondent: Yes.

Interviewer: Are there any other things that have helped you cope?

Respondent: My friends. My cousins and my family.

Interviewer: How do you manage vitiligo?

Respondent: I try to live on fruits and vegetables and taking my drugs.

Interviewer: So those are the big ways that you manage it, so your diet, fruit and vegetables, and then taking the drugs that you mentioned, the creams and the vitamin supplements and the herbal supplements.

Respondent: Yes.

Interviewer: And you’ve said that spirituality helps you manage it, and your friends.

Respondent: Yes.

Interviewer: I’m wondering your church, do you go to church, do you go once a week do you?

Respondent: Once a week, every Sunday.

Interviewer: Have you spoken with your pastor?

Respondent: Yes, my pastor.

Interviewer: Is your pastor supportive?

Respondent: Mm. He’s given me some prayers and praying for me, and also telling me to use more of lemon, should squeeze out lemon and be drinking it.

Interviewer: So your pastor tells you to drink lemon, and have you done that?

Respondent: Yes.

Interviewer: And did it help?

Respondent: I’ve been taking it, it’s helping.

Interviewer: Do you have a good relationship with your pastor?

Respondent: Yes.

Interviewer: Thank you very much. I know this is a very difficult thing to talk about because it brings up lots of personal things, I just wanted to check you’re okay and you’re happy to go on?

Respondent: Yeah. I’m okay and happy.

Interviewer: Okay, thank you very much. I’m wondering how do you manage other people’s responses?

Respondent: Well, people don’t really talk about it when they see vitiligo, just a few they will come to you to ask the question what happened to you.

Interviewer: So people come up and ask you.

Respondent: A few.

Interviewer: Just a few people. And what do you say?

Respondent: I told them and they were like, “Keep praying, God will see you through.” Everybody just said, “Keep praying, God will see you through.”

Interviewer: So everybody says keep praying?

Respondent: Yeah.

Interviewer: I’m wondering for instance when you're walking through the streets do people react?

Respondent: Yes.

Interviewer: What do they say?

Respondent: They just look.

Interviewer: How do you manage that?

Respondent: I don't look at their faces.

Interviewer: So you look down and just keep going about your business? Okay. And you've mentioned that you’ve tried a number of treatments with the creams and the tablets that you're taking now, have you ever tried anything else?

Respondent: No. No, the one I’m taking is a woman giving me some herbs to take and she gave me some Forever products. Those are the ones I mentioned earlier.

Interviewer: What are they then? Is that a brand, Forever?

Respondent: Yeah. And she gave me aloe vera gel cream and I also applying on the skin. That’s what I’m doing for now.

Interviewer: And you're finding it’s helping?

Respondent: Helping, yes.

Interviewer: I’m wondering if I could ask you whether you think vitiligo impacts on your life, would your life be different without vitiligo?

Respondent: Yes.

Interviewer: How would it be different?

Respondent: Because I would have achieved some success but it has really limited me because after spending so much money on it and it’s not really gone, so all my focus is just on it, how to get myself well again for now.

Interviewer: So that’s the main thing, you've had to focus.

Respondent: Focus on, yeah.

Interviewer: So you focus a lot on it?

Respondent: Yes.

Interviewer: So if you hadn’t had it then you wouldn't have focused so much, you wouldn't have spent money on it. You mentioned you had a child?

Respondent: Yes.

Interviewer: Is it a boy or a girl?

Respondent: It’s a boy.

Interviewer: How old is your son?

Respondent: He’s two years and six months.

Interviewer: What’s his name?

Respondent: Richard.

Interviewer: It’s a nice name.

Respondent: Thank you very much.

Interviewer: So I’m wondering if you could just tell me, how do other people react to your vitiligo?

Respondent: Some do want to (inaudible 00:19:02) to associate it with it much, walk with it too much, some of them some don’t mind. I have a friend, Edith by name, since ever vitiligo started she has always telephoned me and this has never from one day discouraged her and I so much, whenever I see her I’m so happy that she’s given me courage and boldness.

Interviewer: So she helps you a lot?

Respondent: Yes, she helps me a lot.

Interviewer: And that’s your one friend, she’s very, very helpful?

Respondent: Yeah.

Interviewer: So how do other people react?

Respondent: I don’t know because I just go to work, come back, stay in my house and I don’t come back to associate with people so much. I can stay inside and do what I have to do.

Interviewer: So you don’t go out so much?

Respondent: I just go into work, go to church.

Interviewer: So you go to work and you go to church but you don’t go out socialising?

Respondent: No.

Interviewer: Because of the vitiligo?

Respondent: Yeah.

Interviewer: So it stops you living the life you might have left?

Respondent: Hm (agreement).

Interviewer: I’m wondering if there are any positive things about the vitiligo?

Respondent: I’d say it has really helped me to check myself, it has really helped me to grow more closer to God.

Interviewer: To grow closer to God?

Respondent: Yes.

Interviewer: What do you mean by check yourself?

Respondent: I has really helped me to draw closer to God like I said and always check myself by checking some ways that maybe I do things that are not right trying to make sure everything I do in my life is okay and perfect.

Interviewer: So you’re very conscious about making your life okay and perfect and trying to make it as good as possible?

Respondent: Yes.

Interviewer: And the vitiligo’s helped you do that?

Respondent: Yes.

Interviewer: Why?

Respondent: Because I don't want people to start seeing faults in anything I do.

Interviewer: So it focused you so nobody can pick fault in you?

Respondent: Yes.

Interviewer: Thank you so much, Grace, for answering all my questions. I just have one more question for you if that’s okay. So I’ve asked you lots of things and I wanted to ask whether you think I’ve missed anything. Is there anything I should have asked you about which I haven’t?

Respondent: No, you've asked everything.

Interviewer: You’re happy that I’ve asked enough questions?

Respondent: Yes.

Interviewer: Thank you very much for taking part, I’m going to stop the recording now.

**Participant 4- FA**

Interviewer: Can you tell me please, when did you first start noticing changes in your skin?

Respondent: It was 14 years ago.

Interviewer: What happened? You mentioned that you had an accident.

Respondent: I had an accident, so after I was in the hospital for a while, a month. So after leaving the hospital, after a year or thereabouts I started seeing white patches on my body, I think for my arm there.

Interviewer: So about your arm and your hand, about there you started seeing them?

Respondent: No it started from my arm. So as time goes on, you know, I was taking some medication. I have it on my face too, a smudge on my face, so I was taking some medication, you know? Basically vegetable, I was taking a lot of vegetable.

Interviewer: That was when you first noticed it you started taking vegetables?

Respondent: So I discovered that there’s a small black patches on the white patches, that it’s coming little by little. You know, it was coming out, just coming out, it’s coming out. So (inaudible 00:01:36) I had vitiligo this my face, you know.

Interviewer: So how quickly did it go from just being a few places on your arm and hand to being on your face?

Respondent: When I stopped the medication, this was coming up then. Later whenever I stopped it.

Interviewer: Which medication did you stop?

Respondent: I was taking more vegetable. At times fruits, you know, all these fruits, but I stopped, you know. There wasn’t time for me to get the fruit and other stuff because I was out of Lagos then, you know, (inaudible 00:02:32) work. So I was able to get the (inaudible 00:02:36) of the fruit, I usually take there.

Interviewer: So when you first started noticing it your first reaction was to start changing your diet?

Respondent: No, I was taking my diet, I didn’t change my diet. Vegetable is part of my diet. You know, I use vegetable to make soup now, you understand? Vegetable soup.

Interviewer: So you first noticed them, and who did you tell?

Respondent: My mum.

Interviewer: What did your mum say?

Respondent: You know, she was worried then that, what happened? You know. She like to go to the hospital. Then she took me to the hospital. They were to see me for something else, you know, they just say sort of things that it’s not down with me, you know? So my dad now saying that should not take me to the hospital again. My dad said that he doesn’t want the doctor to inject me. You understand? Now all the injections they are just giving me is not working. I stopped the injection then.

Interviewer: So that was very early on, they started giving you injections.

Respondent: I stopped all the injections, it was not working.

Interviewer: How long were you taking injections for?

Respondent: Close to a year. I was going to the hospital for medication.

Interviewer: So that was the very start, was it? You found it and then they started giving you injections?

Respondent: And then the white patches were just coming out still, you know, no difference, so it wasn’t enough taking injection.

Interviewer: So I’m wondering how were you feeling, when it first appeared how did you feel?

Respondent: Well I felt bad because I was not born with vitiligo, I felt bad. People are with me then say, “What happened to you? What happened to you?”

Interviewer: So people started asking?

Respondent: Asking. Giving out questions.

Interviewer: So when you said you felt bad, were you feeling depressed, what were you feeling?

Respondent: Then I felt depressed a lot, you know. I was in school then, I have to cover my body, until after some years when I built the confidence.

Interviewer: So for a lot of years you were covering your body and trying to hide it were you?

Respondent: Yes, and it was still coming out. Then I got to the stage I decided not to cover my body again.

Interviewer: You don’t want to cover your body now?

Respondent: No, I decided not to cover my body again.

Interviewer: What made you decide not to cover your body again? What changed?

Respondent: What that was, I felt that yes, I have vitiligo, you know, there is no doubt about it, I can’t cure myself, I can’t cover vitiligo. You know, it’s better for me to live with it. You understand?

Interviewer: Yeah.

Respondent: In the end of the day I have vitiligo, you know, my friends and everybody was with me, so I stay by my home, you know, I feel free with everybody, you know. In the process I have other stuff you know. My wife knew that, yes, I have vitiligo, and she knows about everything.

Interviewer: So were you married to your wife when it first appeared or did you meet her after it appeared?

Respondent: No, I had vitiligo then, I had it on my body. She asked me like, “What happened to you?” and I explained to her. I explained to her. I usually bring my wife to most of the conferences I usually have so that she can hear more about vitiligo so that she can learn more about it.

Interviewer: I’ve heard that you’ve used the term vitiligo to refer to it, but before you knew it was vitiligo what did you used to call it?

Respondent: Then I called it white patches, because I didn’t even know the name then. White patches. But when I met Ogo then it’s vitiligo, you know. After then the nurse said I have to go the net and make my own research, and that is what I did.

Interviewer: So once you heard the term you went away and researched it?

Respondent: Yeah, research. I now discovered my skin is not that pigmented. So I discovered that yes, it’s true, I have vitiligo.

Interviewer: I’m wondering what impact it has on your life.

Respondent: Well to me it doesn’t have any impact. I live my normal life. But even if I’m going in the street people usually size me, you know, a lot. Size me, I size you, I look at your back, you know.

Interviewer: So you feel they’re sizing you up, they’re kind of checking you?

Respondent: Yes. You know, even there are some people that even walk towards you and ask you that, “This is this?” I say, “No, it’s not it.” “Maybe my fore-fore-fore-father has.” I say, “No, it’s not, it’s vitiligo.” Said, “Okay.” You know.

Interviewer: Has it impacted on your family, on your relationships with your family?

Respondent: It doesn’t have any impact on me.

Interviewer: On your work.

Respondent: It doesn’t have any impact.

Interviewer: Has it ever had an impact?

Respondent: It has not.

Interviewer: In the past did it have an impact?

Respondent: No. It doesn’t have any impact.

Interviewer: I’m wondering if it has any impact on your feelings at all or on your confidence or on your thoughts?

Respondent: You know at times if I look back at my pictures, then. I will look at myself, I was not born with this thing, with vitiligo, but now I have it. I have to live with it, and manage it. The first person I asked, “Is there any cure for vitiligo?” At least if there’s any cure for vitiligo at least I will try and get the medication, if there is any cure for it. When I heard that there is no cure for it for now, that you can just only manage it.

Interviewer: So that’s one of the ways that you do manage it is that you kind of say, if a cure comes, great, I’ll take it, if not I just live with it?

Respondent: If not, yeah.

Interviewer: So for you one of the ways to cope is to kind of accept?

Respondent: That is it.

Interviewer: What are the other ways that you manage it? How do you manage people’s reactions?

Respondent: I have a lot of reactions, you know. I have a lot of reactions. Because I myself I don’t even allow their reactions to bring me down.

Interviewer: So you purposefully don’t allow other people’s reactions to get you down?

Respondent: No. Have confidence of myself with everything I’m doing. So you know, if you don’t like me don’t like me, if you like me, like me, that is it.

Interviewer: But confidence is very important?

Respondent: It’s very important. Even though I work I have confidence with myself because I know what I’m doing. I know it best at best, so should have confidence in me or I will lose job, look for a job somewhere else.

Interviewer: What does confidence mean to you, or what is being confident?

Respondent: It’s sort of a self-confidence, it’s not pride, you know? It’s self-confidence in me. Even at home, you know, at times I look at my picture back, you know, my wife tell me that, “See, I knew that you have vitiligo, I knew that you have vitiligo. That is not the reason I got married to you.” So I just have to cope with it, you know. Since I’m not... it’s not a disease, you understand? So you know, for some years I am not really sick or anything, I’m okay, so it’s not something that I... it doesn’t itch, nothing.

Interviewer: Has it ever had an impact on any of your romantic relationships or has it never been a factor?

Respondent: Not at all.

Interviewer: Not at all?

Respondent: Yeah. Everything’s perfect, not at all.

Interviewer: And I’m wondering whether you manage it using any physical treatments. You said you tried the injections but they didn’t work.

Respondent: They didn’t work. Even the so-called drug melanin I know this name. I tried it, it doesn’t work.

Interviewer: And it doesn’t work?

Respondent: It doesn’t work. I’m just wasting my time on the drug. So later I now ordered for this raculia(?) drug.

Interviewer: I’m wondering about spirituality, whether that’s played any role in your experience of vitiligo?

Respondent: It doesn’t play any role.

Interviewer: It doesn’t. It’s not relevant to you?

Respondent: It wasn’t relevant to me. It was then people talk about spirituality and all that stuff. (Kisses teeth) forget about it. You know, it wasn’t relevant to me.

Interviewer: Because I understand in Nigeria there are some people who believe that when bad things happen or when people get ill it’s a spirit.

Respondent: Yeah, there are some people.

Interviewer: Have people ever said that to you or is that something you’ve experienced?

Respondent: They were saying that a lot, you know. They’ve said that a lot. One thing about me, I’m the kind of person I don’t listen to what people say. Even if it’s not about the spirituality it’s do this, do this, you know, kind of maybe try to make one or two sacrifice or this thing. I’ve tried it in the past, doesn’t work, so I don’t have any confidence in it any longer, so.

Interviewer: I’m wondering about when you walk down the street, how do you react to people in that environment, or how to people react to you?

Respondent: People stare at me a lot, a lot, even on the street. Maybe if I’m walking on the street people say, “Ah, what happened to this fine man?” I was walking on the street, a lady will come to me and say, “Ah, you look awesome.” I say, “How?” I said, “Are you kidding me?” She said that, “This thing you have on your body makes you fine.” I said, “Thank you.” You know? You know, for her to come and meet me I know they might have discussed one or two, you know, for she to have the confidence to come and meet me, and she asked what happened to me and I told her, she said, “Well you look fine.” She asked me if I am married. I said, “I’m married.” She said, “Okay.”.

Interviewer: How did you find that experience?

Respondent: (Laughing) Well it was somewhat challenging to me, I don’t look at that, you know.

Interviewer: So whilst it was challenging but you don’t let it bother you?

Respondent: No, not at all.

Interviewer: What do you say to people, you say it’s vitiligo, or what do you say?

Respondent: I say vitiligo.

Interviewer: And then they say, “What’s that?”

Respondent: They say, “What’s that?” You know. I try to educate them about vitiligo and you know. Some will be saying something else, you know, beyond the vitiligo, you know.

Interviewer: And the people, do you get the sense they’re worried, or what... when you see them looking at you what do you see?

Respondent: You know, you see people looking at you from afar, from a long distance. You see people looking at you. But I realise people are looking at me, but I just have the confidence and I have to face what I’m doing, I don’t look at them. Even though at times if maybe I’m in a social gathering or something, you know, some ladies will be staring and saying, “Look at that guy, look at that guy.” It’s fine. Last time my wife caught one of these social girls, saying, “Ah, this guy, what happened to him?” You know. My wife heard what they were saying. My wife have to challenge them, like what happened. “Why are you looking at my husband?” You understand? In a social gathering.

Interviewer: And what did they say?

Respondent: They were saying, “What happened to this guy? Is it hot water upon his body?” You know, all sort of this, you know. My wife heard what they were saying. My wife have to go and meet them and shun them like, “What are you saying? Leave my husband alone. Can’t you see that he’s with the wife.” You know.

Interviewer: How does it impact on your wife? Does that affect your relationship when she’s having to challenge people or does it...?

Respondent: It doesn’t. It doesn’t at all. Because she knows everything about me. Nothing.

Interviewer: Do you have children as well?

Respondent: Yes.

Interviewer: How old are your children?

Respondent: I have a daughter.

Interviewer: How old is she?

Respondent: January 14th she will be two, yes.

Interviewer: So she’s very young.

Respondent: Yeah.

Interviewer: Does she notice? Does she ask? Because she must be talking now is she?

Respondent: She is small.

Interviewer: She’s very small? Too small?

Respondent: Yeah. She’s still a baby.

Interviewer: Sleeping okay?

Respondent: Ah. (Laughing) Very active.

Interviewer: Very active, yeah, lots of energy.

Respondent: Very active.

Interviewer: I’m wondering are there any other treatments that you’ve tried that you’ve not told me about that we’ve not discussed? I understand some people sometimes use makeup, and is that something you’ve ever tried?

Respondent: That is what they say, this camouflage. I have not tried it. To me I just say, “How long will I be using makeup, be using camouflage?” You know. (Inaudible 00:18:25) can’t say that I’d even have time, to be using make up and all that stuff. With this whether the (inaudible 00:18:36) I’ll be using make up. You know, I’m not used to it.

Interviewer: It wouldn't be comfortable.

Respondent: No, no, I’m not comfortable with it.

Interviewer: Are there any other things that you’ve tried?

Respondent: Nothing. For some years I didn’t take any medication. I didn’t even managing my vitiligo until when I now (inaudible 00:19:08) for this reculia(?), the drug, the supplement, I was using the supplement, you know? They said one tablet a day. The supplement is a combination of garnica(?) acid, folic acid, vitamin E and C.

Interviewer: And do you find it’s helped?

Respondent: Well, they said constant using it, you know, that if I’m using (inaudible 00:19:42) you know, at least the vitiligo for at least you have to be using... is it folic acid, Ogo?

Ogo: Yeah, folic acid. 400 to 800.

Respondent: 400 to 800 a day.

Interviewer: And you order that online, do you?

Respondent: What is that?

Interviewer: Where do you buy that from?

Respondent: I got it from America, from the States. I ordered it. A friend of mine picked it up and brought some down to me.

Interviewer: I’m wondering how do other people react to your vitiligo?

Respondent: Well, they react positively. A lot of people react, they talk, about what is happening to this guy? You know. Even though at times it’s somebody that they’ve known me, if the others describe me they describe me, “That guy that has something on his body.” Do you understand? But to me I know I have something, I have white patches on my body so I don’t allow that to bother me.

Interviewer: So it doesn’t affect your emotions, it doesn’t make you sad?

Respondent: No, not at all.

Interviewer: I’m wondering how you found the kind of medical care, dermatology care in Nigeria.

Respondent: Actually I went to meet a consultant, (inaudible 00:21:21) leading(?) hospital(?). So then he was telling me that I should be coming for the clinic I think every two weeks, to be taking melanin and all that stuff. That is the time I met, I don't know maybe he’s a professor or doctor, had this loot(?). He was telling me about something, that if I can try the other medication that there is one like that they will expose to my skin or something, to (inaudible 00:22:07) black. You understand?

Interviewer: So yeah, so there’s other options.

Respondent: Well me, I’m scared to take the other option, because it might... I don't know maybe it has other side effects, I don't know. I’m scared to take the other option. You know, that is one option they said in my case but that one is too expensive, so. But the one they said that you’re exposed to something, maybe the sun or something, but I can’t take that option because I don’t know the end result.

Interviewer: And the risk is too great.

Respondent: And then the risk. You know. I don’t know, maybe they want to do one or two experiments, they want to use me.

Interviewer: Thank you very much for answering all my questions, I really appreciate that.

Respondent: You’re welcome. Thank you.

Interviewer: Before we finish I just wanted to ask whether there is anything you think I’ve not asked, whether there’s anything I should have asked that I’ve missed.

Respondent: What I expected you to ask, you know, people are doing research, in Africa there are so many things that are (inaudible 00:23:34) that maybe if you have vitiligo maybe there is one (inaudible 00:23:43) sacrifice that the family has, maybe they have in their village, that they need to do one to sacrifice, that they do the sacrifice, that they will use this, what do they call it? There’s one called maybe oshibota(?) or something, that they use to rub the surface (inaudible 00:24:11) using it that the thing will go. For me I don’t really rub(?) that. I’ve tried that but it didn’t work.

Interviewer: So you did try that, did you?

Respondent: I tried (inaudible 00:24:19) or something but it didn’t work out.

Interviewer: Where did you try that?

Respondent: I did that in Lagos, but it didn’t work out.

Interviewer: What did they do? Can you tell me about that experience?

Respondent: The experience, they will make I think appeals, it goes, and do one of the sacrifice.

Interviewer: Then what do they then do?

Respondent: So after that they will now go back there and take the oshibota(?) to do this thing that they use to rub, but it didn’t work out.

Interviewer: So they do a sacrifice?

Respondent: It’s more or less like a sacrifice. They will appease this thing that maybe was the –

Interviewer: So they appease the spirits?

Respondent: – the spirit that maybe the fore-fore-father that they didn’t do, that they didn’t appease the spirit for some years, that is the one that is now affecting maybe one of the grandchildren or one of the great-great-grandchildren.

Interviewer: But for you, you said you don’t believe in that?

Respondent: No I don’t believe in that. I asked my dad, he said there is nothing that would have such a thing in our family. Some do have.

Interviewer: But for your family it hasn’t been?

Respondent: In our family we don’t have.

Interviewer: And you said that the church hasn’t been relevant either.

Respondent: The church?

Interviewer: Yeah, is that right? You said God hasn’t really been a factor in...

Respondent: No, I don’t see that, you know. With God all things are possible. You understand? If maybe I went to the pastor and he prayed for me and God said, “Okay, if that’s (inaudible 00:25:58) fine.” You understand?

Interviewer: I do understand. I misunderstood earlier because I was wondering whether the church has been supportive of you with vitiligo and whether that’s been an important place for you to find kind of suppose.

Respondent: Yes, in everything I do you put God first. I usually go to church, on every Sunday and pray to God. Because this is not how God created me. You understand? So I pray to God, if God created me (inaudible 00:26:37) wouldn't have any vitiligo. God shall return my skin to my normal complexion. I pray to God to help(?) me. Spiritually can heal. Anything can happen.

Interviewer: So through prayer healing can happen, through God?

Respondent: Yes. With God all things are possible.

Interviewer: Thank you very much.

Respondent: No problem.

Interviewer: I’m going to stop for this section.

[End of Transcript]

**Participant 5- FA2**

Interviewer: Can you tell me please, when did you first notice your skin was changing?

Respondent: When I was in school, GS2, it started in my face like a pinch.

Interviewer: So what do you mean like a pinch?

Respondent: Just like a pinch, something like pimples. Yeah, in my face. So from my face to my neck, from my neck to my fingers. So from then my daddy took me to hospital to check. They said there’s no drugs from there. From there we went to the village, they said we should use native drugs. We used native drugs, nothing. The drugs did not work. So from then my daddy said, “Okay, we should just hold off for some time.” So when I go to school I always get ashamed, people call me different type of names. Hot water, fire bomb, you understand? So after my secondary school the one in my neck left just like that, that wasn’t anything, but from there started coming to my hand. My hand started turn to white, my waist to my leg. So when I was going to VI one time ago I saw somebody that said there is somebody who has vitiligo that he would like me to meet (inaudible 00:01:32) if I want Nigeria to know so that they can interview me. I said yeah I would like to know the person. Then I met Ogo.

Interviewer: When was that, how long ago?

Respondent: Let me see. Auntie Ogo, is it now for two years now or three years?

Ogo: About three.

Respondent: Three years now, yeah.

Interviewer: So going back to when you were 10, 11, you said the kids at school would call you names.

Respondent: Yeah, different type of names. Hot water, fire bomb. After then I was into a relationship with somebody.

Interviewer: At what age was that?

Respondent: Let me say I was 20, 21. Yeah. So from there the person knew that I have it in my face, you understand, but he don’t know that I have it in my leg because I always wear trousers I don’t put on skirts. Then I was ashamed. I don’t like coming out with trouser and with skirts. So I did not have and every time it’s shirts, I cover my hand, my body.

Interviewer: So that was when you were younger, that’s what you used to do?

Respondent: Yeah.

Interviewer: Did you do that when it first got diagnosed as well, when you were 10, 11, did you start covering up?

Respondent: No. I used to wear something longer that would cover my body, but when I met Ogo, Ogo gave me the courage, so from there I started wearing spag(?) skirt, now I can wear a skirt to church, but then I don’t wear skirt to church, I cover my body, I wear long skirts, not shorts.

Interviewer: I’m wondering, so you talked to your mum, that was the first person you told or your dad?

Respondent: My dad, because I don’t have a mother.

Interviewer: You don’t have a mother so you spoke to your dad.

Respondent: Yeah.

Interviewer: And he said, “I think we should go...”

Respondent: Yeah, he took me to hospital. So the hospital they didn’t have the drugs, they said for now they don’t have the drugs. So I said, “Okay.” So we went to a chemist, chemist gave us calabsu(?) something like calabsu(?) but the thing was that –

Ogo: Ketacalubsu(?).

Respondent: So it was affecting my face so we stopped it.

Interviewer: How did your dad react to all of this?

Respondent: See now my daddy is thinking about it, it’s in there, but to me I don’t think about it again.

Interviewer: So your dad still thinks about it?

Respondent: Yeah, that’s it.

Interviewer: What does he think?

Respondent: That I’ve got vitiligo. Sometimes he thinks that maybe it’s somebody that did it, or maybe it’s from the village or maybe it’s disease. Sometimes I just tell him that, “It’s not disease, don’t worry. One day this thing will go.”

Interviewer: What do you think about the idea that somebody from the village might have done it or it might be a spirit, what do you think about that?

Respondent: My mind did not go there to the village that maybe somebody did it. Why? It’s because I didn’t offend anybody there. There is different type. Why I didn’t put my hope that it’s not somebody that did because it just came like that. You understand? It’s not that when it came it was itching me, no. It just came just like if something want to change my skin, just like that.

Interviewer: Does your dad... your dad still wonders?

Respondent: Still now

Interviewer: And how is that? Does that create a conflict between you?

Respondent: Sometimes we have misunderstandings. Me, I don’t want to follow him out that we should be going to church, different type of church, so that any man of God can heal you. So I say, “No, I have the faith that one day the thing will go, I don’t like going to different type of church every day. No.

Interviewer: But your dad wants you to go to all the churches to find somebody to heal it?

Respondent: Yes.

Interviewer: What do you think about that?

Respondent: My pastor is okay. When I have the faith God can heal me in my house. He’s not go to the church, yes he can use the man of God to heal me, you understand, but for me it will... since I have seen people that have it and somebody explain more better to me about vitiligo, so since then I will move my mind from there.

Interviewer: Yeah, so since you found out more it’s been easier?

Respondent: Yeah. He gave me more mind. Before every time I would be ashamed, I don’t like sitting down with people.

Interviewer: Why not?

Respondent: Because of vitiligo. But now I can sit down with a crowd.

Interviewer: And you don’t mind, you’re happy?

Respondent: Yeah, happy.

Interviewer: So you call it vitiligo?

Respondent: Yeah.

Interviewer: Have you always called it vitiligo?

Respondent: Then I don’t call it vitiligo.

Interviewer: What did you call it?

Respondent: Then I call it fire... because people call me fire bomb, I don’t even know the name.

Interviewer: Fireball?

Respondent: Fire bomb.

Interviewer: Bone?

Respondent: Bond. So people call me hot water. That means hot water that pour on me. But me, I don’t call it any name.

Interviewer: I’m wondering what impact it has on your life now, what impact does vitiligo have today?

Respondent: It’s part of my life. I’m living with vitiligo not that I’m happy. I’m not so happy for me to have it but I thanking(?) myself. You understand? So I don’t have another thing to do that let life continue. Maybe one day I may sleep and wake up I didn’t see it again, fine, but for now with vitiligo so I just need to have the impact and live with vitiligo for now. Yeah.

Interviewer: How does it make you feel?

Respondent: It makes me feel worse sometimes.

Interviewer: Worse?

Respondent: Okay. It make me to feel okay sometimes. I sometimes in the place I work I work in a hotel, like last week it was Christmas period, there is a girl that came from Jamir(?), I wanted to serve the girl, she said, “No, no, somebody has to come, you have vitiligo.” So I need to go back to the room and tell the girls that, “You don’t know about vitiligo and you are telling me to not serve you, I should not serve you water.” They (inaudible 00:07:46) vitiligo.

Interviewer: How did that feel?

Respondent: It make me to feel bad just because after everything the girls report me to my GM for na(?), it’s just like I’m losing my job.

Interviewer: Are you losing your job?

Respondent: For now because of the case, because the girls need to go and tell my GM that this girl is not good to us and my GM know that I’m not good but for him to just change like that, that they should give me suspicion. So since then I am suspicious about (inaudible 00:08:19) because I know if they say that I should not come back to that place I will have another job, but it is hard for me, somebody that have vitiligo to have a job because before I got this job it was hell.

Interviewer: It was hell before you got it?

Respondent: Yeah.

Interviewer: What were you doing before?

Respondent: Before I was at home helping my dad, because I go to a different type of place to work. The first place I work then because of vitiligo they didn’t collect me. You understand? They didn’t call me for the job. But I submit my CV there, but this one they called me for the Sheraton. I did my interview in the Shereton, yeah, so they took me to the Sheraton in VI, but for na(?), I don’t know.

Interviewer: So you don’t know if you have a job waiting for you?

Respondent: Yeah.

Interviewer: Because you went to serve someone and they said, “You can’t serve me.”

Respondent: Yeah.

Interviewer: And now your general manager said you can’t do that.

Respondent: Yeah.

Interviewer: And now you might not have a job.

Respondent: Yeah.

Interviewer: Wow. That’s not easy.

Respondent: The problem there is that, fine I’m not supposed to argue with my customer, to my guests, I’m not supposed to talk to my guests, but on the other side I’m supposed to let me to know more about the guest, let the guest know about vitiligo because they may go to any other hotel and see somebody that has vitiligo, they may lead the person to lose their job. That is why I went back to the room to explain better to the guest.

Interviewer: What did the guest say?

Respondent: She said that I should not worry. But after that I don't know, they(?) went to tell my management.

Interviewer: What did the management say?

Respondent: Since then I have not been at work. When I call they’ve said that they are still talking about the case.

Interviewer: Are you worried?

Respondent: Some are worried. I’m worried. I can’t say that I’m not worried, I’m worried because I don’t like sitting down at home, I like working.

Interviewer: When did this happen?

Respondent: Last two weeks.

Interviewer: How has it impacted on your mood?

Respondent: It doesn’t make my mood to be like before now. Throughout yesterday I didn’t come outside, I was at home.

Interviewer: All of yesterday?

Respondent: I was at home. I didn’t come outside to say let me take fresh air, no.

Interviewer: Why?

Respondent: Because I was at home just sitting down thinking that I don’t know how to get another new job again this year, but I know God will do it.

Interviewer: So you have faith in God?

Respondent: Yeah.

Interviewer: That you’ll find a new job, that there will be...?

Respondent: Yeah.

Interviewer: So are you resigned to the fact that you won’t be going back?

Respondent: Yeah. That means the HR called me when I was in the east that I should write resigning letter to them, and they are telling me to resign, that they don’t want to sack me I should resign. So latest Friday I will give them the resign letter.

Interviewer: Right. Do you feel angry?

Respondent: It makes me feel bad just because, yeah, they said that means customer is always right, but on the other side customer is not always right because there are some customers that lie. You understand? There are some customer that they just want to cause problems so that you can wire(?) them. You understand? There’s some customers that (inaudible 00:11:56) you do not do. Like the other time I have misunderstanding with a customer in the room that they said money is missing, and money is not missing, I happen to see your money on the floor, I gave it to you. The next thing he said I took it from your wallet. You understand? If I took it from your wallet I would not give it to you that I saw your money. You understand? The next thing say you have a camera in your room, okay show me the camera so that you have the proof that I took it from your wallet, you can show me the camera. You understand? So people just come start talking, talking, talking, so I said, “Okay, fine.” I told my boss, “You know me for two years now, I’ve the same things I give to security,” you understand? The security people know me for me to be with the best staff of the year. Management supposed to know that person that look at what this person can do and look at what this person cannot do, so for you to just believe the girl that this girl said your staff stole the girl’s money, fine you will not take the girl say but you will go and talk to the staff, talk to the staff one-on-one, “Do you do it?” and you’re supposed to know what staff can do. Staff that you’re working with for two years first, you understand? So the girls were just talking that I had vitiligo, I would make sure that they sack you from this work. So when the GM think about the girls that I argued with and these guest that said that I stole his money, I know for the GM you don’t want me to come back. But for me I would like to work there again because I like working hospitality. You understand? But if they don’t call me back, fine, maybe God is to find something better than that.

Interviewer: So you have faith in God?

Respondent: Yeah.

Interviewer: I’m wondering how vitiligo’s impacted on your relationships with friends, with family, with romantic relationships.

Respondent: Yeah. My family most. Vitiligo makes me and my family to have misunderstandings, most my dad. Sometimes when I cook my daddy will not eat it, that he wants me to go to the church, what is this man church? Synagogue. My daddy even talk about it, that he wants me to go to synagogue, that he saw that man heal people that have vitiligo.

Interviewer: To the Jewish synagogue?

Respondent: Synagogue, church, yeah, it’s the church.

Interviewer: Sin of God.

Ogo: Synagogue.

Respondent: Synagogue.

Interviewer: Synagogue, so like a Jewish church?

Respondent: Yeah. That he saw the man heal people that have vitiligo. I said, “Fine, that one is different. This thing that is white, there are some people that vitiligo is different than those things. You want me to go there but me, my mind don’t want to go there, I don’t want to be worried about

this again. You should leave me alone.”

Interviewer: So your father was putting pressure on you?

Respondent: Yeah.

Interviewer: And you became annoyed with him and said, “Just let me be.”

Respondent: Sometimes we have misunderstanding, even I cook him said he’s not eating, but sometimes my other brother will bet me, talk to me that I should not worry, that God will do it. You understand? In my relationship sometimes guy thought that vitiligo is something bit like that is a disease. A girl 29 years I can’t tell you I have a boyfriend, I don’t have a boyfriend.

Interviewer: And you can’t have a boyfriend?

Respondent: I know I will have, I know somebody will come, you understand? But vitiligo led me to keep my body, you understand? Because I think if I don’t have vitiligo (inaudible 00:15:48) say they want to come and sleep with this guy and go just like that, because you will not know the guy that is serious about your relationship, but with vitiligo you know who will love you with what you are, with what you have in your body. You understand? I have met so many people and people just want to eat and go, but to me I say, “No, if you like me you will like my vitiligo, you’ll like who I am.”

Interviewer: So they have to accept it?

Respondent: Yeah. He has to accept it since I have it.

Interviewer: But it’s made it very difficult for you to find a boyfriend?

Respondent: Yes, somebody that will love me.

Interviewer: What about with your friends?

Respondent: Yeah, I have friends in the church, at work, sometimes people at work call (inaudible 00:16:35) this thing is disease, so then sometimes they say that this thing is sickness so I will just leave the lady to say whatever they want to say.

Interviewer: And that happens in the church?

Respondent: In the church sometimes when pastor said, “She shake,” I don’t shake.

Interviewer: You don’t?

Respondent: I don’t shake. But not that I don’t like shaking, I like shaking but since something like that happened to me, if we were in this same church you’ll know me better, I can shake you, but if you are a newcomer you just come in and you say, “I’ll shake you,” I will not shake you.

Interviewer: Why is that?

Respondent: Because some people when pastor says I should shake, when I bring out my hand I want to shake, because there is one time that one lady, I want to shake the lady in my church, just because he saw my hand he moved his hand back, but it’s a new member. He was afraid that, “Ah, what happened to this girl?” So me, I just move my hand.

Interviewer: How often do you go to that church?

Respondent: It is my full church.

Interviewer: So once a week?

Respondent: That means twice, three times in a week, we have bible study and prayer time, my Sunday is for studies.

Interviewer: Do you know the congregation well? So you know most of the people there?

Respondent: Yeah, it’s my church from little.

Interviewer: And do you have many siblings? You mentioned a brother.

Respondent: My brother.

Interviewer: Do you have any more brothers and sisters?

Respondent: I have, yeah, we have seven in number, I have brothers and sisters.

Interviewer: Are you close with them?

Respondent: Yeah, very, very close.

Interviewer: How do they relate to your vitiligo?

Respondent: They’ll go out to ask people about the vitiligo about the skin. Why some people say that she was ony that somebody told my sister that she was ony(?) but we didn’t ony to rub my body, like the thing will start changing. But sometimes I use the ony to rub, sometimes they say, “Well let me just leave, let me just do like that.”

Interviewer: So sometimes you feel that you just want other people to accept it?

Respondent: Yeah.

Interviewer: And are there any other ways, because it sounds like it’s impacted on your relationship with your father, it impacts on your romantic relationships and it impacts on your work life as well.

Respondent: Yeah.

Interviewer: And sometimes in church life as well.

Respondent: Yeah.

Interviewer: It sounds like it has quite a big impact in your life.

Respondent: Yeah. Sometimes if you’re passing on the road or inside a bus some people will just stress, “Look at this girl,” so people will just be looking at me. I will just be ashamed that, “Why are you looking at me like that?” So that I’ will say, “Is it because of my vitiligo?” Sometimes they ask me a question, “What is this?” I explain to them.

Interviewer: So on the street it also has a big impact?

Respondent: On the street, yeah. Sometimes on the bus.

Interviewer: Do you have any examples of times when it’s been really difficult on the street?

Respondent: From the street, yeah, that means there was everywhere. You understand? So I was putting on trousers and somebody beside me said, “Ah, there’s (inaudible 00:19:35) I should put on my trouser so that the rain will not fall on my trouser. I said no. Said no I should do it now. So when I said, “Let me turn my trouser,” he said, “Why?” He was telling the other person, “Hey, look at why this girl doesn’t want to draw the trouser, her leg is white, that thing is hot water.” The next day I put down my trouser, I stand up, I say, “Sorry, it’s not hot water, that is vitiligo.”

Interviewer: What did they think it was?

Respondent: Hot water.

Interviewer: How water?

Respondent: Yeah.

Interviewer: Because it would scald the skin?

Respondent: Yeah. Because that day my leg was wet so the thing was white. That is why sometimes I don’t like going into water if it’s raining, I don’t like walking into water if it’s raining because my leg will be white, my body will be white.

Interviewer: You said earlier that when you met Ogo you started being able to wear what you wanted and you covered up less, is that still the case now?

Respondent: No, now I can wear anything, I can put on spag(?). But then I don’t wear spag. Something like this now, then I don’t fold my shirt, I like anything with a sleeve that will cover my hand, because then I was ashamed of vitiligo.

Interviewer: But now you’re more relaxed?

Respondent: Yeah. But not like that because like last two weeks I was front of mirror, when I look at my face, wow, that means I also have vitiligo in my face, so I was just talking to my face that I need to give myself courage that even if it come to my face.

Interviewer: So in the last few weeks then since this happened in the hotel you’ve had a bit of a knock of your confidence, it’s affected you?

Respondent: Yeah.

Interviewer: Do you feel that you’ve gone back a few steps in feeling courage?

Respondent: Not like that because yesterday at the centre I was (inaudible 00:21:35) so they also the same thing, but I know that little, little, I will forget about it also.

Interviewer: So you feel confident that you’ll be able to move on from this?

Respondent: Yeah, and also sometimes if I call Ogo, when I just hear his voice I forget about vitiligo because it makes me to always feel happy, to talk to me.

Interviewer: So one of the ways that you manage your vitiligo on a day-to-day basis is knowing that you can rely on support from Ogo and from VITSAF, and what are the other ways that you manage it?

Respondent: Other way are sometimes when people are surrounding me, like sometimes I go to shop, people are there just looking at me, I’ll just forget that, what is vitiligo? I want to be with them. You understand?

Interviewer: You want to be with them?

Respondent: Yeah.

Interviewer: What do you want to be with them for?

Respondent: You don’t understand. If I went to a shop and I want to buy something people will just be looking at me, I would just put my face that maybe I’m not being looking at, I want to give myself courage that yeah, I’m living with vitiligo, it’s not a bad thing.

Interviewer: So you want to be able to have the courage to kind of say, “What are you looking at?”

Respondent: Yeah.

Interviewer: Why would you want to do that? To stop them looking?

Respondent: Yeah, to stop them looking at me, so that I will not start thinking again that this person is looking at me.

Interviewer: What are the other ways that you manage it?

Respondent: That means that I talk, you know, you meet different types of people, I talk, different types of characters, some people asking different types of questions, because in my first time in the hotel when I work, the first time when you want to start interview in the place I work people will just be looking at me. People would be talking but me myself I know that they are talking about me but I’ll just give myself courage that anyone that come down to ask me a question I will answer the person. Yeah.

Interviewer: You’ve talked also about the church and God. Does that help you manage?

Respondent: Yeah. Because people in the church that I know, people know me in the church, they know that it’s vitiligo because I’ve explained to them, so they take me as their own brother and sister, they don’t even look if I have vitiligo at all. The pastor knows about vitiligo, so.

Interviewer: Is your pastor supportive?

Respondent: My pastor is supportive of vitiligo, that means sometimes just tell me frank I don’t think about it. One day it will go. I should just move my mind from there because sometimes I complaint to my pastor that this thing don’t make me to have friends, so he said that there is a reason for everything, that I should just let it leave me alone.

Interviewer: Do you use any physical methods to cope, to manage your vitiligo?

Respondent: Drugs.

Interviewer: Drugs, yeah, or some people use camouflage or make up or some people use...

Respondent: There’s a white guest that came to my hotel from UK, so he gave me something like powder, if you look at my face that’s what I’m putting on. It’s something like powder, that’s easy to cover my face, but you will not know that I have it in my face.

Interviewer: So you’ve got the powder on now?

Respondent: Yeah, in my face. So when I (inaudible 00:25:13) you will not even know that I have vitiligo here.

Interviewer: Up on your eyes? Right, okay.

Respondent: Yeah. You will not know. But when I clean my face now you will see it there.

Interviewer: Do you use that every day?

Respondent: Not every day, sometimes I will just leave it, let me just leave my face like that.

Interviewer: Do you also use support of friends and family, does that help you manage?

Respondent: Yeah.

Interviewer: Is there anything else that you do to live with vitiligo day by day?

Respondent: Sometimes, because there is one of the seminars we went, the girls told her that we should be away from anything oily, too much of oil is not so good. Thinking, I should stop thinking and stress.

Interviewer: What was the first one, sorry?

Ogo: Oil. Too much oil. Cooking with oil.

Respondent: Like sometimes she does cook vegetable and that means we should take vegetable and eat a lot of vegetable.

Interviewer: So avoid oil?

Respondent: Yeah.

Interviewer: Avoid stress?

Respondent: Yeah, and thinkness(?).

Interviewer: Right, okay.

Respondent: Yeah. I know in Nigeria I know how the stress now, the stress is too much, so some time when I have off I use it to rest.

Interviewer: How do you manage the responses of others? So when other people respond to your vitiligo how do you manage that?

Respondent: Sometimes outside when they come to me and ask me about vitiligo, sometimes I do explain to them that look at what vitiligo is all about, so it will leave me. They got too much some time

and they’ll be asking question, question, question, question. To answer this sometimes I can’t answer, they say, “Don’t worry, one day you will not have vitiligo.”

Interviewer: Do you feel in control of how you feel about vitiligo? Do you feel you’re in control of your emotions about vitiligo?

Respondent: Some are. Some are but sometimes I do think about vitiligo and sometimes... (click of mouth)

Interviewer: I’m wondering, you've talked about the kind of things you’ve used to manage it, you’ve talked about how it’s impacted on you, I’m wondering, have you tried many treatments over the years?

Respondent: Yeah, I’ve tried many treatments. There are some drugs that they said I should use, I have been using it but I’m not the type of person that likes drugs, so I usually sometimes just leave it, I’m not too regular with the drugs, no.

Interviewer: Why is that?

Respondent: Because the drug is much... everyday everyday!. And sometimes because my job, sometimes if I’m going to work I will deal with the drugs, but for me to go downstairs and take, I should drink the drugs, would be another (inaudible 00:28:47) let me leave that, maybe later we’ll drink it then later we forget.

Interviewer: So it’s very difficult to keep taking them, and when you say they’re much, is it they’re expensive?

Respondent: Yeah, they’re very, very expensive, yes, the money is high for the drugs.

Interviewer: You spoke earlier about how your dad thought maybe it was somebody from the village that had put vitiligo onto you.

Respondent: Thought maybe somebody from the village make me to be like this. Because somebody told him that maybe I ate something that I’m not supposed to eat. So if you ask my daddy, does my fore-fore-father have this thing, he said no.

Interviewer: He said no-one had it?

Respondent: He said nobody have it in my family.

Interviewer: Has it affected your relationship with the village?

Respondent: The village? I’ve not been in the village like that, I’m busy in Lagos.

Interviewer: So you don’t go out back to the village?

Respondent: No.

Interviewer: And his wife do other people react to your vitiligo?

Respondent: Sometimes people they are good people that know about vitiligo, sometimes they’ll react very nice to me, but people that do not know about vitiligo sometimes they just talk different type of

things, behave so bad to me, but me I know that they don’t understand, that’s why they are behaving like that.

Interviewer: So does that help you kind of cope with their response, that you just think, well look they don’t understand?

Respondent: Yeah, sometimes I just look at that, if they know they would not behave like that.

Interviewer: Thank you so much for telling me about all of that, I really value all of your expertise on the subject and it’s brilliant to hear.

Respondent: Okay.

Interviewer: I’m wondering, before I finish this part, can you tell me have I missed anything? Is there anything that you think, “God, Nick should ask me this question,” and I haven’t asked? Is there anything else you want to talk about on it?

Respondent: I want to say, if in Nigeria in total, I mean I want us to bring out vitiligo, because many people have vitiligo, that means I’ve seen so many people that have vitiligo they’re going to different types of hospitals, they don’t know what is vitiligo, so I don’t know if it will bring out something like seminar or them to let Nigeria know more about vitiligo. .

Interviewer: So you think that would help?

Respondent: Yeah, that will also, because many people that do not have vitiligo, some of them are running away from us. People that have vitiligo will go like where they are looking for a job, for them to have a job sometimes is a problem, you understand? So that is why we need to let people to know more about vitiligo.

Interviewer: So if people knew more then it would be easier to have vitiligo?

Respondent: Yeah.

Interviewer: Thank you very much. I’m going to stop the recorder now.

[End of Transcript]

**Participant 6- JE**

Interviewer: James, could you please tell me, when did you first notice the changes on your skin?

Respondent: It was 1982.

Interviewer: So 31 years ago.

Respondent: Yes.

Interviewer: What did you notice?

Respondent: I noticed a white patch on my eyelid there, it’s still there, this. That is when I first noticed it.

Interviewer: Did you tell someone?

Respondent: Yeah. My mother saw it and showed it to people. They said maybe what I’ve eaten, and they said it’s a spell cast on me or whatnot. But she took me to a doctor, you know, back then the awareness of vitiligo wasn’t so pronounced. Even now most doctors don’t even want to know about it, that’s the issue, most doctors don’t know about it. Like I said at that time, a guy, calling himself a doctor, sees me and he said, “Leprosy, can’t treat it.” I was furious, I said, “It doesn’t look anything... this is not leprosy it’s vitiligo.” He was not asking me what is vitiligo.

Interviewer: So is this what happened when you first went to the doctor?

Respondent: Yes.

Interviewer: They thought it was leprosy?

Respondent: Mm-hm.

Interviewer: And you were furious?

Respondent: I was very furious.

Interviewer: How old were you?

Respondent: I was 12 then. But now I’m 45 now.

Interviewer: When you were 12 you said that the doctors didn’t really know what it was.

Respondent: No, they didn’t know.

Interviewer: So how long did it take for a diagnosis?

Respondent: It was when I was grown up around 30 to 32 years old and I went to look.

Interviewer: So that’s a long gap, it’s almost 20 years.

Respondent: Yeah, a long gap. I ignored it, I didn’t care about it. I was going about my business. It would start bothering me and I said, “Okay, let me go and check what is this.”

Interviewer: Why did it start bothering you?

Respondent: People were starting to stigmatise. That is the issue. So I said let me try and find what I can treat it with, and I went to look, they said there is nothing wrong with me, that it’s not in my blood, that is only on my skin. So I went for a series of tests which eventually they came out and said there is nothing wrong with me. They gave me some crème and they said I should go on the drug that man mentioned at that time, steroid, is it steroid? Keloid. Yeah, keloid. And the man said I should take melatonin, which eventually it spoilt my eyesight. That was too powerful, it spoilt my eyesight so I stopped taking the melatonin and keloid.

Interviewer: I’m wondering just going back to when you were 12, was it just on your eye was it?

Respondent: No, it’s all over my body now.

Interviewer: No but when you were 12?

Respondent: Yes, only on my eyelid.

Interviewer: What did you think? What were your thoughts about it?

Respondent: Frankly speaking I didn’t know what is wrong with me.

Interviewer: That’s what you thought?

Respondent: Yes, I never knew what is going on and what is wrong with me.

Interviewer: Then did you feel scared? Did you feel worried?

Respondent: Why, I should be scared or worried about what is going on. I saw this strange thing on my skin, should be scared.

Interviewer: So you were?

Respondent: I was scared, I was scared.

Interviewer: Naturally.

Respondent: Yeah, naturally I was scared. Yeah. I was scared.

Interviewer: So then there was a 20 year gap, then you got a diagnosis.

Respondent: Yes.

Interviewer: What did you call it before you had the diagnosis?

Respondent: They didn’t give it a name. No name. They told me it’s skin pigmentation, that’s all, but I’m lacking melatonin, something like that.

Interviewer: When did you hear it was vitiligo?

Respondent: Ogo made me to know about vitiligo.

Interviewer: How did that happen?

Respondent: I met her at the bus stop at Eric Moore(?).

Interviewer: So it was a chance encounter?

Respondent: Yes. I went to the bank. So it was raining. We all ran for cover and I shared bus stop. That is where I met Ogo. So I collected her number, she took my number and so we started communicating. She told me some drugs to be taking. I’ve not been taking much of the vitamins she said, I’m lacking B12, but I should be taking folic acid, B12 and vitamin C. So then she now told me it’s vitiligo. That is what happened to Michael Jackson. That is what she told me.

Interviewer: Did that help knowing that?

Respondent: Yeah. When I was taking it, it helps. I do see the white patches were now going off, but when I stopped it comes back.

Interviewer: So every day now you take...?

Respondent: Every day I take it. Now I’m using raculia(?).

Interviewer: What is that?

Respondent: It’s one of those drugs for vitiligo.

Interviewer: What does it do?

Respondent: It helps to pigment the skin. My sister sent it from the US.

Interviewer: Have you noticed any change?

Respondent: Yeah. But I started getting it just about one month ago.

Interviewer: And you’ve noticed some change in that month?

Respondent: Not really. It was the other vitamins that I noticed some changes, but this one I’ve just started taking it, I want to watch it maybe three or four months and see how long.

Interviewer: And see what happens.

Respondent: Yes, and see what happens.

Interviewer: I’m wondering if you can tell me what impact vitiligo has on your life.

Respondent: Devastating.

Interviewer: Devastating?

Respondent: Devastating.

Interviewer: Can you tell me more?

Respondent: Like I said before, it has made me to lose my self-confidence, it has stopped me from so many things that I wanted to do in life.

Interviewer: Like what?

Respondent: I’ve been playing football. So I stopped playing football because of that. I was supposed to be among the under 16 1985, I was supposed to be on it but because of vitiligo I stopped going to the training, I stopped football. Ever since then, ’85, I stopped playing football.

Interviewer: So you were a professional footballer?

Respondent: Not really professional. I was, you know, under 16, just these people coming up. I was very good, yes, so I was about making the team.

Interviewer: Why did you quit?

Respondent: I quit because I couldn't stand it now. People look at me as strange human, because I have it all over my leg but I didn’t have my hands, so I was shy putting on shorts, short sleeves and shorts. But if it is now playing football I’d been there long-sleeved and I would have played, but back then I was too shy.

Interviewer: So it stopped you pursuing your career.

Respondent: Yes, as a footballer, I stopped playing football.

Interviewer: What else has it stopped you doing?

Respondent: Well, nothing much, because now I’m a sailor and where I work people don’t discriminate.

Interviewer: What are you now?

Respondent: A sailor, I work on the ship.

Interviewer: Where do you sail?

Respondent: I sail to African countries, but I don’t go to Europe.

Interviewer: Is it shipping? Is it like container shipping? What kind of sailing?

Respondent: No, not really shipping. Sometimes we deal with this oil rig. We go to the rig, yes, where they drill oil. We take their materials to the rig and come back, that is what we do.

Interviewer: Does it impact on your job?

Respondent: No. Not at all.

Interviewer: Your colleagues?

Respondent: No. They are very free with me. I don’t have a problem.

Interviewer: What about in your day-to-day life like when you’re walking around the streets?

Respondent: That is a problem. In my area, since I don’t go out too much people in my area know me, so that it seems not to bother much.

Interviewer: Why do you not go out too much?

Respondent: I’m not an outgoing person, maybe because of vitiligo, maybe that’s why I don’t go out. But if I have any occasion I’m going maybe I just go but I don’t like, because I don’t have friends. When I go to sea and come back I stay at home with my family, I don’t go out.

Interviewer: And you have a family?

Respondent: Yeah, I have a wife and three children.

Interviewer: How old are your children?

Respondent: The first one is four, the second one is two, the third one, the boy, is just two months old.

Interviewer: Wow. Congratulations.

Respondent: Thank you.

Interviewer: One of the things I wanted to ask you about was how has vitiligo impacted on your romantic relationships?

Respondent: No, I don’t have a problem. That is one good thing about me and this vitiligo issue, I don't have a problem with women.

Interviewer: There was never any?

Respondent: Never. No, I don’t have a problem. However I do have with me, some men, but women I don’t have.

Interviewer: So even non-romantically with women do you not have a problem with it?

Respondent: Yes.

Interviewer: But with men you find...

Respondent: Some of them.

Interviewer: What can they do? Why do you have a problem?

Respondent: Some they look at you like... why I said I have a problem with me is like the other day on a bus the bus conductor refused to collect money from me. He wouldn't collect money from me. It was one other woman beside me that sat beside me that took the money and gave it to the conductor, and he now collected it.

Interviewer: How did that experience make you feel?

Respondent: I didn’t do anything but I was so furious, very, very furious.

Interviewer: So you felt angry?

Respondent: Very, very angry. I was hoping that he is going to make (inaudible 00:09:21) I was ready to punch him.

Interviewer: So you felt angry, you wanted to be violent.

Respondent: Yes.

Interviewer: What did you actually do?

Respondent: No, no.

Interviewer: Nothing? You did nothing?

Respondent: No, nothing.

Interviewer: Did you want to do something?

Respondent: Yes, but you know, I was ashamed of myself because people was near watching what was going on, so I was ashamed of myself and so angry.

Interviewer: When you say ashamed what do you mean?

Respondent: Shame is... shame or shy?

Interviewer: So you wanted to kind of shrink away?

Respondent: Shrink away, yeah.

Interviewer: how does it impact on your mood?

Respondent: Well, my mood is dampened.

Interviewer: All the time?

Respondent: They dampen my mood. Vitiligo has dampened my mood generally. It doesn’t make me snappy.

Interviewer: Do you find that you’re able to be happier, do you have happy times?

Respondent: Yeah, I do have happy times, watching TV, sitting with my family, with my friends. I do have happy times. The only time that I have a little problem is when going out because I don’t know what people will say, they look at you and they say certain things, that is when I have problem in my life.

Interviewer: So it’s other people’s reactions.

Respondent: Other people, yes. But people in my vicinity, my family, my friends, I don’t have a problem.

Interviewer: Is there any other way it’s impacted on your daily life?

Respondent: No.

Interviewer: I’m wondering how you manage vitiligo on a day-to-day basis, how do you manage it?

Respondent: Well, the issue that is, I’m trying to see how I can cope the spreading of vitiligo on my skin.

Interviewer: How you can cope with it?

Respondent: Yes. That is a problem that I face in life.

Interviewer: So you’re worried about how to stop it from spreading?

Respondent: To stop it. Yes.

Interviewer: How do you manage it as it is?

Respondent: I take my multivitamins.

Interviewer: So you take vitamins, you take medication from your sister in America.

Respondent: Yes.

Interviewer: How do you manage the reactions of others?

Respondent: Well, that’s not easy. Most people call you names. Like the other day somebody called me two colour.

Interviewer: Two colour?

Respondent: (Laughing)

Interviewer: What did you say? So you’re laughing now.

Respondent: Yeah. Sometimes you laugh about it. Call me two colour. I stood where I did, I couldn't do anything.

Interviewer: What does that mean to you, to be called two colour? How does that impact on your identity?

Respondent: Ah, no, it’s really bad, very odd, very odd statement to say?

Interviewer: Yeah. Why?

Respondent: They’re kind of facts, you know, I don’t how to put it, to tell you who you are, yes.

Interviewer: So for somebody else to tell you who you are.

Respondent: It makes me angry. Except when in a proper manner or a proper way. There’s a proper way to say it. So you can come over to me and say, “Ah, Jones, what’s wrong with you? What is this now?” And I’ll explain.

Interviewer: Do you mind when people do that?

Respondent: Yeah, I don’t mind. When they come I explain, but when you call abusive words, that is when I get furious.

Interviewer: So if people are abusive to you that makes you angry?

Respondent: Sometimes. Yes.

Interviewer: If people are courteous then you’re okay?

Respondent: Very okay.

Interviewer: I’m wondering how do you manage kind of... I think some people have talked about spiritual support has been quite important to them.

Respondent: Yes.

Interviewer: Is that important to you?

Respondent: As well, because without God nothing works. Without God nothing works so with the medication whatsoever you must put God in it, so it’s really helpful.

Interviewer: So does God help you manage vitiligo?

Respondent: Of course. If God doesn’t control your heart, you fall apart.

Interviewer: You said earlier that when you first saw the vitiligo on your eye, that there was some kind of question about spirits. Was that something that your parents or your family worried about?

Respondent: Yeah. They did not really worry about it much. It was the people around saying why is my mother not taking a step towards my vitiligo, that maybe it is a spiritual issue, maybe I have eaten what I was supposed not to eat.

Interviewer: What would that be?

Respondent: I don't know. They said I should stop eating okra. That is the problem. Any drugs(?), they said I should stop eating it. I don’t know whether you people have drugs(?) around there.

Interviewer: No, we don’t.

Respondent: But you have okra?

Interviewer: Yes.

Respondent: Any soup make with okra, they call it drug(?) soup.

Interviewer: Right, okay. Nice?

Respondent: Yeah, it’s nice.

Interviewer: So they told you to stop eating that?

Respondent: They said I should stop eating that.

Interviewer: Because it was causing the vitiligo?

Respondent: Yeah, they said, but I don’t believe that.

Interviewer: What about people saying it was a spirit, did they say it was a curse?

Respondent: I found they didn’t say it’s a curse, but they said maybe somebody cast a spell on me.

Interviewer: Who would do that?

Respondent: Yeah, this African stuff, all this jazz, British people, maybe... especially when you’re having a bad uncle in the village.

Interviewer: Bad uncle, do you mean a family uncle?

Respondent: Yes.

Interviewer: Or do you mean as in an elder male?

Respondent: Yes, elder male in the village.

Interviewer: But they don’t have to be your family, do they?

Respondent: Maybe your father’s brother, your mother’s brother or whatever.

Interviewer: So it has to be an uncle in your family, not just an older male?

Respondent: Yes. People that knows you very well.

Interviewer: So if you have a bad uncle there is a view that bad spells can be cast by the uncle?

Respondent: Yes.

Interviewer: Why would they do that?

Respondent: They don’t want your progress.

Interviewer: So they would be threatened by you?

Respondent: Yes. They would do anything to thwart your life.

Interviewer: So there are some people who believe that vitiligo was a bad spell?

Respondent: Yes.

Interviewer: What did you think about that?

Respondent: Initially I believed it because I have not seen it before.

Interviewer: Was that scary?

Respondent: It’s scary. Especially when we believe it because it was... before it came out I was very, very sick, I almost died, but when I now recovered it now came out.

Interviewer: What were you sick with?

Respondent: I was 12, I couldn't really... but I know that I was very sick by then so I was being treated. When I recovered from the sickness this came out.

Interviewer: And then there was questions about...

Respondent: Yes.

Interviewer: Was there any action taken to get rid of the bad spell? Does the village do something?

Respondent: No. We didn’t go to the village. All boys and girls have to pray for God to help.

Interviewer: And that was then was it, everyone prayed?

Respondent: Yes.

Interviewer: I’m wondering in the past what treatments you’ve tried?

Respondent: I’ve tried this melatonin.

Interviewer: How did you find it?

Respondent: It was scarce. My mother would get is it two or three jars by then, ever since we couldn't find it any more, so it was about, that’s, is it 19... get to know about meladin(?) again, started coming.

Interviewer: Again?

Respondent: Again, but I now stopped, when God told me it was no good and Ogo told me it is no good and I should be taking multivitamin.

Interviewer: So God told you it was not good and Ogo told you it was not good and you stopped?

Respondent: Yes.

Interviewer: So you've taken it a few times in the past?

Respondent: Yes.

Interviewer: Is there anything else you've tried?

Respondent: No.

Interviewer: How do you feel towards treatment? Is it something that you’re actively looking for?

Respondent: Yes. I’m looking.

Interviewer: So you want a cure?

Respondent: Yeah, I want a serious cure.

Interviewer: Do you believe there will be one?

Respondent: Sure.

Interviewer: Just a matter of time.

Respondent: Yeah, a matter of time, just like AIDS, a matter of time, there will be a cure for AIDS. Don’t you believe so?

Interviewer: I do actually, yeah, with AIDS, yeah. How do other people react to your vitiligo?

Respondent: It depends, people that know you and people that know vitiligo. People that know you, but I’ve seen you ever since birth, they will react as a normal person, but people that has not known you for a long time sees you, they tend to ask questions, that is the issue.

Interviewer: What questions, just what is it?

Respondent: How come about it? What is all this? How did you get it?

Interviewer: So they react, by either questioning, wanting to know.

Respondent: Yes.

Interviewer: Or by stigmatising you. I’m wondering, your mood, being stigmatised, has it made you feel depressed?

Respondent: Sure.

Interviewer: Has it ever been so bad that you felt suicidal?

Respondent: I felt so bad when I was in, like I said, for the mobile cafeteria, when a lady walked away from her tray, her food and left where we were sitting.

Interviewer: Because you were there?

Respondent: Because I was there.

Interviewer: So the lady got up and walked away?

Respondent: Walked away.

Interviewer: How was that for you?

Respondent: Very, very... you know, I was annoyed, and so ashamed of myself.

Interviewer: So ashamed.

Respondent: So ashamed.

Interviewer: That’s not an easy thing to feel.

Respondent: It’s not easy, in public. Living with vitiligo is not an easy task, it’s not an easy task at all.

Interviewer: What are the things that enable you to cope?

Respondent: well, if I have positive friends around me it gives me confidence. Like I’m here now, it gives me confidence.

Interviewer: So the self-help groups?

Respondent: I love the programme. Yeah, seeing other people with vitiligo gave me a lot of confidence because it looks as if I’m the only one with it.

Interviewer: So meeting other people with it really helps?

Respondent: It helps. Then it reveals other people’s talk and idea, it gives you morale.

Interviewer: Morale?

Respondent: Yeah.

Interviewer: You mentioned earlier about in the ‘80s how the doctors didn’t know what it was.

Respondent: They didn’t know, no.

Interviewer: But then you waited many years before you went to the dermatology clinic.

Respondent: Yes.

Interviewer: How did you find that experience?

Respondent: Well, it wasn’t so easy because by then I was was little and children back then that I’d play with tend to mock me. They’d call me sort of names, which maybe when I’d go home I’d tell my mum, but she said I should take (inaudible 00:21:23) that she doesn’t know how this came about.

Interviewer: Did it affect your relationships with your parents?

Respondent: No. My dad was a half British man. He lived in the UK for a long time before he came back to Nigeria.

Interviewer: What impact do you think that had, on the way he reacted?

Respondent: Well, he saw me as a normal person. In fact I am the loving(?) boy or child in their eyes, so loved by my father, and so close to my father, I’m so close to my father.

Interviewer: So now do you think that the health provision in Nigeria is good, is bad?

Respondent: Health in Nigeria, I know we are backwards. We don’t have much medicine, awareness is not there. That is the issue. If we have awareness we have drugs. Most of these drugs are imported. My sister no longer sends them to me, I don’t buy them here, some of them don’t have them here, so health in Nigeria is very, very backward and expensive.

Interviewer: So it’s expensive as well?

Respondent: Very expensive. If I can get a good health it’s expensive.

Interviewer: Thank you so much for answering all my questions.

Respondent: You’re welcome.

Interviewer: One of the last questions I have for you really is just to say, I’ve obviously written down some ideas of questions I wanted to ask you, but I’m conscious that I might have missed something. Is there anything that you think, Nick, you need to ask me this? Is there anything I’ve missed that you think is important?

Respondent: I don’t think so.

Interviewer: You feel we’ve touched on every big subject?

Respondent: Yes. Everything’s in place.

Interviewer: And my final, final question is just to say, thank you for taking part in the research.

Respondent: You’re welcome.

Interviewer: It’s really so valuable and helpful for us. I wonder if you have any feedback about the research, if you think that in future it should be a little bit different or if you like the way it was done or if you have any feedback about it.

Respondent: Yeah, I (inaudible 00:23:37). If I can have your email as well maybe if I have some words, some ideas, what I’ve experienced maybe I can send you some email.

Interviewer: My email address is here on the bottom of this.

Respondent: Okay, no problem.

Interviewer: So if you want to take that away with you.

Respondent: Yeah.

Interviewer: And then if you have any questions at all just please contact me.

Respondent: No problem.

Interviewer: Thank you very much. I’ll stop the recorder now.

Respondent: You’re welcome.

[End of Transcript]

**Participant 7- Mrs O**

Interviewer: I was wondering if you could tell me when you first noticed the changes on your skin?

Respondent 1: It was 2004, November, when I had my second baby with CS. So I only got back from the hospital and I noticed the change. And I went back to the hospital to inform the doctor. Do I accuse him that it’s a drug that he used on me that reacted, but he said no, that this one is not what he used on me. But I used antibiotics, but antibiotics cannot react like this. But that is all the risk I took in November 2004.

Interviewer: So, initially, you thought that it was something the doctor had done?

Respondent 1: Yes.

Interviewer: A mistake the doctor had made?

Respondent 1: Yes, that was my thought at first, that there was a mistake, maybe. I’d already had CS before, but that was the first one I had. I said he had made a mistake. But later he sat me down and said, “This is not the problem.” I said, “Okay.” That was all the risk.

Interviewer: And did you trust that the doctor was telling the truth?

Respondent 1: Yes, because he is our family doctor and we have been using him for the past… I’ve known him for the past six years, before that time. So I trust him, and he is an elderly person, I know that he would not lie to me, so I believe him.

Interviewer: And what did you first notice, what was the first sign?

Respondent 1: What I noticed was it started from my hand, this very place. I just noticed that this is changing. When my sister-in-law came, they all said maybe I’ve eaten something, you know, in our own culture, that maybe if you eat something that that will happen. I said, “What did I eat? I’ve not eaten in your place.” They said, “We have seen what you have eaten now.” I said, “Okay.” “What is the one that you are not eating?” they said, “No.” I said, “So what are you telling me?” But I’m not happy, because this is not the way God created me. It started, there’s nothing I can do. It cannot question God. So that is how they raised that. Somebody now said I should go to the clinic when I went to the hospital. The doctor has now referred me to (inaudible 00:02:42) and I got there. They now referred me to the skin clinic at (inaudible 00:02:48), so that is where I used to attend. They would give me drugs and they gave me Meladinine, and that thing is tough for my skin, so I stopped using it. Because if I use it, then if I stay outside, I don’t feel comfortable. And I’m not seeing they work, so that is why I stopped.

Interviewer: So I’m wanting to focus on the moment on when it first happened and you first noticed, you mentioned that you told your sister, did you say?

Respondent 1: Yes, my sister-in-law. They all thought that it was a spell on me, that maybe I ate something that is against the culture, that is affecting me.

Interviewer: When you say against the culture, what do you mean?

Respondent 1: I eat something…

Respondent 2: Some things are forbidden.

Respondent 1: Is forbidden.

Interviewer: What kind of things are forbidden?

Respondent 1: It depends on the area you come from. Like some people, they will not take palm wine. If you take palm wine, you can have something like this that will come on your body, and immediately they appeal to God everything will come back to normal.

Interviewer: So, when it first happened, people in your family thought either it was something you had eaten, something forbidden?

Respondent 1: Yes, like my husband, he came from a Royal house, so they all said maybe I ate something that they don’t eat. I said, “What is it?” They cannot even say that, because they are doing, they prepared it for them eating, because I just had a baby. And they are doing, they are preparing my food. And there is all the food that they are preparing and I am eating, so which one is forbidden? They said no, that all the food I am eating is not forbidden. I said, “So what is happening?”

Interviewer: And what did they say then?

Respondent 1: Everybody is looking for the solution. They used to go out and bring some herbs for me, and they said I should take it. I take one in the morning, they will bring in soap for me to bathe, and I should use it. And I am using everything, you know, they are all looking for a solution. Well, everything is not working. When I see that it is not working I just tell them that I am not using anything again, and they should just leave me. Anyway, I now start going to the clinic, to the skin clinic.

Interviewer: And how did your family react?

Respondent 1: Well, like my sisters, my brothers, they all feel bad, now what is this? How can it be you? I said, “We cannot question God.” Everybody is just running around to their friends for a solution, and then it’s not much, like this.

Interviewer: So it was quite a small area, was it, originally? When you said it’s not much like this, what do you mean?

Respondent 1: What I mean is that it’s not big like this day.

Interviewer: And how are you feeling during this time, when it first started and your family’s looking for answers, solutions, and what were you feeling?

Respondent 1: I feel bad, because I just think how come my skin’s all changed, that I will have to colour? I was not born like this, what happened God? I used to fast, I fast, God, where is the solution?

Interviewer: So for you one of the solutions was to pray?

Respondent 1: Yes, when I’ve used everything and I see that it is not working, we have to go to fasting and pray again that God will intervene. It’s not that God is not answering the prayer, but you know it is gradually.

Interviewer: And I’m wondering about the people talking about, did you say a spirit earlier, is that the term you used?

Respondent 2: Spiritual. The reason the attack came.

Respondent 1: You know, people think it’s an attack that somebody that is doing me… in the initial stage, yes, initially I believed that it’s somebody. There is nothing anybody can tell me that I don’t believe that this thing is natural, but I believe there is somebody behind it.

Interviewer: So you believed at the time that somebody was attacking you?

Respondent 1: Yes. At the initial stage, I believe that somebody attacked me, and there’s nothing anybody could tell me at that time, that’s how we saw it.

Interviewer: And how long did it take for you to move from thinking it was an attack to not thinking it was an attack?

Respondent 1: After three years I see that, okay, if it is evil people that are attacking me, we are now looking for a solution. They are collecting money, 30,000, 100,000 they will collect. Don’t worry, it will go. A solution. I just think one day I will pack everything in one bag and I’ll go to the dustbin and throw everything away and I’m not using everything again. Let me be. That is how God wants me to be. So when my husband came that day, “You know,” he said, “Where is all your medicine?” I said, “I’ve thrown them away.” He said, “Why?” I said, “I cannot continue like this, because everything they are bringing, 30, 50,000, everything that we are spending, it’s not working, so why am I using that? It’s no solution to the problem, so let me move forward.” Well, you know, at that time my husband, he doesn’t want me to get annoyed, because I said it is from their place that they are doing me. So…

Respondent 2: Sorry, I do have to explain that she is from another tribe, married another tribe, so this is…

Respondent 1: So I said it is from their place and he said no it can’t be that, you did not offend anybody. I said, “No.” So when that used to (inaudible 00:08:59) me that it cannot be, I said let’s wait, God will do it. But when I see his reaction and these people, everybody will come and say that I should just be praying that God will do it. We travelled to their home town to see the king. The king now said there is nobody that is doing me, that this thing just came, that he just believes that this thing will go.

Interviewer: So the king told you that it wasn’t an attack?

Respondent 1: Yes, it was not an attack, so they now bring one man from the village. That man said that there is going to be a solution to it, and he will prepare some herbs for me that I will take down to Lagos. They bring it, I use it, nothing happens. So that’s why I just say, “Well, let me wait, they will go in (inaudible 00:09:52).”

Interviewer: So could you possibly explain to me the impact that… you had your child at your husband’s tribe’s area, and then you were staying with his family, were you?

Respondent 1: My husband is from Edu (inaudible 00:10:13). But then, when we got married, I don’t have a problem with these people, but he has married from that tribe before, and that woman is still alive. That is the only telling that I have, that woman, she is the curse of everything. But then, the older sister said, “No, she is not the one, she didn’t know you, she cannot do

anything to you.” That is the telling I have. Later my sister came, they now said that I should erase that thing from my mind. That thing would always be bothering me, that if I don’t erase it from my mind, I won’t move forward. And that’s all we are doing, there is no solution. I just think that I should just wait to the Lord.

Interviewer: Can I ask, when the king told you that it wasn’t a spirit, how did you feel?

Respondent 1: I know that as a king he cannot lie to me.

Interviewer: So you trusted him?

Respondent 1: I trust him. If it is a spell, though, he would now say to my face, but I know that there is something that they would do in the village. If it is a spell they would know.

Interviewer: So when he said it’s not…?

Respondent 1: When he said this is not that anybody is doing to me. And I said, so we came back to Lagos.

Interviewer: I’m wondering whether you felt any relief?

Respondent 1: Then I was relieved, because then my mind is up. I used to feel that… I don’t believe that. Something is behind it. So when the king said, “No, nobody is doing it, I should just believe like this thing just came and it will go. But what they used to ask, is it itching me? No. Is it paining me? No. From the onset there’s something that not even scratched me for a day like this. That’s what they used to ask, “Is it paining?” I said, “No.” That’s what my husband said, he said, “Since it’s not doing anything it’s not that it has another health problem, that I should just believe they’ll go and wait. That’s what my husband used to say. And when I said, “I want to go for any conference, or any place that’s consigning this,” everybody who supports me in my house said, “Okay, you can go, no problem.”

Interviewer: And what do you call it now? Do you call it vitiligo?

Respondent 1: Vitiligo, yes.

Interviewer: And that’s the term that you use to describe it, yeah?

Respondent 1: Well, I suppose I didn’t know that it was vitiligo, until I got to the clinic. There is a guy in my office… when I now go to the clinic they tell me that it is vitiligo. I now went to the internet and I said, “I have had no worry about this thing before, this skin problem is vitiligo, you know I went to the internet to see I’ve now seen different people from different countries, that is not only in Nigeria that we are suffering the thing.

Interviewer: And how did that impact on you?

Respondent 1: Well, that made me relieved, because I’m not the only one. But then, before this I have not seen anybody with this thing before. It was now that I have it that I used to notice people on the road now that I have it. But before I don’t think I have ever seen anybody that has it.

Interviewer: So before you got it you had no awareness about it at all?

Respondent 1: I’ve not. Also, when I have this, that was when I notice people now that I see there is not only me, that I have it. Like last week, my friend in the office said, “Mrs Ashoma, just thank God that (inaudible) last week when we resumed for New Year break. She said she looked

at that man, and that man even knew that she was looking at him. He said, “Why?” she said you just have to thank God that your own is even looking. He said from that I ate like this, every way, that the only black patches that are there, its not even 1%. Said, have you seen that kind of a person I said I used to see it. He said he has noticed people like that before. She now said she looked, she looked, she looked. That guy isn’t even looking at her, “Why are you looking at me?” (Laughing). He said because he was surprised at her. “Mrs Ashoma is so lucky that when she wears make-up we don’t know… we see we don’t know that she has vitiligo.” (Inaudible 00:15:30). Not many people know about it, because people now say, “Mrs Ashoma, I saw you on TV. I don’t know, I used to look at you, but I didn’t know you had… but on that day, when I saw you on TV. I’m not around, I travelled at that time, so I didn’t know you would see it. People are calling my husband, and I saw your wife, they like her, she is good, and thank God for you that you encourage her.

Interviewer: So you were on television about vitiligo?

Respondent 1: Yes.

Interviewer: And when was that?

Respondent 1: 2011, June 25th. (Inaudible 00:16:18).

Interviewer: And how did people react to seeing you?

Respondent 1: That’s what I was saying, that people were calling me that they saw me on TV. There is another lady that has said somebody had that thing in their village, that all of them, they were even thinking that it was a spell on her. That God should forgive them. They said, “Mrs Ashoma, thank you, you should have let me know.” She did not see, she would not believe all the people. But because she’s seen me, that I am on the TV she now believes that when she goes to the village she will tell them that I said… the lady now, I did not even see her here because she has travelled now, she is not around here. She said she will go and tell them in the village there is not a spell on now, because vitiligo it’s a skin problem that can happen to anybody.

Interviewer: So did the television almost persuade people? It made them realise?

Respondent 1: Yes, it can make people realise. Like one of my directors saw it. He said, “Ashoma, I like your courage, that you can even come on here.” I said, “Me, I did not even see it.”

(Laughing)

Respondent 1: I did not see. Even my immediate family, nobody saw it. It was for people, our friend’s relatives, they did see it. Even the king, my husband (inaudible 00:17:37) he saw it. He now called my husband, “I actually saw your wife.”

Interviewer: What relationship is your husband to the king?

Respondent 1: He’s his uncle.

Interviewer: His uncle?

Respondent 1: Yes.

Interviewer: So they’re close?

Respondent 1: Very.

Interviewer: So you found that your family has been very supportive of you?

Respondent 1: Yes, everybody.

Interviewer: Friends as well?

Respondent 1: Yes. In my office, everywhere, my friends, but at first if they see me, they will ask, “Raquel (?), what happened to you?” Then I tell them that it is vitiligo, it is skin pigmentation and it can happen to anybody. And that is not contagious. That is the first thing you have to tell them, so that they will not be running away from you.

Interviewer: So that’s one of the things that you’re aware of…

Respondent 1: Yes.

Interviewer: Is that when people see it you have to say it’s not contagious?

Respondent 1: I used to tell them, because my friends, old friends, that they haven’t seen me for a long time, about ten years, 12 years, and I’m not like this. “Okay, what happened to you? Is it fire?” I say, “No, it’s not fire, it’s called vitiligo.” Some people have heard about it, some people have not. So they say, “What is vitiligo?” So people that have not heard about it, I will explain to them. But you know that if they ask you, you just need to take your time and explain to them. If not, they will just be looking at you to see if you are a (inaudible 00:19:00). So I will explain to them what happened. When I get to the office now, I will tell my… because my director, she will ask me, “What happened?” I will tell her. She said she has not noticed people like that before. Until when I tell her she didn’t know. She did not notice people. She didn’t used to look at people, even though she asked me about it, she was just being nice. She said that she is very sorry that she didn’t used to look at me. So then I think that’s like, I really thank God for that.. That’s my family, my friends, everybody, they are supportive. I really thank God for that. You know, there are some people that have this thing and their husbands run away from homes, but I thank God, my husband, he is there for me and the children, everybody. They are not running away thinking that this thing can catch them, no. Everybody they use my things, I use their things, so it really turned good for that.

Interviewer: One of the things I was going to ask was how it impacts on your life now?

Respondent 1: I’m moving forward. It’s not in any way… if I want to go anywhere I go. I go to church, and I used to shake people in the church, and they don’t run away from me, so I used to greet people. And nobody has ever rejected me, and I want to shake… nobody has ever rejected me. I’ve not experienced that.

Interviewer: You’ve never experienced anybody rejecting you?

Respondent 1: Nobody is rejecting me when I’m out. Even if I hug people, they don’t. Nobody rejects me.

Interviewer: Does it impact on when you’re, say, walking through the streets, or if you’re in the market?

Respondent 1: Well, people will look at you when you are going on the streets, but I’m not bothered.

Interviewer: You’re not bothered?

Respondent 1: I’m not bothered, because that’s the way God wants it to be, so I’m not bothered if you look at me. When I go to the market and I want to buy something, I pay. I’ve not seen anybody rejected money. Because I’ve had that think in my mind that if anybody should reject money I will take that thing and go, and I will not put the goods down.

Interviewer: So you’re prepared for that?

Respondent 1: I’ve prepared for that. You know, I had people experience, so I have that thing in my mind that if anybody I want to pay you like this. You know what? If I want to give you something you will not even, because my (inaudible 00:21:26) so I don’t think anybody will reject that. But I have not experienced that yet.

Interviewer: And what about at work, does it impact on your work?

Respondent 1: Not at all. My peers, my permanent secretary used to shake me, so I don’t have any problem with anybody in my office. My immediate boss, my director, everybody, we used to shake, when we resume on 2nd, when we go to work, everybody, we are shaking each other. So when we have been sat in a mission in prayer together, they shake me and I hug them.

Interviewer: Do you mind me asking what your work is?

Respondent 1: I work with the Ministry of Education.

Interviewer: What is your role in the Ministry of Education?

Respondent 1: I’m in the Personnel department.

Interviewer: And it’s never had any impact, you’ve never felt any stigma or prejudice or…?

Respondent 1: At all. Nobody has ever rejected me in my office, in my home, in the church, so I’ve not been rejected and I thank God for that.

Interviewer: I’m wondering whether it’s impacted on your relationships, or romantic relationship or anything like that?

Respondent 1: Like my relationship? Nobody has ever rejected me.

Interviewer: And you’ve mentioned that your husband is very supportive of you?

Respondent 1: Very, very, very supportive. He has not rejected me for one day, although I have this thing now.

Interviewer: Would it be safe to say it had no impact on your relationship with your husband?

Respondent 1: At all. But, after this vitiligo, I had a set of twins.

Interviewer: So that was after the birth of the child when it occurred?

Respondent 1: After the other one I have a set of twins. They were before this match, so at first, I even said, “I’m not going to get pregnant again because I don’t want it to affect my children.” The doctor now said, “It’s got nothing to do with your children.” And I have a set of twins, they were before this match, and it’s not affecting them in any way.

Interviewer: Do you worry it will affect them?

Respondent 1: At first I thought it would affect them, it was (inaudible 00:23:36) did say it has nothing to do with them.

Interviewer: I’m wondering how it impacts on your mood, on your emotions?

Respondent 1: Well, at times, as a woman (inaudible 00:23:53), I used to think oh God, let me come back to my normal skin as a woman.

Interviewer: So just a real wish?

Respondent 1: Well, I hope that one day it will come back. I know we are telling God what you want every day, one day to answer your prayer. So that is my wish.

Interviewer: Because one of the things I was wondering about was how you manage it. What are the things that help you cope, and it sounds from your descriptions that God is very important in that?

Respondent 1: Yes. Because you know what happened, and I used to pray, when I prayed to God, this skin is (inaudible 00:24:44) God, I don’t want it. And I don’t want it to spread more than this, Lord, come and heal me. That is my prayer point when I pray, heal this skin problem, but there is nothing you can do about it, (inaudible 00:24:59) God answers the prayer, and I would wait.

Interviewer: So prayer is a powerful aid to helping you cope?

Respondent 1: It is.

Interviewer: And what are the other things that help you manage your vitiligo?

Respondent 1: I used to eat a lot of carrots. I don’t take any drugs again, I’ve stopped drugs. You know, the one that they are giving me from the hosptial) is not working, and I used to take (inaudible 00:25:29) relief, but I don’t take that one regularly because it is very bitter.

Interviewer: So the taste is not nice?

Respondent 1: I used to eat vegetables almost every day, I used to take vegetables. And I use fresh as I used to use it to bathe.

Respondent 2: It’s NIM. It’s called NIM.

Respondent 1: So I used to use it and I take this grain seed (or green tea?).

Interviewer: And have you found any of it helps?

Respondent 1: I think they are natural, and I believe they are working. Like, you know, it’s not spreading and that is what I’m praying for, that it should not spread more than this.

Interviewer: And how long has it been stable?

Respondent 1: It’s been stable since about, like this one, when it started in 2004 it was no more than this, but later it now spread. But about three years ago…

Interviewer: So it’s been stable for about three years. And how do you manage other people’s responses? So if you see somebody responding negatively, how do you manage that?

Respondent 1: I’ve not seen people that...ah.. The only thing I’ve experience is, “What happened to you? Is it fire?” I said, “No.” You know, some people they think it’s fire, I said no. So this is the only thing I’ve experienced. When they look at me, is when I used to tell them.

Interviewer: So you used to just tell them what it was and they would be okay at that point? They would stop asking, they would stop. If you told them what it was they would stop asking you, would they? You were telling me about how the only think you’d experienced was people asking you about it and questions?

Respondent 1: No, what happened to you? Is it a fire or what? And I told them it’s not a fire, it’s vitiligo. Some people who have heard about it, they say okay, that they have heard about it. Some people who have not heard about it, I tell them that this is what happened. So in case they see another person that has even more than my own.

Interviewer: And it sounds to me as though you’ve had a very supportive reaction, so your family’s been supportive, your husband. Have your children been supportive, as well?

Respondent 1: Anyway, my children are still very small.

Interviewer: They’re very young.

Respondent 1: But then I have one that is… my first boy is in (inaudible 00:28:51) and he’s a big boy. They have not rejected me. When I go to their school, that is where you can even have names, and I go to their school, their teachers, all of them, there is nobody that has… nobody has even asked then. The only thing that my son, when I went to their school for their (inaudible 00:29:13) graduation, they said, “Your Mum is still very young and she’s beautiful.” I said, “Is that all?” I also hear from him. Maybe there is something else that they said. He said, “No, Mummy, they said that you still are very beautiful.” You know children of nowadays, they are bold. They would tell you anything. So I said, “Is that all?” He said, yes, that your Mum is still very young. Does she wear trousers?” I said, “Yes.” I asked him what did you tell them? He said, “My Mummy wears trousers, she’s working in the office, now she wears trousers.” I want to know their reaction and the friends’ reaction. So when they saw me in their school, you know, “What happened to your Mummy?” But nobody asked.

Interviewer: So it hasn’t come up. You were aware that it might, and you wanted to find out whether it had?

Respondent 1: You know, children of nowadays, they are computerised, so they can ask anything. Questions like you, you can not ask me, they are very strong willed, they will ask.

Interviewer: So they are willing to just ask anything now?

Respondent 1: They will ask you.

Interviewer: You’ve not had that experience and your son, children haven’t asked?

Respondent 1: My children, not at all.

Interviewer: And you’ve mentioned that you’ve tried a number of treatments over the years, but you’re not trying anything now. Is that right?

Respondent 1: Yes.

Interviewer: But you do use make-up, do you, to cover it?

Respondent 1: No, it’s not that I use the vitiligo make-up, no, I just wear my normal powder and my lipstick, that’s all.

Interviewer: Okay, so you don’t try and cover it up?

Respondent 1: I don’t cover it up as I used to do. But I do use my powder and my lipstick, that’s all.

Interviewer: We’ve slightly covered this, but I just want to ask in case there’s anything else for you to say on it, how do other people react to your vitiligo?

Respondent 1: Other people that have vitiligo? People who don’t have vitiligo? Most of them, you know, they don’t know what vitiligo is, unless you tell them. So their reaction is, “How can it be you?” I say, “Well, you cannot question God.” I have a friend, she was worried that what happened to you? And I told her, she said, “Okay, why you, hey? What part of the world…? How can it be you?” I said, “You cannot question God, that is how God said it is going to happen, so there is nothing I can do. The only thing that you can support me with is that you pray for me, that is all.”

Interviewer: So is other people’s prayer, other people’s support, they’re the very important things to you?

Respondent 1: Yes, it’s important.

Interviewer: Is the church important in helping you cope with vitiligo?

Respondent 1: Well, my church don’t have to do anything about it, because I go to the Catholic Church and you know that’s a very big church. I go to church, attend mass, and go. I have the society that I change it, so I don’t have any problem with anybody there, so I don’t have anything to do. I go to the church to pray and after the mass I go to the altar to pray.

Interviewer: And how much has it impacted overall on your life?

Respondent 1: About my life, it’s not changing anything. I’m living my normal life. So even though I said I should not go to it, when my office wanted to send me on a course I was there. I didn’t say, “Because of this thing I will not go.”

Interviewer: So you still go to all the same things?

Respondent 1: I am living my normal life.

Interviewer: And it doesn’t impact on your life?

Respondent 1: It’s not affecting me in any way. Because normally I go to my office, I do my work, and when they say, “You will be away somewhere,” I will go. If they want to send me out to (inaudible 00:33:49) I will go. So it’s not affecting me in any way.

Interviewer: Thank you very much for answering all those questions. I guess one of the things that I want to check is whether you think that I’ve missed anything. Is there anything I haven’t asked you that you think if you asked me this, why are you not asking me this?

Respondent 1: I think I’ve answered all the questions. The impact, how it’s affecting me, my work, my home, I think I’ve answered everything.

Interviewer: And you’ve told me about religion and you’ve told me about your…

Respondent 1: So that’s it.

Interviewer: Okay, well thank you very much.

Respondent 1: I just hope that one day God will come to our help, and let us see the research and solution to this problem.

Interviewer: So now you want a solution to come, that’s very important.

Respondent 1: It’s important. And I hope that if God should intervene, that one day there will be a solution. I know that people are trying throughout the world, that they are trying to get the research. But I know that God will do it one day, and let them say it. And our governments are good, I know one day God will touch them. And in the land that there are some people who have this thing that they will support them and release money for them to go for the research.

Interviewer: Can I ask one other thing that has come to my mind, which is how have you found the health service in Nigeria? How have you found the hospitals, the doctors, the dermatologists?

Respondent 1: They are trying their best, but you know the government is not administration. They believe that if their people should have any problem they have money, they can send them abroad. But I don’t think about other people that don’t have money to travel out to India and all these countries to look for a solution. But I pray that one day God will bring somebody that will help us in this nation, that will have the masses in their mind.

Interviewer: So you’ve found that the people you’ve met with have done their best, but that the government…?

Respondent 1: They are trying their best but I just think that they have not done much research, like people overseas are doing. That’s what I see.

Interviewer: You feel there’s not enough research in Nigeria about this?

Respondent 1: There’s not enough.

Interviewer: Do you think there is enough awareness about vitiligo?

Respondent 1: Yes, like I told you, the NTA, they showed about something we had from this, so people were aware. Now people are now knowing, are starting to know it, that something is called vitiligo. So people didn’t know it before. Even though when I get it (inaudible 00:37:07) to say vitiligo, and it’s like I’m just thinking that the doctor, it’s like when I took my secretary in the office, our secretary (inaudible 00:37:23) so we know so everything (inaudible 00:37:26) and as I now look at this I say, “So this is a skin pigmentation,” people are many outside to say you cannot explain everything to me. So, according to him, he said maybe if I go to the (inaudible 00:37:44) I will see more about it, and that’s what I did. You know, the doctors are trying, the dermatologists they are trying, but if the governments can help and pump money in so that they can go for research, I think they’re the ones who would help more.

Interviewer: You’ve mentioned that you now attend the skin clinic. How often do you go?

Respondent 1: I’m not going again.

Interviewer: So you went once, did you?

Respondent 1: No, not once, I attended it for about three years before I stopped.

Interviewer: And why did you stop?

Respondent 1: They gave me this Meladinine something, to use that to stain it, so I would go to the office in the morning. He said around 6.30, 7 o’clock in the morning that I was to… but I tell my boss that I have to do something in the morning, so I used to a little bit when the sun would come up. When I used it, it harmed my body, this was now squeezed like this. So when I went back to the clinic, I said, “This thing, I think it’s too harsh on my body.” He said maybe I applied too much, that’s what he told me. I said, “No problem,” and I just threw everything away, and I didn’t take it.

Interviewer: So at that point you just thought, “I’m done with this.”

Respondent 1: Like, already I have this medication, and I don’t want anything again that would even add to my problem. So I had to stop it.

Interviewer: Just to leave it.

Respondent 1: So when I met Ogo.

Interviewer: So then you met Ogo? Was that…?

Respondent 1: I didn’t go to anywhere again after I met Ogo. Somebody that even gave me a contact. It’s not that (inaudible 00:39:41) there is a programme in my church, at St Paul’s Catholic Church in (inaudible 00:39:48) so there is a lady that came there in 2010, and she said, “Madam, you have vitiligo.” I said, “Yes.” She said, “Have you met this person, Ogo?” I said, “No.” Okay, and the lady was working there for lots of years. She said she would record Ogo when they had some, there is a programme that they had on vitiligo. She now gave me Ogo’s number and if I call her she will give me the details. I even had the number for over the months I didn’t call her. I didn’t call her, it was easy, you know, it was (inaudible 00:28) who said, “Call her now and she will see. I now call her. Somebody from LCV, I don’t even know, if I saw the lady I wouldn’t recognise her again. The lady that gave me Ogo’s number.

Interviewer: So it was lucky, in a way, that she gave you that number.

Respondent 1: Yes, it was in ? they are recorded, because the Archbishop of Lagos came to our parish to visit, so there was a big programme. So all the TV stations came to record the programme. So that was how we met. It was on the move that we met.

Interviewer: And have you found VITSAF and Ogo’s organisation and Ogo very helpful?

Respondent 1: Yes, she is really helpful, and I pray to god that we continue to please her and help her, because she is really doing it for us. Because like, October last year, you know, we had this conference and there was a doctor that came from India. People came from different parts of the country, from the African country here. We thank God. The doctors from India, they are doing research. Ogo, she’s really trying, because you know all this natural, something, you eat carrots and a lot of vegetables. (Inaudible 00:41:56) It’s really helpful.

Interviewer: And do you find it helpful knowing other people with vitiligo?

Respondent 1: Yes, people will tell you their own experience, and what they are using.

Interviewer: So you can share experiences?

Respondent 1: Yes, you know, from other people’s experiences you can pick something. People say they used to use some drugs, but I just don’t feel like using any drugs, that’s why I’m not using, I don’t feel like. When we talk, said you are using this, using that. I also in Nigeria you have to be very careful with what you take, so that’s why I don’t use drugs. I did see the doctor, there is no drug that is recommended for me. So that’s why I am not using them. I believe in natural, you know that the carrots or the fruits, you can eat those any time, any day, anywhere. VITSAF is really helping us, because if not for that…

Interviewer: Thank you very much for answering those questions.

[End of Transcript]

**Participant 8- OM**

Interviewer: Hello, hello, hello. Okay. I think that’s working. Okay. So I was wondering if you could tell me what age you were when you first noticed vitiligo?

Respondent: 30.

Interviewer: 18?

Respondent: 30.

Interviewer: 13.

Respondent: Three zero.

Interviewer: Oh, 30.

Respondent: 30, yes.

Interviewer: Alright. Okay. And what did you notice? When did you… How did that happen? What were the first things you noticed?

Respondent: Okay. I noticed a patch here, on my inner lip. That was the first thing I noticed – and I ignored it, thinking it was one of those reactions. Then it came to my forehead, then another part. So, at that point, I felt that… Okay, no, it now came to my inner lip and was extending to this point. That was when I felt that, okay, I think something is wrong. But, initially, I felt that it was a reaction of some of the things I eat, so I had to do away with all of the things I was eating –

and then it didn’t stop. So I noticed it in February. By April, that was when I sought medical help.

Interviewer: Right. Okay. So was it then that you went to find out a diagnosis? You went to…

Respondent: That was two months later.

Interviewer: That was two months later.

Respondent: Two months later, yeah.

Interviewer: So, in that two months, it had spread from your inner lip to…

Respondent: To here, to the forehead.

Interviewer: Right. Okay. So was that when you were diagnosed with vitiligo then?

Respondent: Yes, I was diagnosed in… I couldn’t see a doctor from that April to June, so I think it was in four months that I was diagnosed – four months after I noticed it.

Interviewer: So you went, you sought out help after two months.

Respondent: Yes, but the doctor I saw was not a dermatologist. I met a general physician and she told me that it’s a fungal infection – so they prescribed some cream, which I used. It was not until June that it was diagnosed vitiligo – that was when I saw a dermatologist.

Interviewer: Right. So the general practitioner you saw was a medical doctor…

Respondent: A medical doctor, yes.

Interviewer: Okay. And then they misdiagnosed it as a fungal infection.

Respondent: Fungal infection, yes.

Interviewer: And how did you get from that misdiagnosis to then… Did you go back and say, “This can’t be right”? Or why did the diagnosis change?

Respondent: No, I didn’t go back because… I didn’t go back at that point. I still went to a laboratory to run some tests – and they still misdiagnosed. They said I… I can’t remember what they said, but they said something was wrong with me, before I saw a family doctor, who studied dermatology – a family doctor of my cousin’s, who… She just looked at it and asked me, “Do you know what vitiligo is?” And I’m, “No, I’ve not heard of that before.” She said, “Okay. Just go online and read about it.” So I have read about vitiligo online before it was diagnosed, and I was rejecting it – I didn’t want to believe this is what’s happening to me. So it was in June that I finally met with the dermatologist for a diagnosis, but I never went back to any of them to tell them it’s diagnosed.

Interviewer: Right. So you went once, they misdiagnosed, and then you never went back.

Respondent: Yeah, I never went back.

Interviewer: So the dermatologist was a friend of your cousin’s?

Respondent: No – another general practitioner that I saw, a family doctor of theirs. Because I was confused at that point, I needed someone that would explain a little of what was happening to me. So I had talked to him on the phone, but he didn’t understand what I was saying. But when we

eventually met, he actually understood what I was saying on the phone, but he didn’t want to believe vitiligo is what is happening to me. So he mentioned it and he was, “Okay, go online and read about it.” So I went online, read about it, and, “No, this is not what is happening to me.” But two days after I met him, or three days, I met with a dermatologist.

Interviewer: And how did you find the dermatologist?

Respondent: Not friendly at all because she was… She just looked at it, asked me a few questions, and just put down on the paper, vitiligo. I was trying to get her to tell me, okay, this is what you have, or something, but she never said it – I had to look into her stuff. So she scribbled vitiligo – and, “Oh, so this is it”.

Interviewer: So she didn’t tell you.

Respondent: No, she didn’t, she didn’t.

Interviewer: And why do you think that was? What was she trying to do?

Respondent: I think it’s their style or something, I don’t know – because we don’t usually ask questions in the hospital, so that’s why. So even when I was asking her, she asked me to go with the other person, asked me, get my medical history. And I asked the same person, “What is happening to me? What is it?” He told me that… (mobile ringtone) Sorry. I think it’s (inaudible 00:04:51) and I’ve got the phone. It was (00:04:54) calling and I cut it off – I’m sure she will call back. So where was I? Okay. I was asking him, and he said, “That is not my job. My job here is just to get your medical history.” So I was so worried, I said, “Okay. Who amongst you can tell me what this vitiligo thing is?” And then he asked me, “Who told you you have vitiligo?” I said, “I saw it when the dermatologist wrote it down.” “Oh” – that was it with them. The next one was

another dermatologist came in to take my picture, so I asked her why. She said so that they would follow up on me, but she’s not called me to date.

Interviewer: But how did you know about the dermatologist? Did you see it advertised? Or did you…

Respondent: No, he’s in the teaching hospital, so I… He’s in the teaching hospital. I asked where I would get a dermatologist. Because I was trying to go to the general hospital, but I couldn’t meet up – I was there three or four times. They usually take a certain number of people in the morning, and then, if you don’t get there at that time, they won’t see you.

Interviewer: So you need to get there at a certain time in order…

Respondent: At a certain time – I think before 7am or so.

Interviewer: So you found out that there was a skin clinic…

Respondent: Yes.

Interviewer: And because you knew, obviously, it was a skin… Something on your skin, so therefore you went…

Respondent: Yes, I knew that, okay, it’s a skin condition and the doctor I’m supposed to see is a dermatologist. But I couldn’t meet them there. And then the… I had to go to LUTE, but, normally, before you go to the general hospital to see any of the doctors, teaching hospitals, you have to get a referral – you have to go to another hospital who will refer you to them. So I did that and then, in June, met a dermatologist.

Interviewer: Right. Okay. And I was wondering if you could tell me which parts of your body are vitiligo present on? Where… Is it just where you can see or…

Respondent: Yeah, where you can see. It was already here, on my neck, here, on the forehead, and my scalp too. At that point here.

Interviewer: And so that was at that point, was it?

Respondent: Yes.

Interviewer: And, now, where is vitiligo present for you?

Respondent: Just here.

Interviewer: Just there. Right.

Respondent: And (inaudible 00:07:06).

Interviewer: And has it changed over the years since you first noticed it?

Respondent: Changed, how?

Interviewer: It comes and goes in different places?

Respondent: Yes. It progressed so fast. It was all here, then to this point. It progressed so fast. But when I start trying different treatments… Because I was given an injection in the hospital that reacted too badly – Kenalog injection. Kenalog reacted so badly. And when I went back for the next appointment, I shared my experience with them, and they asked me if I have anybody abroad,

so I was, “Why are you asking me that?” They said because there’s a certain medication that I can get from abroad. So I told them, “I am here in Nigeria. Give me something I can get locally.” So they talked about Meladinine – there’s a particular medicine, I told them, “Sorry, I don’t want to use that.” So they were, “Okay. That’s what we have in Nigeria.” I don’t want to use it.” No, somebody first said, “This one looks informed.” You know that kind of thing? The moment I heard that, I was, okay, this is not even the people I’m supposed to be talking with. Because I said I’m not going to use a particular medication they mentioned, so they believe that I’m informed then. If I know I know somebody, I can go ahead myself – that was the…

Interviewer: So they didn’t like you taking…

Respondent: Yeah, they didn’t like… No, they don’t usually like that – they want you to… Okay, anything they say, fine, you take it. So after everything, after all the discussions with them, they prescribed something for me again. I didn’t look at the prescription sheet when I was with them. It was when I was leaving and I looked at it and it was the same injection – after sharing my experience with it. So that was my last time of seeing a dermatologist.

Interviewer: That was the last time of seeing a dermatologist. And when…

Respondent: July 2005.

Interviewer: July 2005. And, since then…

Respondent: No, I’ve been my own doctor (laughter).

Interviewer: I was wondering, does anybody else in your family have vitiligo?

Respondent: Not my immediate family, but one of my Mum’s cousins is living with it too. I don’t know if it’s that in their family.

Interviewer: So there is… You sometimes wonder whether there is a family link, do you?

Respondent: Yes, I wonder because she’s the only one I know and they are cousins, so maybe that, I don’t know. But, outside that, I don’t know any other one, except if they had one in their forefathers or something, I don’t know.

Interviewer: So perhaps if they had it and it wasn’t visible, maybe they wouldn’t say…

Respondent: Yes.

Interviewer: Is what you’re saying. Okay. And outside of your family group, do you have any friends with vitiligo?

Respondent: Friends… I don’t know, but now they are all my friend, so I don’t know (laughter).

Interviewer: Now, yes.

Respondent: Yes, now – but then, no. No, I didn’t know anyone.

Interviewer: So at the time when you first started noticing it, you didn’t know… Did your cousin have it at the time or…

Respondent: Yes, she had it at the time. My Mum’s cousin, not my cousin. But I didn’t even know…I didn’t even remember her then. I didn’t even remember that… It was when I travelled home that

everyone else said, “Okay. This is what…” Unfortunately, we bear the same name. Everyone else said, “Okay. This is the same condition that she is living with.” But that was sensitive because they believe that she is a relative, she has tried everything and nothing has happened – it was a very… The reaction about vitiligo I got from home was… It wasn’t friendly at all.

Interviewer: It wasn’t friendly?

Respondent: No, not at all.

Interviewer: What was it then?

Respondent: Because they felt that she’s been living with this and she has tried so many things and nothing has worked. They were kind of scared. They were so worried and they believed that it must be some spiritual thing, it must be some nemesis, and we have done something – so many things that they believe. They were insisting I would have to go from one church to the other – and I wasn’t ready to do any of that. So it didn’t go down well with me and them. It was the point of view that I was trying to see how I can live with it, and they were suggesting so many things, and I was not ready to do any of the things that they were suggesting, so it turned to anger from them to me, believing that I have something that is very serious and I’m not listening to what they are saying, giving me the impression that they know more than I do and… Yeah.

Interviewer: So do you think they felt intimidated by what you were saying then?

Respondent: Did they feel intimidated?

Interviewer: Yeah.

Respondent: No, they didn’t – they were angry. They believed that I didn’t know what I was doing. They were trying to help me and I was feeling that I know too much and… But I think, after about three years, my Mum’s cousin, the other sister saw me and she was just screaming that, “Oh, my sister has to come and see you. She has to come and see you.” The impression was that I was getting my skin colour back… Even though I was getting it back for a long time, I was getting it back and losing it on my face.

Interviewer: Right. So it was coming back where it had been and then reappearing in new places?

Respondent: My face, yeah. But, for the hand, it was going and that was it – it never came back on my hand or any other place. But my face, for two years, I would get the skin colour back, I would lose it again, I would get the skin colour back, I would lose it again. But it’s been stable for two years now.

Interviewer: Right. Okay. So it first happened, you went to see the dermatologist, you spoke to members of your family – did you tell anyone else? Did you speak to your friends or…

Respondent: When I spoke to a few of my friends… I don’t really have friends, sorry about that. When I spoke to them, when I say people see me on the road and some people will cry, “What is happening? Somebody must have done this to you”… They all believed that it was some… Maybe I’ll say black magic. I don’t know what you call it. We believe so many things in Africa – somebody must have done it to me. So when I meet somebody I have not seen in a while, the reaction was always something I didn’t like. Always some people end up crying, “Who did this to you?” and all of that. So what I learned at that point, if I know I’m going to see anybody I haven’t seen in a long time, I’ll prepare the person’s mind, say, “Something happened to me. I don’t want you to go crying or to go giving me any kind of impression. When you see me, we will talk normally and you will go.” If we agree on that, then we will meet. If we don’t agree on that, I don’t meet anybody.

Interviewer: Right. So you always warn people then?

Respondent: Yes, before… Yeah, because it was affecting me so much. My mother was always… She over-stated. She couldn’t just understand it. I would sit down and she would come, “Go white, go white, I seen it.” She was just so uncomfortable with it. She would go…Lagos is in the East. She would come here sometimes, “I’m sure you’re covering it. Please, do cover it all the time.” Then my father will call me every day, reminding me that there is somebody that did it to me, that I should go and confront the person.

Interviewer: Your father?

Respondent: Yes, yes. It was a very serious case then because I had to, at some point, call… No, at some point, I had to tell him, “If you believe that somebody did it to me, you are my father, confront the person on my behalf. But, for me, I don’t believe that anybody did this to me – this is a medical condition.”

Interviewer: So how long did your father take that position?

Respondent: It was about… It runs into months. I can’t remember now, but I know it was more than six months – until, one day, I just decided I won’t take his call anymore, I won’t call him. So I didn’t call him for six months. We did not speak on phone. We did not talk on phone. He now tried to get to me through my siblings, and I told them to tell him that I am not ready to speak with him until he will allow me be, that I am trying to manage this condition and he will call me every day, because I end up speaking with him and I will be crying. So, at that point, I was just

looking for (inaudible 00:14:45). I wanted to get everything off me, to see how I can focus and manage, how I can live with this and how I can go out. Because it got to the point I wasn’t even going out. I get to the door and they see vitiligo, they speak to me, they will start staring again, people will start talking again – because they were making nasty comments and… So I was just looking for a way then to continue with life, and he would call me and tell me that all the time, so I stopped taking his call – for six months, I didn’t take his call. So, eventually, when we spoke on the phone, it was a different him – it stopped.

Interviewer: Right. And he had accepted…

Respondent: Well, I don’t think he’d accepted it, at that point, because when I had started talking with people living with vitiligo and all, I was on my way to Abuja some day, by road, and he called me and asked me, “Where are you?” I said, “I am on my way to Abuja.” And he asked me, “To do what?” I said, “I am going to speak to some people about vitiligo.” He was so angry. On the phone, he was, “You must get off the bus and you must go back to Lagos. Get off that bus and go back to Lagos.” Okay, I turned off the phone. I didn’t speak with him all through the journey. I finished what I was doing in Abuja and I came back. I think when he accepted it for the first time was when I had a programme on TV. So people would now call him, “I saw your daughter. I saw your daughter.” So he now called me, “Do you mean you are actually doing this?” I said, “Yes.” He said, “Okay.” He gave me his blessings. He was, “Okay. If this is what you’ve decided to do, great, go on with that.” That was when he accepted it, but it took, I think, 3/4 years.

Interviewer: I’m wondering – because I know you use the word vitiligo to describe it – when you’re talking to other people, do you call it vitiligo or do you use a different word or…

Respondent: Okay. I usually tell them, “Okay, this is vitiligo.” But I find they don’t understand when you say vitiligo. I have the name and the definition that I use – a skin condition that turns black man white and white man whiter. That’s what I just say – and they understand it that way.

Interviewer: So you use that description.

Respondent: Yes.

Interviewer: But do you then call it vitiligo as well or…

Respondent: Firstly, “This skin condition is called vitiligo.” “What is that? “What does that mean?” “A pigmentary disorder.” They still don’t get that. I will now say, “Okay, it’s a skin condition that turns a black man white and a white man whiter.” They’re like, “Oh, okay. So how can a black…” They usually ask me how can a white man turn whiter. If I have pictures, I will show them. “Can you see this?” Usually, they notice it more when they suntan and all of that.

Interviewer: And how do people react when you give them that description?

Respondent: Initially, nobody believed me. I will give you a case that happened. I was in… Somewhere on the island, with a friend. Now these two ladies sitting opposite us were looking at me – then it was still so much on me. (inaudible 00:17:23), so they were asking my friend, “What happened to her?” What’s that?” She was telling them there is one skin condition that just started happening to her. And my friend, who I had known way back from school, so she knew me when I didn’t have vitiligo. And when I came back, they were laughing. They were, “So she gave you that and you believe her?” The two ladies were, “We have travelled far and wide, and you are just telling us this and you want us to believe it.” So, when I came, my friend was really angry with them. She was, “I am telling you what happened her. You are telling me that is not true.” So, when I came, she now told me, “See what they are saying.” So I looked up to

them. They were, “Give us the gist” – that kind of thing. “Give us the gist. Come, tell us.” (inaudible 00:18:07) So I was, “Okay. What do they think happened to me?” They told me that I used some cream – that I wanted to bleach my skin to white and it now reacted. They told me to accept it and tell the truth – I’m now claiming that something happened to me. Then, other people, when I use, “Okay, this is the skin condition Michael Jackson had.” “Oh, spare me that, please. Are you trying to find some thing with Michael Jackson.” Initially, it was difficult for people to understand. They didn’t… Nobody even wanted to listen to what I was saying, at the initial time, apart from people living with vitiligo, and their families – because they were just, “Okay. Finally, there is somebody with this symptom that I have that is even saying something about it.” They were all eager to listen then. But for every other place, uh-uh. But after about a year or so, it started changing, and now they believe you. Because, now, anybody that sees any small dots on their body, they are calling me up with, “I saw something. Please, I need to see you. I want to know if this is vitiligo or not.”

Interviewer: So people now treat you as the expert on it and will come to you and ask?

Respondent: Yes, yes. Well, funnily enough, they will come to me before they go to the dermatologist, but I always refer them – “Go and get diagnosed first – then, come, let’s talk.”

Interviewer: Okay. So you think that diagnosis is important?

Respondent: It is important because you don’t just… Even when I look at it, even when I know it’s vitiligo now, I will ask them to go to a dermatologist, get diagnosed, then come.

Interviewer: And why is that?

Respondent: I’m not a medical doctor. And I wouldn’t want a situation where somebody says… Okay, I told you it is vitiligo, and then because of that… You know? And some people tell you some things

on the phone, and I tell them, “Sorry, I can’t tell you anything until I see you.” But the first question I usually ask them, when they come in for the first time, is, “Which doctor have you seen?” And they’re, “I’ve gone to so-so doctor. I’ve gone to so-so doctor” – if I talk with them and find out that it is not a dermatologist, I will advise them, “You need to see a dermatologist first.” I’m trying to view it as… I don’t know how to put it. I think it’s a medical doctor that’s supposed to diagnose any condition, not just anybody, so that’s it.

Interviewer: So you think that’s the role of a medical doctor, therefore it’s important that’s done…

Respondent: Yes.

Interviewer: And then…

Respondent: Yes. Then we can now discuss and all that, yes.

Interviewer: And what impact does vitiligo have on your daily life now?

Respondent: Oh, there’s one thing I say every day – I’m forever grateful to God for vitiligo. Because it has changed my perception completely about life and I understand empathy. Before now, I think I was a typical Nigerian pity-pity – we just say it and we pass, we don’t really think, okay, what’s this…

Interviewer: PTPT?

Respondent: Pity, yeah. You know, we just pity.

Interviewer: Oh, pity.

Respondent: We are fond of, “Aya.” We do that a lot – aya, sorry, and it ends there, they don’t do anything. But, now, I see not just people with vitiligo now, I see people with different conditions and I put so many things, I think about so many things, what they are going through in this, in that – so many things. And it has made me become a more confident person. There’s no place I want to go now that I don’t go to. If I want to see the president of Nigeria, I will organise how can I see him. It’s not… I don’t do, “Okay, tell me how I can…” Start calling somebody – “Please, how can I…” Because I found out that the people you are calling sometimes don’t really have what you need – they are just carrying themselves because they find themselves in certain positions. It has made me understand people more.

Interviewer: So that’s very… It seems like a very positive description.

Respondent: Yes. Now, I know. But when it happened, I didn’t know it was going to be something positive. I was depressed, I was traumatised – I was everything negative. It was so difficult for me at the initial stage.

Interviewer: So it’s changed from being a very negative experience to becoming quite a positive one, where you’re feeling more confident and…

Respondent: Yes.

Interviewer: And you mentioned that you now have greater empathy…

Respondent: Yes.

Interviewer: Whereas you said typical Nigerians just have pity.

Respondent: Pity – lip pity. Call it lip pity – they just say it. “Oh, sorry, that…” It ends there. They don’t think about it. They don’t even… You know, it just ends there.

Interviewer: Yeah. And why is that? I don’t…

Respondent: I don’t know, I think it’s…

Interviewer: Explain that to me.

Respondent: I don’t know, I think it’s our society. When they want to help, Nigerians help a lot too. But it’s when you put it in their face and try to… “This is why you need to help this person. This is why you need to help this person.” Things are changing now because people are… You see Nigerians now, I’m telling you that anything that happened to anybody, anytime, it was not like this before – our psyche is changing a lot. Most of them, it’s like that because – like I mentioned last night – everything about us is too religious. There will be some beliefs that certain things happen to you because you don’t know how to pray, certain things happen to you because you are not going to so-so church, so-so pastor is not your pastor – if you come, my pastor prays for you. That is why they keep inviting you to come – my pastor prays for you, everything will be fine. And I keep wondering if it is the same God we are praying to, why does it matter which pastor prays for you? And anything we do not understand in Nigeria is a mystery. I started talking about vitiligo – because they don’t know what vitiligo is, as far as they are concerned, it’s not normal. So things are changing now because people are beginning to open up. And another problem we have here is that we don’t share our stories. We are always worried about what will they think of me after sharing that story – the stigma, the da-da-da. So they will put the story… They paint it in a certain way that the society will accept it before they tell you that. So I know so many people who have talked to me will go, “Wow, from what you have said, I have learnt a lot. Now I understand that. Now I understand that.” Because anything you ask me, if I want to, any part of the story you ask me, I’ll tell you and I walk away

– it doesn’t matter what you think of me and the story that I just told you. I have just said to you, “This is it.” But I really believe the person with… It might be something tomorrow, it might not be vitiligo – it might be something worse. So it gets people thinking.

Interviewer: So would you say, overall then, it’s… Have I understood that it’s made you a more honest person in your interactions with people?

Respondent: I’ve been a very honest person from… I’ve been, from day one, a very blunt person, though I’m learning how to be diplomatic now because I say some things now and they’re, “Why do you have to say that?” “Because of the work.” You understand? Yeah, but a more honest person in the sense that it doesn’t bother me what you think about me… Sorry. About me sharing exactly what I am going through, with you. It doesn’t bother me. I feel that sharing it with you will make you understand more and consider someone – maybe your colleague or a friend’s colleague or something.

Interviewer: Because, now, you’re full-time working at the Vitiligo Society…

Respondent: Yes.

Interviewer: In previous… Well, in that work, how does it impact you, having vitiligo, in your current role?

Respondent: I don’t understand.

Interviewer: So I’m wondering how… It’s an interesting one because having vitiligo, I’m wondering how it impacts on work and your working day, but then I imagine, for you, because your work is around vitiligo, what impact you having it has on your work in the Society? Do you think that… Does that make sense?

Respondent: Explain to say how has it… When has it impacted… You mean… Is it how am I accepted in the society or how my everyday job goes?

Interviewer: How your everyday job goes as a result of your vitiligo? Does it impact on your day-to-day life in your job?

Respondent: Oh, well, yes – yes. Yes, because if it was around vitiligo… Working with… Firstly that, working with vitiligo is the first job that has given my 100% fulfilment. Because it gives me joy to see somebody say, “Wow, I don’t need to be that scared now.” (Inaudible 00:26:29) told me… She will be coming in tomorrow. Told me that, “You told me that I’m beautiful. You told me that I didn’t bring vitiligo upon myself, so why worry about it.” She called me and she was telling me that – so I am a happy person now. When I hear things like that, I want to do more. Do you understand?

Interviewer: So it motivates you.

Respondent: Yes, yes, that’s what motivates me, but I want to do more. Then, when I hear the very negative part too, I want to do more. The very negative part has paused me, tells me that you haven’t started – there’s a whole lot of work that needs to be done. Because, what I went through, I don’t wish anybody 1% of it – I don’t just wish anybody. Though, talking with people, I notice that people have experienced worse, worse, worse, worse, worse, worse experience than me. But even the one that I had… Because I know that it affected me – it most affected me mentally and all of that. But I look at people… I’m just looking for a way to change this, and I want this person to be happy, I want you to know that it doesn’t remove anything from whom you are and all of that.

Interviewer: I’m wondering, in previous jobs, how has vitiligo impacted on your work?

Respondent: Oh, I had to stop working because I couldn’t really perform. I remember my last job, it was a sales job – sales of a magazine – and I was a sales executive in (inaudible 00:27:58). I had vitiligo when I got the job and it was so… It was much. But when I get to a client to talk to you, the first thing I try to do is to tell you that I wasn’t born this way, this is not me, instead of selling the product that I came to sell. So it affected me so much. I couldn’t really perform. If only in the office – because it was a very big office – everyone in the office, the colleagues, we are all friendly, I know, but, within me, I would be thinking that they are just patronising me, they are just saying it so that we will all get by and… So it affected me and I had to stop working. Even when we were advised to resign because of our performance – me and one other person… She didn’t have vitiligo but she didn’t perform too. When they asked us to stop working, I pleaded that they give me a position in the office, though I am not an office person, I’m more of a field person. I pleaded that they give me, but they said no. So it was after that, it was after I left the job… No, that wasn’t even my last job. I got another job, but that wasn’t serious – I was just going there just to leave the house. The magazine one, it was after I left the job, I just went there on a visit and I saw the editor, and I’m sure she was just trying to get to know what are you doing now and all of that. I said, “Well, I….” Because I was working with African fabrics – I used to design African fabric, but I had to stop too because the chemical was affecting me – the vitiligo. So she just asked me, “Okay. So what now?” And I said, “Well, I try to talk with people that have vitiligo.” And she was, “Vitiligo?” I said, “Yes, that’s what I have.” And she asked me into her office and sat me down before she said… She apologised. She said, “I’m so sorry I was a party to the team that said you have to resign. I never knew you had these huge challenges.” So I looked at her and said, “But it is on my face.” She said, “I didn’t know.” She didn’t see that vitiligo. She was not aware, that I was so confident – but, as far as I’m concerned, I didn’t have 1% confidence at that point. But she said I carried my job, I did my job, as in when I’m around the office that I was so confident. So she now asked me, “Okay. Do you want me to do an article on it?” So that is actually where it all started – because it was (inaudible 00:30:13) magazine. Now they’ve moved it (inaudible 00:30:15) Nigeria. It was a widely read magazine – women’s magazine, actually. Now we did an article. It took us one year to get the article published. When she talked to the dermatologist, she wanted to get practical day-to-day… Their day-to-day experience with the patients, but they were all giving her textbook stuff. So she told them that if she wants to get it from the textbook or from the internet, she will go and do it that way – she wants them to tell her their experience with… But she couldn’t get that for a year, so she eventually worked with what I told her and what she saw people… Online. (Inaudible 00:30:49)

Interviewer: So it sounds, in a way then, it affected your job in two ways…

Respondent: Yes, it affected it…

Interviewer: Psychologically, in that… Three ways. Psychologically, it affected your confidence.

Respondent: Yes.

Interviewer: Then it also affected the way that other people treated you – even if they didn’t show it to your face, they would have treated you differently.

Respondent: Yeah.

Interviewer: And also you mentioned that you couldn’t use certain chemicals because it was affecting you.

Respondent: Yeah, that was when I had stopped working and I went into business – my own business. Doing the fabric, I had to dye them. And it was going well, but, at some point then… I noticed that for… I think for about 8 months to one year, I didn’t get a dot of re-pigmentation, so I was

wondering, I’m supposed to be re-pigmenting. So I decided, okay, let me stay off this chemical for 6 months – and (inaudible 00:31:42) hand re-pigmented after that.

Interviewer: Really?

Respondent: Yeah.

Interviewer: So you noticed a very big difference.

Respondent: Yeah, a very big difference. And that was how it continued to re-pigment, so I stayed off that. I tried to work with people, but it wasn’t easy.

Interviewer: Not easy. Okay. And I’m wondering how do you manage vitiligo in your life now on a day-to-day basis?

Respondent: It’s not an issue for me now. I’m actually wondering, okay, eventually, the one on my face is going to go, I’m going to get my skin colour fully back, and I’m wondering, okay, how am I going to look, what am I going to be like, how’s it going to be with people. It’s not… It doesn’t affect me anymore. I don’t even see it. I only remember it maybe when I talk with somebody or somebody says, “Oh, this is going”, so I look in the mirror. I look in the mirror every day, but I don’t see it. I’ve gone way beyond that. I don’t even remember it anymore. But it’s just like when you have a birthmark, it’s something that is part of you – that’s what I see. But, before now, I was believing that, okay, I want it to completely go. Now that it’s going, I’m thinking, okay, if it eventually goes, what is it going to be like.

Interviewer: Yeah, so it sounds a little bit like you have to consider your identity and think, “Well, hang on, if it’s not there, who am I?”

Respondent: Yeah. I usually say it’s my fashion statement. Because people react when I tell them, “Okay. You go and pay so much money to get this tattoo done. This is my white tattoo” and all of that.

Interviewer: So is that one of the ways you manage other people’s responses, is to almost say it’s who I am, it’s my statement?

Respondent: Okay. When people talk to me about that… Funnily enough, I don’t get any negative… I hardly get… I still get… Especially when I go into a new environment now, but not like before. Even when I get negative talk, I just laugh. And I’m always like, if you know where I’m coming from, you won’t even be saying what you are saying. But what has worked – which I tell people a lot – is self-acceptance. And let people understand… I try to make them understand, which they are beginning to get gradually, that what you give people is what they take. If you present yourself a rejected person, they don’t have any option. Because I had a situation… There’s one man called James. I met him on the road, and I just walked up to him, “Hello. You seem to have the same condition…” – the kind of embarrassment, he almost hits me. So I had to run. I was, “Oh, I’m so sorry. Please, I’m sorry, I thought we had the same condition.” Fortunately for me, it’s (inaudible 00:34:27). So I ran into a shop – and he ran into the same shop. So I just (inaudible 00:34:34) this guy can’t hit me here. But I didn’t know he was regretting what he did. So he now came up to me and apologised that he’s so sorry about that but because of people’s reaction, what people have told him, that’s why he got… And he didn’t want… He doesn’t like people saying anything about his vitiligo. So I said, “But I have the same condition. Can’t you see I have the same thing? I expected you to… Okay. She has the same condition. She’s trying to talk to me.” He apologised – but that’s all words. I met him again and he was so negative, so… He was so negative that I didn’t want to see him again. So I now started thinking of how people outside perceive us. In other words, one of the things that made me to start thinking about that, okay, look at this guy, I want to talk with him but his attitude, he just presented more rejected (inaudible 00:35:26), just like he has given up on life – and I didn’t like that. I was just telling him, “Why are you like this?” He said, “I have vitiligo. You’re

supposed to understand why I’m like this.” I don’t know, but this is too much. So, afterwards, he called me on the phone, so I gave him excuses. I didn’t want to see him because, again, that day I saw him, he felt that, okay, because she has vitiligo, I have to date her. So I told him… When he was calling me on the phone, I told him, “Sorry, but I have the same condition, I have the same condition – it doesn’t mean we must date. I am not interested in dating you or anybody. I just wanted to talk to you about vitiligo.” And he went, “Okay. You have vitiligo and you are rejecting me, you won’t even date me” and all these other kind of things. So that informed me more on how what we present to people and how they think of us. I learnt a lot from that.

Interviewer: Yeah. I think that’s a fascinating example. So, really, if you present to somebody as very down and rejecting, then they will…

Respondent: They will treat you that way.

Interviewer: Treat you the same way.

Respondent: That’s how they will take you. Because I started thinking much about it later. I said, okay, he has vitiligo, I’m supposed to be interested in talking with him, but this guy, he’s too negative, he’s too this, he’s too that. So I started connecting it to the way… Just somebody, if you’re downcast, you are this, you are that, the person won’t want to stay home with you. So it has helped me. I enter anywhere. I make noise a lot of times. I am always cracking jokes. I’ll go here, there, and da-da-da. People don’t even get to see the vitiligo first. It’s much later, when I ask them, “I’ve been trying to meet you. Tell me more about this vitiligo.” That’s what I get now. Not like I enter a place… There’s another thing I’m enjoying too. You enter a place and all heads turn. “Who is she?” Somebody knows that lady has been talking about vitiligo. So it’s one of the things I tell them too – give people more to look at. Carry yourself well, find

something nice and put on, so that, when they are looking at you, you tell your subconscious, “Okay. They are either admiring my dress or they’re admiring my hairdo or something.” Don’t tell yourself they are looking at vitiligo.

Interviewer: So the psychology is very important…

Respondent: Yes.

Interviewer: And how you manage people’s responses. So if you do it confidently and you go out there with a can-do attitude, then that really helps.

Respondent: Yes. Saying it is very important, it is not that it is very important – it is the thing. Managing it psychologically is just the thing – if you can get that sorted, then you’re fine.

Interviewer: And do you manage it with any physical treatment or…

Respondent: Manage my vitiligo?

Interviewer: Do you use any make-ups or anything? Or do you just stay psychological?

Respondent: No, I don’t use any make-up. I don’t cover it with make-up. I’m fine the way it is. It was not until… Even in the past, I had tried some topicals, but I’m lazy with topicals – I hardly even remember to rub body cream. To come and start putting this here, putting that here… I had tried so many paths, but I don’t think I used any for more than one month – any of the creams for more than one month. I’ve always believed in sorting it out from the inside – getting it corrected from the inside.

Interviewer: And when you say the inside, you mean from within yourself?

Respondent: Yeah, from within myself. Because I talk so much of positive attitude, I learnt that, even when you take all the medication and you worry too much, the medication doesn’t work. I learnt that you have to put your body in the position to heal itself for it to heal itself. I learnt all of that. So (inaudible 00:38:54). Like I said this morning, I’ve had all my green juicing and all of that. Those are the things I eat. I eat my raw foods and all of that. And I’m happy – I try not to allow anything to get to me.

Interviewer: So is that one of the things then that you do, is to make sure your diet is very good?

Respondent: Yes.

Interviewer: So what kind of things… Is that for the vitiligo or just generally?

Respondent: Well, I started doing it for the vitiligo. Initially, when I learnt about it… They have all these Indian… Talking about diet online. And I got involved with all of that. And, at the end of the day, I found that this is not true. I was a vegetarian for 12 months. I was a vegan for 18 months. But what I said found out is not true is that they say don’t eat tomatoes, don’t take orange, don’t take that… So many, they said don’t take that. These are the things we need, I found out later. So I have been into all the trial and error, trial and error, trial and error, and I’ve gotten to a point now where I know that I need all the fruits, I need all the vegetables, especially the dark green ones. So I don’t even wait for somebody to tell me, “Okay, it’s supposed to be this.” Any vegetable I have in the morning, I throw it in the blender – I don’t have a juicer. I blend it, sieve it and I drink.

Interviewer: So lots of fruit and vegetables.

Respondent: Yes.

Interviewer: Just as part of a good, everyday diet?

Respondent: A good everyday diet. And I found out that it’s helped me for vitiligo because they say it’s an autoimmune disorder, it’s helping to fight the free radicals that causes the… And the antioxidants and stuff. And, again, I tried this severe one – very severe one. (inaudible 00:40:24) And it was a serious thing. I started studying about that. I found out that it said, sometimes, when you have one autoimmune disorder, you have another one. So I was linking it to could it be… Okay, because vitiligo is an autoimmune disorder, is that why I am having this? And it was not diagnosed until… It started in 2007. It was not diagnosed until 2010. I didn’t even know what was wrong with me. I couldn’t climb a staircase. It was very… I couldn’t climb a staircase, I have to stop, I have to hold something. I was searching for how can I at this stage… Doing all of that. So I became… I intensified my diet and I’ve not taken a tablet – and I’m good. So it’s working for me and I’m doing more of that and I’m telling everybody, if you want to… I tell them don’t go to (inaudible 00:41:10), stop wasting money – eat vegetables and you’ll be fine.

Interviewer: Eat well and… And you’ve mentioned a couple of times that spiritual… The church is very important…

Respondent: Yes.

Interviewer: And spirituality. Does that help you manage vitiligo?

Respondent: Does it help? I don’t really think so. Because they have given everybody this mindset that, okay, there is this (inaudible 00:41:33) here, whose report do you believe? The report of the Lord, not that, not that, which is very fine, I trust God, I am a very good Christian and all of

that, but there’s something I usually say – “Go to the doctor and trust God that the medication will work.” Do you understand?

Interviewer: I do, yeah.

Respondent: Because most of them don’t even know what vitiligo is. And they make you… They tell you that don’t worry, don’t worry about the doctors, don’t worry about this, don’t worry about that. Your fate is not my fate. I don’t know what you do. I don’t know whether you spend all your night praying to God in the night, crying and waiting for God to do something. There are certain things that we don’t know. I met a girl in church. Hers was… I don’t know, it was here. And I walked up to her like, “Hello. How are you?” da-da-da. We talked. I said, “We have the same condition.” She said no, hers is an attack – a spiritual attack. I said, “Okay. Maybe it is, but I’m trying to make you understand that is a skin condition.” She said, “No, it’s not the same thing” – her own is different. I said, “This happens to people even from outside Nigeria, even from outside Africa.” She said, “Hmm, but my own is an attack.” She has been… Her mind says… She has been so brainwashed that she believes that this is an attack. She’s not ready to do any other thing. So I asked her, “Okay. Are you doing anything? Is anything helping you?” She says she is using olive oil and that it is going. Her sister said, “It is not going. It’s there. It’s coming out more.”

Interviewer: And she believes it’s an attack…

Respondent: She believes it’s a spiritual attack and that the only place she can get her healing is in the church. There is nothing wrong in believing, trusting in God to heal you, but when you are believing it is a spiritual attack, the belief is contradicting. You understand? So sometimes I say the pastors have taken… Assumed the position of a psychiatrist and a psychologist, even when they don’t really know the processes. If you’re counselling someone, send the person to

the right place to go to. Tell the person to go and meet (inaudible 00:43:28) person. I don’t know if you get what I’m saying. So I was given an opportunity to talk in my church, and I talked and my pastor was, “Wow.” He never saw this, this way, that this is true sometimes, what they think they know, they actually don’t know. He’s a very liberal-minded person that believes that you must see your doctor, you must check your pee-pee, you must exercise – he’s that kind of person. The majority of them are not like that. There’s a case of one man, (inaudible 00:43:56) – he’s not in Lagos now, he’s in (inaudible 00:43:59). In 6 months that his vitiligo started… You need to see him. He was taken to about three churches in the East (inaudible 00:44:06), and they gave him one… I don’t know what they gave him that he took. He so believes… He sits here, he will be… Always worry. I was, “What happened to you?” He said he doesn’t know. He doesn’t know, that they took him somewhere, that he hopes that he gets better some day. In the space of that 6 months, it has become almost all over him completely. I don’t know what they gave him that (inaudible 00:44:26) vitiligo even more.

Interviewer: But people have such faith in the church that they put their faith in their pastor above faith in medicine? Is that what you’re… Have I understood that right?

Respondent: Yeah, they but their faith in pastor, not even God.

Interviewer: But in the actual pastor who they have the relationship with – they trust everything that that person…

Respondent: Yeah, everything that that person is saying. I’m not saying it’s not a good thing to trust God. Not to trust a pastor – because a pastor is a fellow man. I’m saying it’s a good thing to trust God, but do certain things that you’re supposed to do. Why trust in God (inaudible 00:45:01).

I saw a small boy in September in a school, and I called him, “How are you?” da-da-da. We talked – and he would not even let me say anything about vitiligo when he told me, “Sorry, Aunty. What you have is not what I have. My own is different. God knows why it happened to

me. I don’t want to discuss it.” He was about 8 or 10. That is what the Mum has told him. So I now said, “Okay. Can you give your Mum this? Do you know that there are certain things you can do that might help you?” “Nothing can help me.” The boy told me plain.

Interviewer: And that’s a response that you see time and time again, is it?

Respondent: Yes. Because…. (Mobile ringtone) Sorry. So these are all the church, whatever. I don’t have anything against them, but I have said… I’ve said it in my articles, I’ve said it even on TV, “Pastor, be a pastor. Leave a psychologist to be a psychologist, leave a psychiatrist to be a psychiatrist. If you want to be a psychologist, go and study that and add it to your pasturing. Stop misleading people.”

Interviewer: So you think that’s something that you, personally, have championed against, is the idea that…

Respondent: Yes.

Interviewer: And you mentioned that you don’t use any treatments now, but in the past, you have trialed a few but you’ve not found them to be effective or you’ve not…

Respondent: There was a treatment I used… I used it for 6 months. It’s called Vitix. It didn’t do anything. I started that because there’s a phrase or something – they usually say if you try a medication between 3 and 6 months and you don’t see any improvement, move to another one and try another one. I tried Vitix and I didn’t see any difference. One that I’ve used that I’ve seen so much improvement with is called Recouleur. They said it’s a French word – it means getting your colour back. And it simply contains vitamin B12, which I know that we need so much, and folic acid, and I think magnesium (inaudible 00:47:23). (Inaudible 00:47:24) is a supplement. I was on it for long and I saw so much improvement with that. But I’ve not been on it for a while, but what I do now, I just buy vitamin B12 and take the normal 2000mcg with folic acid in a day.

Interviewer: As part of your healthy diet?

Respondent: Yes, yes. So that’s what I do. But, basically, for me, the key thing is be positive, put your body in a position to heal itself, do your plant-based diet, and it will be fine.

Interviewer: And soldier on (laughter). And my final question, really, and I know we’ve touched on this, is really just, overall, how do people react to your vitiligo in Nigeria?

Respondent: Now?

Interviewer: Mmm.

Respondent: Well, how do they react now… In some places, now, I enter, they’re like, “Hey, she’s here.” I don’t know if they know my name (laughs) – I’m just kidding – but it’s just like… I think they see… The reaction is not so much about my vitiligo anymore but how I have persistently struggled to get this seen. So what they usually say now is, okay, that which you think is a problem can actually be a blessing – something like that. Because, most places, I usually tell them look at how you turn something, that is supposed to keep you down, into something very… That kind of thing. So sometimes, when I get to speak to people now, I try to make them understand that that’s in your feeling that’s keeping you down, but, actually, it can be something that can give you fulfilment in life. But the reaction to my vitiligo, for now, is very positive because I don’t even see you if you are negative – that’s the truth. I don’t even see you.

Interviewer: So if people are negative, you’ve stopped seeing them, you just don’t bother?

Respondent: No, if… The only people I see and I give a chance in life is people with vitiligo or some other conditions that is putting them down. But for somebody that feels that I am (inaudible 00:49:26), I don’t give negative people a chance, no – it would be a waste of time.

Interviewer: Okay. Thank you very much.

Respondent: You’re welcome.

Interviewer: I’ve asked a lot of questions, but I just wanted to check if there’s anything that I’ve missed that you think, “Nick should have asked me that?” Is there anything that you think I should have talked about?

Respondent: I don’t think there’s anything. One thing they usually ask is if it affects your relationships in any way and da-da-da. I think we treated that in one of that.

Interviewer: And in case we haven’t, do you feel that it affects your relationships?

Respondent: Then, not now. But, again, I’ll tell you one thing I’ve noticed with people – one thing I’ve noticed with people… I don’t know if it’s a generality or if it’s people here, but I’ve noticed that even with Nigerians living abroad, they feel they come into your life to do you a favour – that they have something and maybe not so many people will be willing to date you or not so many people will be willing to marry you. So they come and give you the impression that, okay, if they can’t… But always giving you that impression that, “I’m doing you a favour.” You understand that kind of thing? And that is one thing I keep talking to the ladies about – you cannot end up with somebody who thinks that he is doing you a favour. Your vitiligo might increase with childbirth or something, so if the person is not down with you as a person, the person should not be considered – it’s a very serious thing. Though they’ve challenged me

though. Three ladies with vitiligo have challenged me, if I’ve said that it doesn’t affect relationships, that I should get married. They’ve challenged me (laughs). So I told them, “Well, unfortunately, that is my personal life. I can sacrifice everything for vitiligo and I can sacrifice that just to make people happy. It will happen when I’m ready for that.” But I don’t think… Maybe because of my personality, I don’t really think it does affect it because, for some time, for 5 years, I was off it completely, I wasn’t interested in anybody – too much fickleness and… There were so many other things that was an issue for me – people not being real and all of that, so I just decided to stay away and just be on my own. But talking with other people, I find out it has affected… But, again, I try to get them to be… To accept themselves for who they are, so they will be able to check that, because no man wants somebody who is not confident. Likewise a woman – she doesn’t want anybody who is not confident. So if you are not confident, you’ll not find a person to say, “Okay, it can be you.” I can’t say it’s because of vitiligo because you may just be… Okay, I’ll give you an instance – there’s a lady (inaudible 00:52:05), she dated a man for 16 years because she believed that if the man goes, no other person will say hello to her. And for that 16 years, she was footing his bills, she was paying for his accommodation, she was doing everything for the man. The accommodation she was paying for, she didn’t know the place. The man did not allow her access to the place. And, every year, she would renew the rent for where the man is staying. She didn’t know any member of the man’s family. And she believes that, one day, the man will marry her. I have not met her in person but we have been talking on the phone for about three years. I broke that relationship on the phone. I had to break it because I didn’t understand why she was staying with the person. I told her, “No, it’s not that. Somebody else can come to you.” Eventually, somebody else came. They were in school together in (inaudible 00:52:54) school. And he was celebrating his 40th birthday and he went looking for her. But I wanted it to be out of this, so I called her and said, “If a man is celebrating his 40th birthday and he is looking for you that same day, I don’t think he is coming to you for some joke – I think he is serious. Be careful – don’t mess this one up again and all of that with your…” She said, “Okay” da-da-da. The next day, she was calling me to say that the man’s younger brother came and was asking her,

“What happened to you?” I said, “Because you are different, he is asking. Is there anything wrong in that?” She said no, that the man has no right to ask her what happened to her. I said, “Prepare – people always ask you.” I usually tell them if you don’t know what to explain, just tell them, “I have a pigmentation disorder – it’s a skin condition that turns black men white and white men whiter. If they don’t understand, give them my number, I will explain.” That is what I usually tell them – and some of them do this. But she took it… And she was angry. But, eventually, I called her again and said, “Respect yourself. Your self-worth is very important in whatever you are doing. If the man finds you to not be somebody who has self-respect or self-worth, he will not like you, he will not want you for keeps.” I told her this over time – and she messed it up. She called me… We didn’t talk for almost 9 months or so. She now called me and said there’s something happened that she felt I would be angry with her and all of that. “Okay. What happened?” She said she’s pregnant. “Oh, that’s a good thing. Why would I be…” Because, at the time, she was 41. I said, “You’re 41 and you’re pregnant. What do you want to do?” She said she wanted to terminate the pregnancy. Now, what will people say? What will people say about her? I said, “No, at your age, nobody will bother that you are pregnant. If it’s a girl of 21 or maybe less than 20, they might be saying, ‘Oh, okay. Why did it happen now? Why did he do that to her?’ About you, you were having unprotected sex with a man and you got pregnant and you are saying I should feel for you for what?” And the man told her plain, “Sorry, I don’t want to marry you” – but he actually came to marry her in the first place. You see what I’m saying? So that one, now, she’s saying that the man doesn’t want her because of vitiligo. I told her, “No, you messed it up. Because for him to come to you in the first place to celebrate his 40th, he wanted you for something good.”

Interviewer: So it’s the same thing again – there’s a theme, isn’t there? Be confident…

Respondent: Yes.

Interviewer: And don’t take second best because of vitiligo.

Respondent: Don’t take second best because of vitiligo. So, in all I’m saying, I can’t really say it affects relationships so much, because I don’t know how the other person is presenting herself to the person – it has a lot to do with it. But, in general, when I say it affects it, it’s because I know a man who left a wife with three kids because she suddenly started turning white and he said his nemesis is that she must have done something with her mother – that’s why she suddenly started turning white.

Interviewer: So he left her.

Respondent: He left her, yeah. But they now live in London, the lady and the three kids, and I think there’s a reconciliation with the husband or something. So it affects it if somebody can do that. And they are a very known family in the East, in River State – well-established family – so it’s not like it’s some unexposed person somewhere.

Interviewer: So it can have a very big impact on relationships…

Respondent: Yes.

Interviewer: But if you have the right attitude, then perhaps it has less of an impact.

Respondent: Yes. Especially when you’re not married – because when you are (inaudible 00:56:22), when you are married, you already… And it’s happening. Maybe you don’t know how to go about it, but make sure you sort it out before getting into it. You understand? I would prefer to have vitiligo before getting married, so that you know me, this is who I am, than it suddenly appears. People can be funny.

Interviewer: Okay. Thank you very much, (inaudible 00:56:41).

Respondent: You’re welcome.

Interviewer: I’m going to stop the recorder just now.

[End of Transcript]
