# Supplementary material for: The experience of living with vitiligo in Nigeria: A participatory Interpretative Phenomenological Analysis
Source: J Health Psychol. 2024 Jul 30;30(5):1120–35. doi: 10.1177/13591053241261684 (PMC11977828; doi:10.1177/13591053241261684)

Vitiligo study

Explanatory Memos

1. Example of reflective diary

‘Mrs X is an interesting participant. She was one of the older people taking part, one of the better educated (I think), in one of the better jobs and came from a high socioeconomic group (married to the uncle of the King of a tribe). By some distance she provided the most detail about the traditional belief structures of illness, such as the spirits and incorrect diet leading to vitiligo. However she also spoke confidently in the paradigms of medicine and Christianity. Like other participants she initially suspected that her vitiligo began as a result of some other medical thing, in her case giving birth. She presented as someone who is coping well and who has experienced less impact of from the vitiligo.

Throughout the interview she came across as a little evasive on questions of how vitiligo has impacted on her negatively. This could be for a number of reasons, perhaps she did not want to talk about it to a stranger, a man, a westerner, or in front on Ogo. I sometimes wonder what impact Ogo’s message had on the answers given by participants. Ogo takes the view that people should be confident and outspoken. That the future will be good. I think that this very positive mantra has a lot of advantages but that also it night get in the way of people acknowledging the hurt they feel. This hunch may be completely wrong’

The use of Interpretative Phenomenological Analysis (IPA)

IPA is the natural methodological choice to understand the experience of living with vitiligo in Lagos. It examines how people make sense of and engage in significant life experiences (Smith et al., 2009). Using careful interpretations the researcher draws on participants’ reflections and feelings about an event to bring meaning to an experience, this data is then considered in relation to the other interviews (Larkin & Thompson, 2012). This approach has previously been used to explore the experience of living with vitiligo by Thompson et al. (2002) and used widely in clinical health psychology (Brocki & Wearden, 2006). IPA has three core theoretical foundations: phenomenology, hermeneutics and idiography (Smith et al., 2009). Phenomenology is a philosophical position from which to study human experience. It seeks to understand the lived experience of being human, for instance regarding what it is like to be immersed in a particular environment, culture, language, or situation. Hermeneutics concerns the interpretation of experience. IPA involves a double hermeneutic, the participant is making sense of their experience and the researcher is attempting to make sense of what the participant is saying (Smith, 2011). Idiography is the systematic and thorough focus on the particular at a detailed level. It is the attempt to understand experiential phenomena using in-depth exploration of cases that are linked with one another (Smith et al., 2009).

TA, an approach that combines participant introspection with a structured form of promoting (Someren, Barnard, & Sandberg, 1994) is the most appropriate methodology to test the usability of a self-help leaflet (Haak et al., 2003). As an approach it can give insight into participants’ views and thought processes and has been widely used in the development of self-management programmes (Boren & Ramey, 2000). It has been argued that TA can provide valuable and reliable information about cognitive processes (Ericsson & Simon, 1980).

Process of data analyses

Part one - IPA. The interview transcripts were analysed in accordance with the methodology outlined by Smith et al. (2009) and Biggerstaff and Thompson (2008). For each participant the transcribed audio recordings were checked for accuracy by the researcher. Notes were made in the left-hand column of initial reflections and thoughts during the first encounter with the text (see below). The researcher referred to the reflective diary (see below) to help contextualize the experience, nature, and origin of the interpretations.

Line-by-line the transcriptions were organized into a spreadsheet according to preliminary-themes (Appendix L). These themes attempted to capture the essence of the interview and the researchers’ interpretation of it. Once the entire transcript was coded into the spreadsheet the themes were reorganized into clusters. These clusters resulted in super-ordinate themes. So as to make the super-ordinate themes and sub-themes more visually accessible the spreadsheet was converted into a mind-map (see additional data analysis file for this step).

1. Example of IPA text notes in the left hand column reflecting initial thoughts


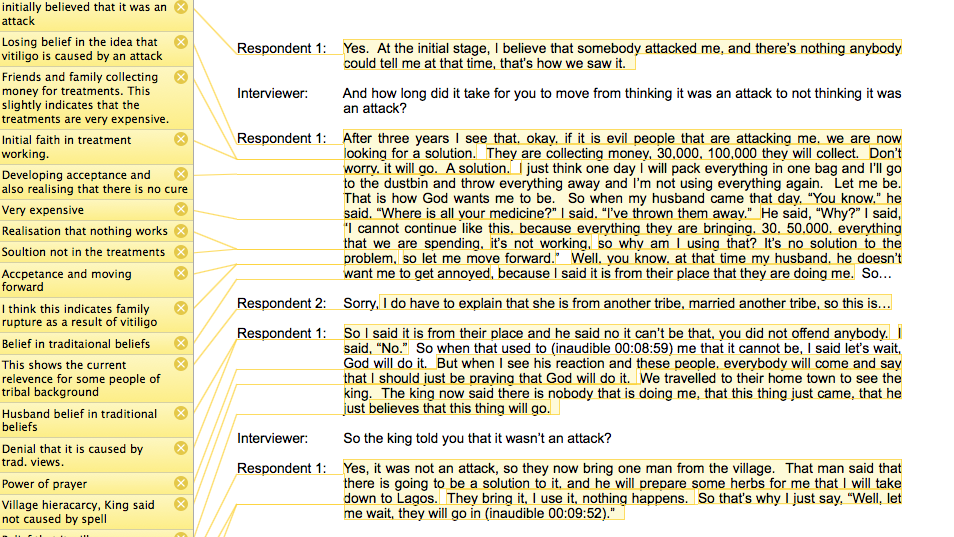


1. Example from spreadsheet of preliminary IPA themes
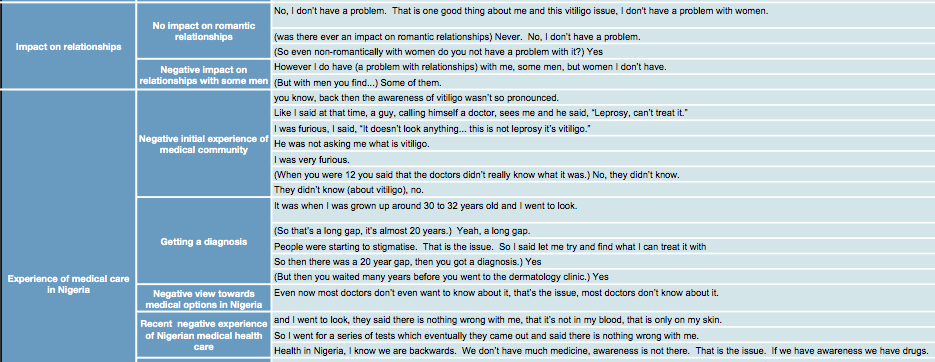

Supplement: sj-docx-3-hpq-10.1177_13591053241261684 – Supplemental material for The experience of living with vitiligo in Nigeria: A participatory Interpretative Phenomenological Analysis [file sj-docx-3-hpq-10.1177_13591053241261684.docx]
