# Supplementary material for: The experience of living with vitiligo in Nigeria: A participatory Interpretative Phenomenological Analysis
Source: J Health Psychol. 2024 Jul 30;30(5):1120–35. doi: 10.1177/13591053241261684 (PMC11977828; doi:10.1177/13591053241261684)
Supplement: sj-docx-4-hpq-10.1177_13591053241261684 – Supplemental material for The experience of living with vitiligo in Nigeria: A participatory Interpretative Phenomenological Analysis [file sj-docx-4-hpq-10.1177_13591053241261684.docx]

Read ME

1. Upload info
2. Document with the manuscript presenting the main research conducted
3. Document with the title page with details of the co-authors and other information about the study
4. Document with the table 1 (only one table)
5. Document with the supplementary Materia (semi-structure guide)
6. Document with the data set (transcripts from the interviews conducted)
7. Document with the explanatory memo (presenting details about the data analyses)
8. Document with the data analyses details (robustness of analyses)
