# Supplementary material for: The experience of living with vitiligo in Nigeria: A participatory Interpretative Phenomenological Analysis
Source: J Health Psychol. 2024 Jul 30;30(5):1120–35. doi: 10.1177/13591053241261684 (PMC11977828; doi:10.1177/13591053241261684)

Supplementary Table S1: Summary of participant demographic, quality of life, and social anxiety scores

| **Participant psuedonyms** | **Age** | **Gender** | **Profession** | | **Quality of life (DLQI) Score** | **Social anxiety (FNE Score** | | **Age vitiligo first noticied** | | |
| --- | --- | --- | --- | --- | --- | --- | --- | --- | --- | --- |
| **HN** | 38 | F | | Head of charity | 2- Small effect | | 8- Low | | 30 |  |
| **PL** | 28 | F | | Cinematographer | 11- Very large effect | | 15- Average | | 25 |  |
| **NT** | 62 | M | | Engineer | 1- Small effect | | 10- Low | | 31 |  |
| **AT** | 39 | F | | Officer Worker | 2- small effect on life | | 13- Average | | 30 |  |
| **MP** | 35 | M | | Web Designer | 4- Small effect | | 15- Average | | 22 |  |
| **RY** | 29 | F | | Hospitality worker | 3- Small effect | | 14- Average | | 10 |  |
| **KO** | 32 | M | | Primary teacher | 14- Very large effect | | 15- Average | | 21 |  |
| **DD** | 41 | M | | Member Sailor | 12- Very large effect | | 15- Average | | 12 |  |

Supplementary Material S2: A semi-structured schedule


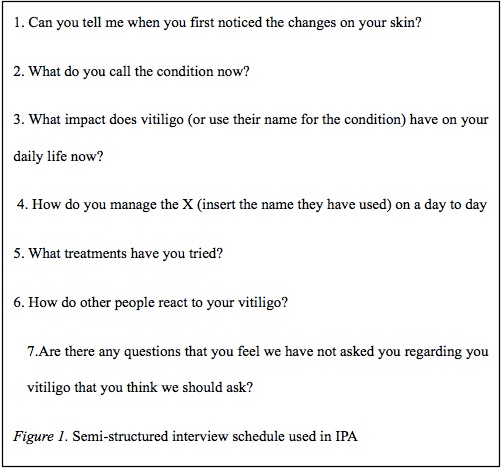


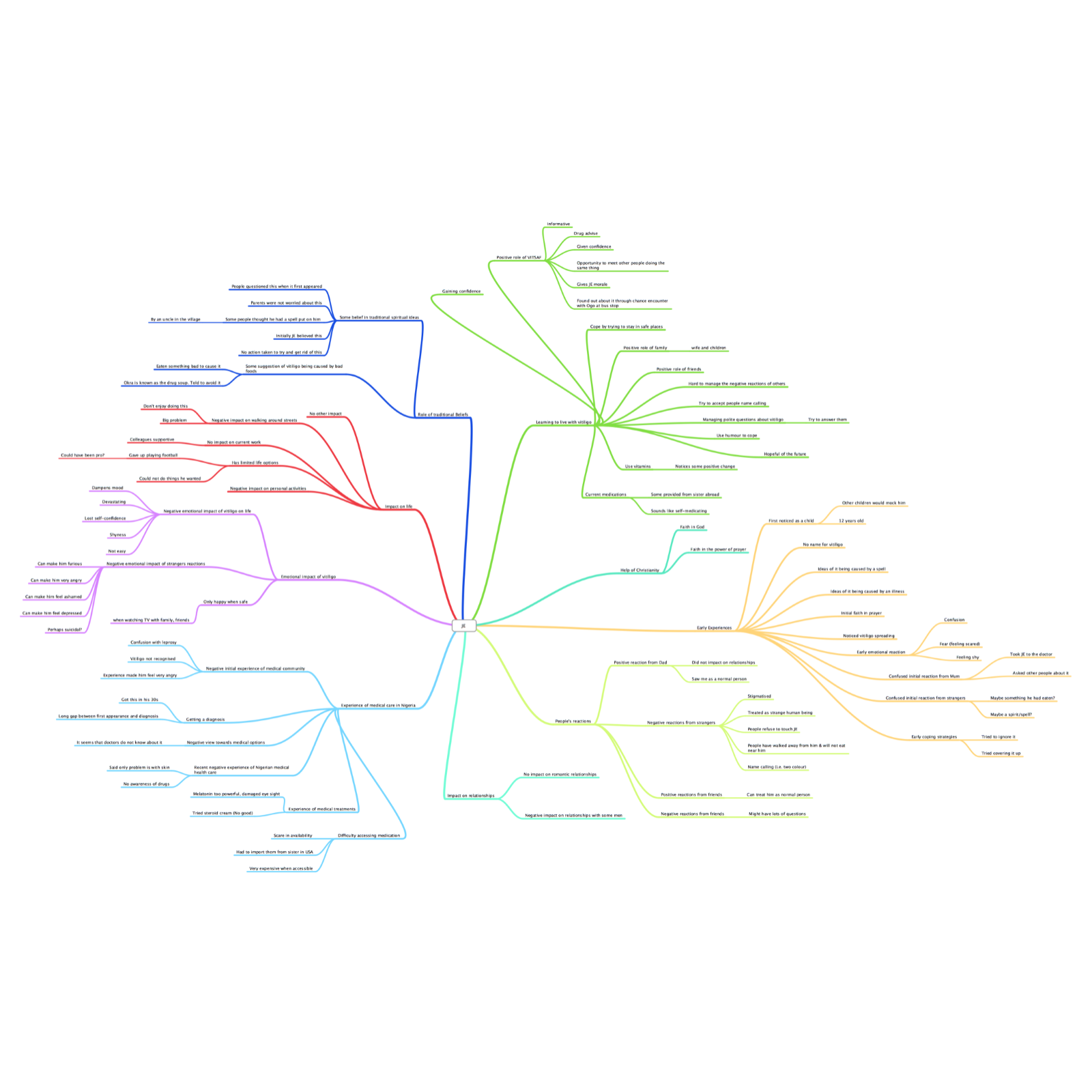
Supplementary Material S3: Example of mind map of IPA themes

 Flow diagram used to facilitate IPA


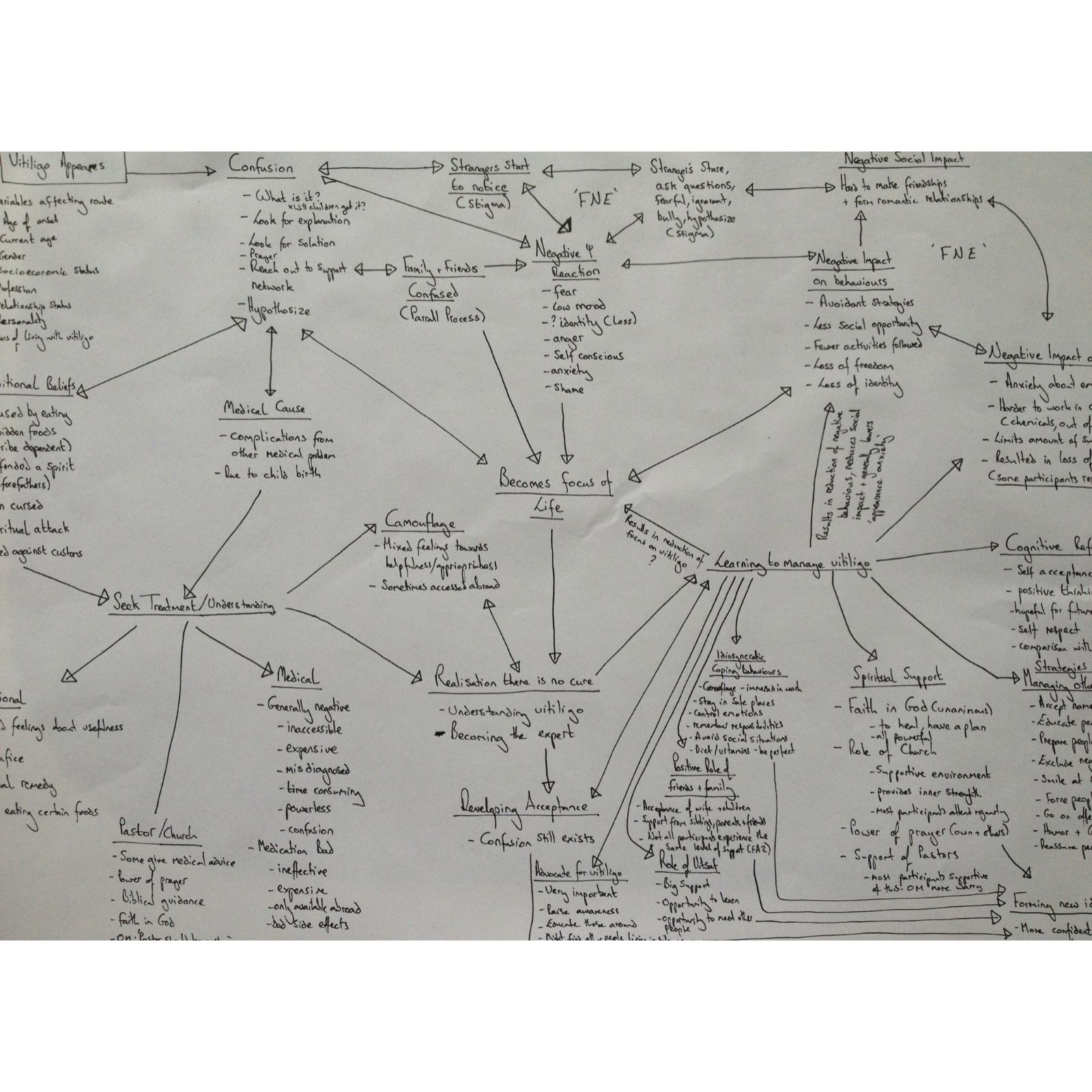


Example of TA initial coding


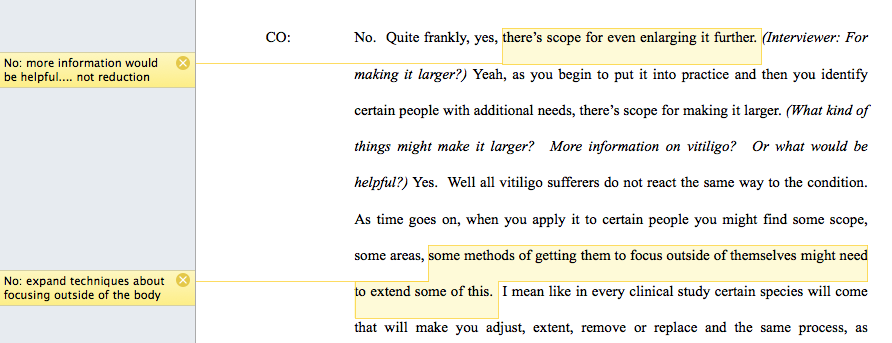


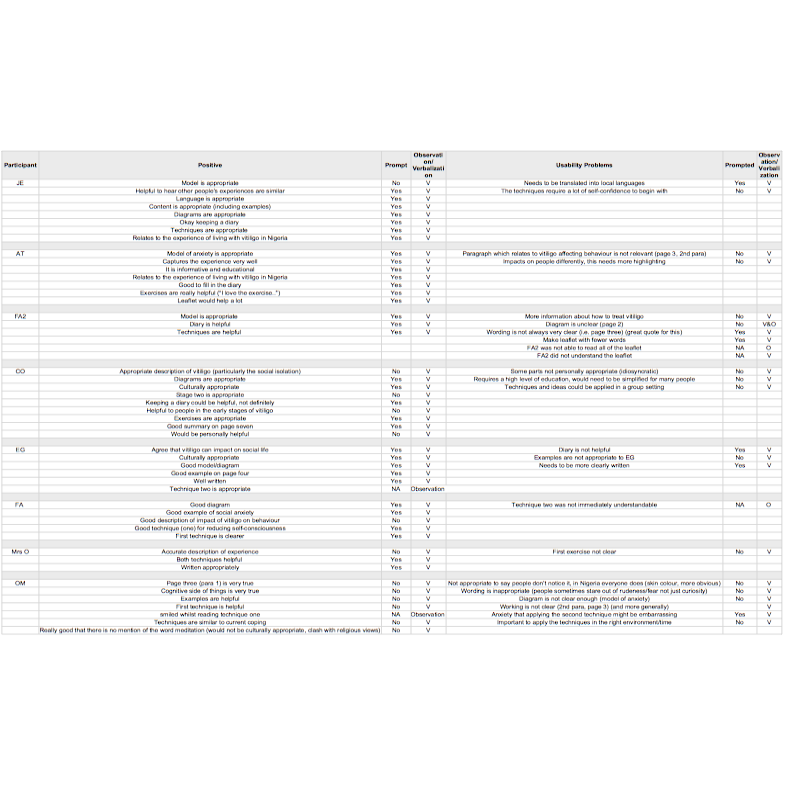
Spreadsheet used to facilitate analysis

Supplementary Material S4: Eight-point guideline for conducting research in developing countries as outlined by Emanuel, Wendler, Killen and Grady (2003)
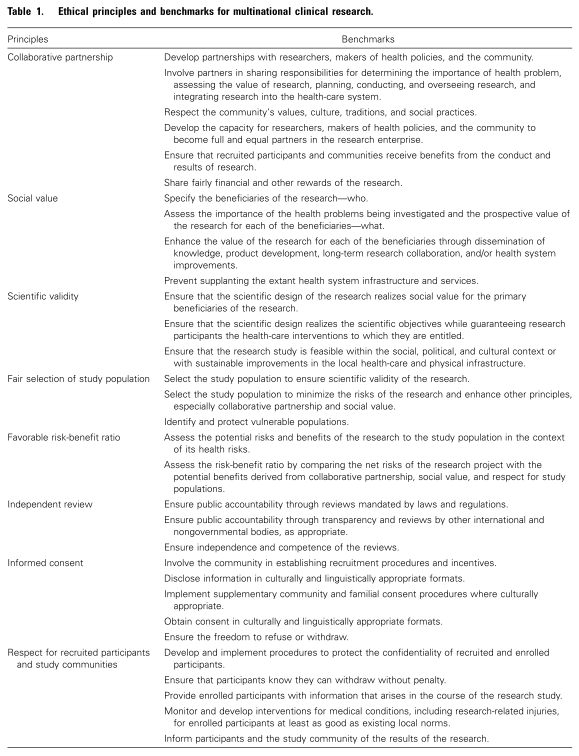

Supplement: sj-docx-5-hpq-10.1177_13591053241261684 – Supplemental material for The experience of living with vitiligo in Nigeria: A participatory Interpretative Phenomenological Analysis [file sj-docx-5-hpq-10.1177_13591053241261684.doc]
